# The most effective therapeutic exercises for pain intensity in women with fibromyalgia: A systematic review and network meta-analysis^☆^

**DOI:** 10.1016/j.bjpt.2025.101226

**Published:** 2025-05-03

**Authors:** Álvaro-José Rodríguez-Domínguez, Manuel Rebollo-Salas, Raquel Chillón-Martínez, Abel Rosales-Tristancho, Inmaculada Villa-del-Pino, José-Jesús Jiménez-Rejano

**Affiliations:** a Department of Health and Sports, Pablo de Olavide University, Seville, Spain; b Department of Physical Therapy, University of Seville, Seville, Spain; c Department of Applied Economics I, University of Seville, Seville, Spain

**Keywords:** Fibromyalgia, Pain, Physical therapy, Rehabilitation, Therapeutic exercise

## Abstract

**Background:**

Therapeutic exercise (TE) is the only intervention with strong recommendation for fibromyalgia. However, there is controversy as to which type of exercise is the most beneficial.

**Objective:**

To determine which TE approach is the most effective in reducing pain intensity in women with fibromyalgia.

**Methods:**

A systematic review was performed with a network meta-analysis (NMA). Six databases were searched from inception until January 2024. Randomized controlled trials (RCTs) evaluating the effects of TE on pain intensity were included in women with fibromyalgia. Methodological quality was assessed using the Physiotherapy Evidence Database scale. The size of the effect and the clinically important difference were determined in the short-term (≤3 months) and long-term (>3 months).

**Results:**

Sixty-one studies were identified, of which 51 were included in the quantitative synthesis (*n* = 2873). Fifteen TE interventions and eight comparison interventions (comparators) were identified. Aquatic exercise (p-score: 0.8713) was found to provide best benefits in the short-term and resistance training in the long-term (p-score: 0.9749). Statistically significant differences were found in favor of aquatic exercise, Pilates, qigong, resistance training, virtual reality, mixed exercise, and aerobic exercise (in the short-term) and in favor of resistance training, dance, functional training, aquatic exercise, virtual reality, and aerobic exercise (in the long-term) compared to usual care.

**Conclusion:**

With a moderate level of evidence, our NMA shows that, in the short-term, aquatic exercise is the most effective TE intervention to reduce pain intensity in women with fibromyalgia, while resistance training is the most effective in the long-term. More RCTs are needed to strengthen these findings.

## Highlights

• Resistance training was the only therapeutic exercise (TE) approach that showed clinical relevance, in the short and long term, to reduce pain intensity in women with fibromyalgia, showing a moderately important improvement (>30 %).
• In the short term, aquatic exercise was the most effective approach to reduce pain intensity, so it could be an appropriate option to start a TE program in women with fibromyalgia.
• These findings could contribute to a change in the current paradigm on TE prescription in fibromyalgia, because the “gold standard” recommendation of aerobic exercise should be replaced by other more effective approaches.
• The results provide a valuable tool for decision making when choosing the most appropriate TE approach according to the patient's characteristics and preferences, because it is a key factor in improving adherence to treatment.

## Introduction

Fibromyalgia is a chronic syndrome characterized by widespread musculoskeletal pain, chronic fatigue, sleep disturbances, and physical disability.^1^, ^2^, ^3^, ^4^ It is prevalent in 2 to 4 % of the world's population,^2^, ^3^, ^4^, ^5^, ^6^ affecting mainly women.^5^^,^^7^^,^^8^ Although it is hypothesized that fibromyalgia is due to a central sensitization process, the etiopathological mechanisms are still unclear.^3^^,^^9^, ^10^, ^11^, ^12^ Studies have identified several muscle abnormalities, such as a reduction in type II fibers and alterations in muscle contraction and metabolism, which can contribute to fatigue and pain in patients with fibromyalgia.^13^^,^^14^ These findings may explain the benefits of physical exercise for these patients.

Physical exercise is the only treatment with a strong recommendation for fibromyalgia.^2^^,^^3^ Several reviews have evaluated the effects of different therapeutic exercise (TE) approaches in patients with fibromyalgia, with benefits observed in almost all of them.^15^, ^16^, ^17^, ^18^, ^19^, ^20^, ^21^, ^22^ However, there is controversy as to which approach is the most beneficial. The Cochrane review^23^ of 2007 classified aerobic exercise as the "gold standard" for the treatment of fibromyalgia, although in their 2017 review,^18^ they concluded that this exercise approach may have little or no long-term effect on pain.

To date, we have not found any publications that compares all TE approaches with each other for the treatment of pain in fibromyalgia. Because the disease affects mainly women and the response to treatment is influenced by a multitude of factors, including gender, the objective of this study was to analyze the efficacy of different TE approaches in pain intensity in women with fibromyalgia and to identify which exercise approach is the most effective through a network meta-analysis (NMA) of randomized clinical trials (RCTs).

## Methods

A systematic review with an NMA following the PRISMA-NMA guidelines.^24^ The protocol was registered in the PROSPERO database.

Our search was conducted from database inception until January 14, 2024. The databases selected were MEDLINE, CENTRAL, Embase, Web of Science (WoS), Cumulative Index to Nursing and Allied Health Literature (CINAHL), and Scopus. The search strategy and keywords used are given in Supplementary material (Table S.1)

To minimize publication bias, a search was performed on ClinicalTrials.gov. Additional records were searched by hand from relevant literature reviews to supplement the findings of the database.

### Eligibility criteria

The inclusion criteria followed the PICOS (participants, interventions, comparators, outcomes, study design) strategy:

1. Type of study: RCTs.
2. Participants: adult women diagnosed with fibromyalgia according to the criteria of the American College of Rheumatology for fibromyalgia (ACR 1990/2010/2016).
3. Interventions: studies that included any form of TE as the only intervention or a combination of exercises in any of the groups were selected.
4. Comparators: any treatment
5. Outcomes: pain intensity, evaluated with visual analog scale (VAS 0–10, 0–100)

Trials published in languages other than English, Spanish, French, Italian, or Portuguese were excluded, as were trials that combined ET with other treatments and trials that included men.

### Data collection process

Two review authors (AR-D, JJ-R) independently performed study selection and data extraction. A third author (MR-S) was consulted in case of disagreement. Studies that did not report the data required for the meta-analysis were excluded from the quantitative synthesis. In general, the data collected were the mean and standard deviation for each treatment and period studied. Given the existence of studies that provided other types of measurements (such as median or quartiles Q1 and Q3), prior estimates were made to obtain an approximation of the mean and standard deviation from the data.^25^^,^^26^

A standardized form was used for data extraction, addressing participants, diagnostic criteria, type of intervention, follow-up time, and results obtained (Supplementary material -Table S.2).

### Risk of bias

The methodological quality was evaluated using the PEDro scale.^27^ The quality of the studies was reviewed by two independent evaluators (IV-P, RC-M), and a third evaluator (AR-T) was consulted when discrepancies appeared. The included studies were classified according to scores of 9 or 10, 6 to 8, and ≤ 5 on the PEDro scale and were interpreted as excellent, good, and fair quality, respectively.^28^

### Certainty of the evidence

The CINeMA web application was used to assess confidence in findings from primary NMA. The CINeMA framework considers six domains that affect the level of confidence in the NMA results: (a) within-study bias, (b) reporting bias, (c) indirectness, (d) imprecision, (e) heterogeneity, and (f) incoherence. The reviewers assessed the level of concern for each relative treatment effect of NMA as giving rise to "no concerns", "some concerns", or "major concerns" in each of the six domains. Then, judgments across the domains are summarized into a single confidence rating ("high", "moderate", "low", or "very low").^29^ (Supplementary material -Table S.3)

### Clinically important differences (CID)

According to Cochrane,^16^ the Initiative on Methods, Measurement and Pain Assessment in Clinical Trials (IMMPACT) recommended the following benchmarks for interpreting changes in pain intensity on a numerical rating scale 0 to 10 in chronic pain clinical trials: a) a 10 to 20 % decrease is minimally important, b) a decrease greater than 30 % is moderately important, and c) a decrease greater than 50 % is substantial.^30^^,^^31^ Therefore, the minimum CID in outcome was interpreted as a pain difference of 15 points out of 100 (VAS 0–100). The CID was determined from the difference obtained for each intervention compared to usual care.

### Summary measures

The analysis included a qualitative synthesis (Supplementary material -Table S.2) and a quantitative synthesis (pairwise meta-analysis and NMA). VAS is reported as a continuous measure from 0 to 10 and from 0 to 100. The outcome data was standardized from 0–100, using the mean difference (MD) to perform the primary analyzes. A temporal division was established into two periods: short-term (≤ 3 months) and long-term (> 3 months). For the short term, only follow-up results closer to 3 months after the end of the intervention were selected. For the long term, only follow-up results closer to six months after the end of the intervention were selected.

In those meta-analyses, a random-effects model was used where heterogeneity between studies was observed, while a fixed-effects model was used for those where homogeneity was observed. As indicative of homogeneity, I^2^ coefficient values <50 % and/or Chi^2^ test values of homogeneity with *p* > 0.05 were taken. In all cases, the corresponding forest plot is presented (Supplementary material -Figs. 2–14). A pairwise meta-analysis was performed using Review Manager software version 5.4.

Regarding NMA, the means provided by each study were taken for each of the treatments performed, as well as the standard deviation of each one, which allowed the calculation of the MD, as well as its standard error.^32^ The NMA allows us to know the effect size between any pair of treatments, whether they have been compared directly or not. To establish a ranking between all treatments, it is possible to assign a p-score to each treatment. This score is based on the probability that a treatment is better than any other treatment included in the NMA.^33^, ^34^, ^35^

### Planned methods of analysis

The NMA developed in this study is framed within the framework of frequentist statistics.^36^^,^^37^ NMA is performed in RStudio using the ‘netmeta’ library.^38^

Coherence is the statistical tool to test the transitivity between treatment effects.^33^ The objective is to study whether there are statistically significant differences between direct and indirect comparisons of any two treatments. For this purpose, the SIDE (Separating Indirect from Direct Evidence) method is used to study local coherence and the I^2^ statistic is used in the case of global coherence.^33^^,^^39^^,^^40^ This statistic is calculated from Cochran’s Q.^40^

The NMA is developed according to a random-effects model that considers the heterogeneity of the estimates. Furthermore, the random-effects model facilitates extrapolation of the results obtained to a larger population.^39^^,^^41^

## Results

**Fig. 1:**
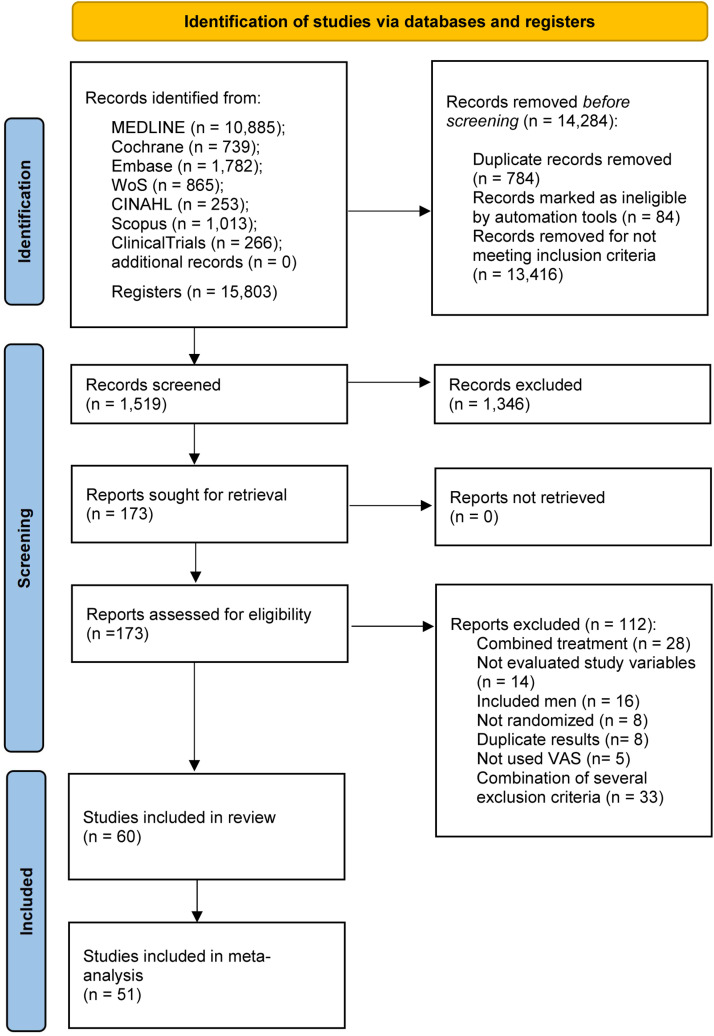
Flow chart of the selection of studies.

Fig. 1 describes the study selection process. Sixty articles were included in the qualitative synthesis^42^, ^43^, ^44^, ^45^, ^46^, ^47^, ^48^, ^49^, ^50^, ^51^, ^52^, ^53^, ^54^, ^55^, ^56^, ^57^, ^58^, ^59^, ^60^, ^61^, ^62^, ^63^, ^64^, ^65^, ^66^, ^67^, ^68^, ^69^, ^70^, ^71^, ^72^, ^73^, ^74^, ^75^, ^76^, ^77^, ^78^, ^79^, ^80^, ^81^, ^82^, ^83^, ^84^, ^85^, ^86^, ^87^, ^88^, ^89^, ^90^, ^91^, ^92^, ^93^, ^94^, ^95^, ^96^, ^97^, ^98^, ^99^, ^100^, ^101^ and 51 were included in the quantitative synthesis (Supplementary material -Table S.2).

### Characteristics of the study and results of individual studies

All studies were published between 1992 and 2021. Three studies^102^, ^103^, ^104^ had two treatment arms with the same exercise approach but different doses, so they were counted as six studies in quantitative synthesis.

The sample size in each article was between 15 and 207, with an average of 59 participants. The total number of participants was 3581, of which 2873 were included in the quantitative synthesis (708 not included). The mean age was approximately 48.9 ± 6.1 years (range = 32–59 years). Of the 60 studies included, 48 used the ACR1990 criteria, 13 the ACR2010 criteria, and 2 the ACR2016 criteria. The total number of studies and participants per exercise modality, the total number of participants per nationality and intervention method, and the diagnostic criteria used are detailed in Table S.4 (Supplementary material).

### Intervention group (Therapeutic exercise)

The interventions were classified according to the definitions given in Table S.5 (Supplementary material) and described according to the exercise approach used, the program design, and the dosage (intensity, frequency, and duration) (Supplementary material -Table S.2). . One of the identified categories (vibration exercise) was only investigated in one study.^105^ However, it did not provide the necessary data to be included in the meta-analysis, so it was only included in the qualitative synthesis. Thus, of the 15 TE categories identified, 14 were included in the NMA.

### Comparison group (comparator intervention)

Eight intervention categories were identified as comparison groups in the included studies (Supplementary material -Table S.5). Studies that combined exercise therapy with another treatment approach were classified as "Combined Therapy" and therefore qualified as a comparison group. Studies that included a placebo treatment were classified as “sham” and those in which no intervention or usual care was performed as "usual care".

### Outcomes

The main analyzes of this study evaluated the effect of the different identified TE approaches on pain intensity in women with fibromyalgia, assessed with VAS. All available results were collected for the available follow-up time points, with two-time cut-off points for the primary meta-analyzes: short-term (≤3 months) and long-term (>3 months).

### Synthesis of results

The network formed by the treatments and their comparisons is constructed from a graph where each node represents a treatment and each edge a direct comparison.^106^ The size of the nodes is proportional to the sample collected for each treatment. However, the thickness of each edge is proportional to the number of comparisons collected between the two treatments at its ends.

### Pain results

**Fig. 2:**
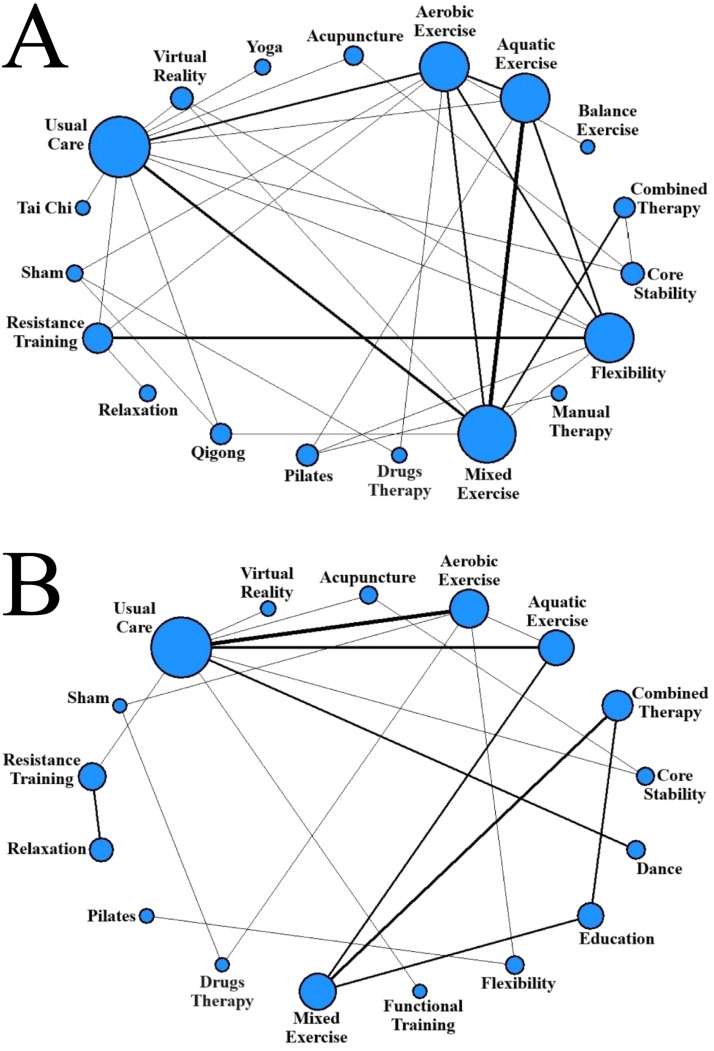
Network plot presenting the trial data contributing evidence comparing exercise treatment types. A: Short-term (≤3 months): 37 trials, 171 comparisons, 1922 participants. B: Long-term (>3 months): 24 trials, 136 comparisons, 1530 participants. The size of the nodes represents how many times the exercise appears in any comparison about that treatment and the width of the edges represents the total sample size in the comparisons it connects.

For the short-term (Fig. 2A), a total of 19 interventions were analyzed by 37 different studies (35 studies plus two studies that had two treatment arms of the same intervention). Among them, there were 32 pairwise comparisons and five comparisons between the three intervention groups at the same time. Usual care had the largest sample size (*n* = 307), followed by mixed exercise (*n* = 290), aquatic exercise (*n* = 236), flexibility (*n* = 234), and aerobic exercise (*n* = 233). The thickness of the edges determines that there are a greater number of comparisons between aquatic exercise and mixed exercise (4 comparisons) and between flexibility and resistance training (3 comparisons).

Regarding transitivity, the SIDE method did not show statistically significant differences between direct and indirect comparisons of treatments (Supplementary material -Table S.6A). Furthermore, the I^2^ statistic had a value of 84.7 % (78.5 %, 89.1 %).

**Fig. 3:**
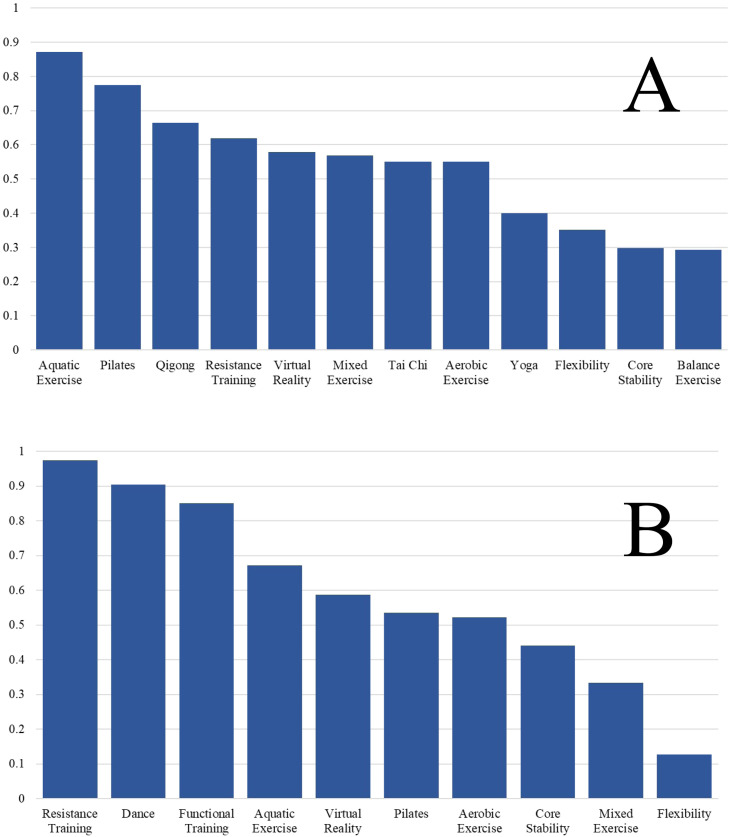
Treatment rankings and surface under the cumulative ranking curve (SUCRA) for pain intensity. A: Short-term (≤3 months); B: Long-term (>3 months).

Fig. 3 shows the ranking between all treatments. The p-score represented for each TE intervention in Fig. 3A places the aquatic exercise group in the first position (with a value of 0.8713), followed by Pilates (0.775).

**Fig. 4:**
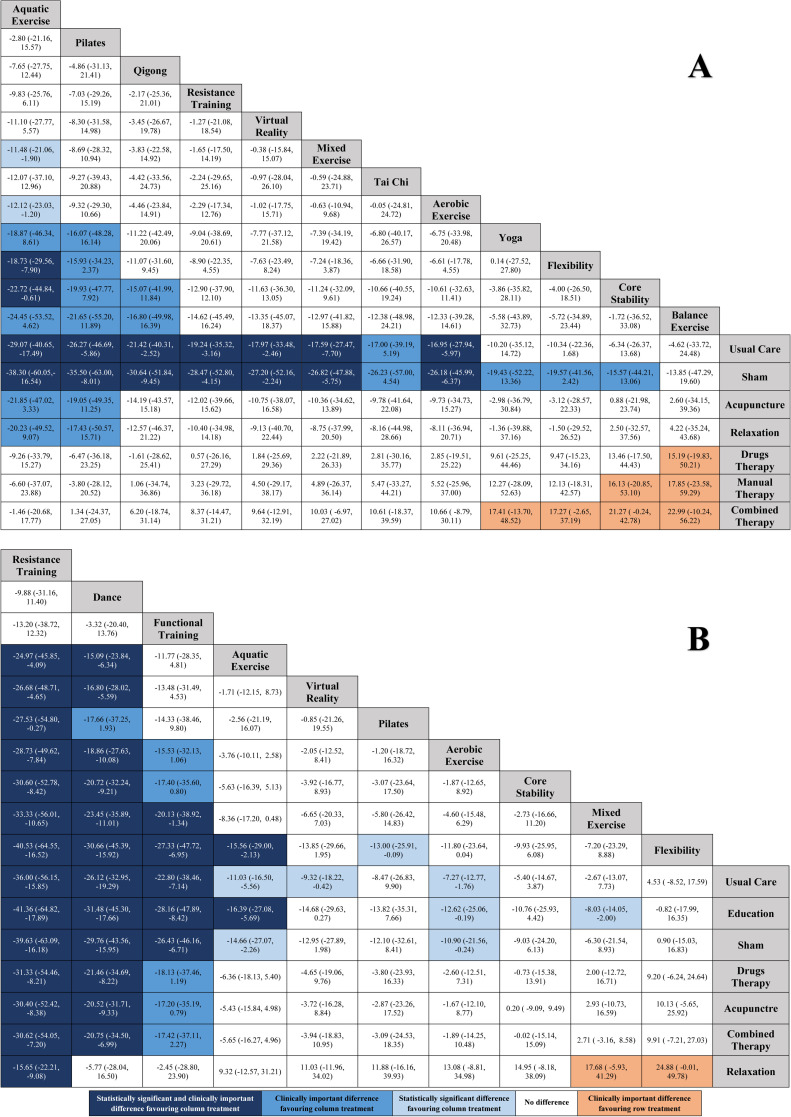
League table of network meta-analysis results for all comparisons between exercise and non-exercise interventions. Effects are expressed as the mean difference (95 % CI) between interventions. A: short-term outcomes (≤3 months); B: long-term results (>3 months).

Fig. 4 shows the relative effects between all pairs of comparisons and their respective confidence intervals at the 95 % level. A negative value of the effect size indicates the superiority of the column treatment, while a positive value indicates the superiority of the row treatment. The order of the columns in Fig. 4A was constructed following the ranking established in Fig. 3A.

**Fig. 5:**
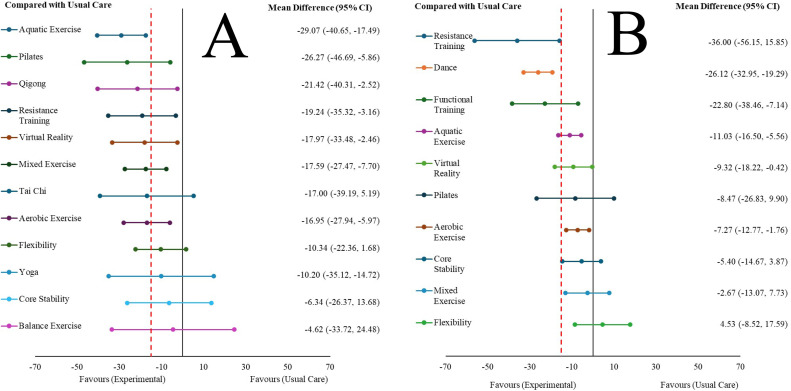
Summary network meta-analysis results for each exercise type compared with Usual Care. A: short-term (≤3 months); B: long term (>3 months). The hashed line indicates clinically important difference.

Statistically significant differences were found in favor of aquatic exercise, Pilates, qigong, resistance training, virtual reality, mixed exercise, and aerobic exercise compared to usual care and sham. Aquatic exercise also obtained statistically significant differences in comparisons with mixed exercise, aerobic exercise, flexibility, and core stability. Taking the usual care as a reference, it is possible to observe the effect size with respect to the other interventions in Fig. 5A.

Finally, 12 short-term pairwise meta-analyses were also performed (Supplementary material -Figs. S1-S6 and S.9-S14), of which five TE approaches showed statistically significant results in favor of aquatic exercise (*p* < 0.0001), Tai Chi (*p* < 0.00001), virtual reality (*p* = 0.001), resistance training (*p* = 0.02), and qigong (*p* = 0.02). On the other hand, an intervention (flexibility) had statistically significant differences in favor of the comparison group, with a p-value <0.005 (*p* = 0.002). All results are summarized in Table S. 7 (Supplementary material).

For the long-term (Fig. 2B), there were 17 interventions evaluated by 24 studies (22 studies, and two more with one pair of treatment arms each). Of the 24 studies, 20 made pairwise comparisons and four presented comparisons between three treatments at the same time. Node thickness again indicates that the usual care group has the largest sample size (*n* = 312), followed by the aerobic exercise (*n* = 177), mixed exercise (*n* = 164), and aquatic exercise (*n* = 156) groups. Furthermore, the number of comparisons between the usual care and aerobic exercise groups was the highest, with a total of four between all studies, which resulted in a higher thickness on the corresponding edge in Fig. 2B.

Regarding transitivity, the net splitting method also did not show statistically significant differences between direct and indirect comparisons of treatments (Supplementary material -Table S.6B). Furthermore, the I^2^ statistic had a value of 0 % (0 %, 56.6 %).

The p-score represented for each TE intervention in Fig. 3B places resistance training first (0.9749), followed by dance (0.905) and functional training (0.8502).

The order of the columns in Fig. 4B was also constructed following the ranking established in Fig. 3B.Fig. 4B shows that a greater number of statistically significant comparisons were obtained in the long term than in the short term. Thus, we can highlight that statistically significant differences were found in favor of resistance training, dance, functional training, aquatic exercise, virtual reality, and aerobic exercise compared to usual care, and statistically significant differences were found in favor of resistance training, dance, functional training, aquatic exercise, aerobic exercise, and mixed exercise compared to education. Furthermore, resistance training showed statistically significant differences in all comparisons, except compared to dance and functional training. Fig. 5B shows the effect size using usual care as a reference group.

Finally, 10 pairwise meta-analyses were performed in the long-term (Supplementary material -Figs. S.1-S.9 and S.13), of which six TE approaches showed statistically significant results in their favor: resistance training (*p* < 0.0001), aquatic exercise (*p* = 0.0002), dance (*p* = 0.003), functional training (*p* = 0.004), aerobic exercise (*p* = 0.008), and virtual reality (*p* = 0.04). As in the short-term, flexibility had statistically significant differences in favor of the comparison group, with a p-value <0.005 (*p* = 0.003). All results are summarized in Table S.7 (Supplementary material).

### Clinically important differences (CID)

Fig. 5 shows the CID of the interventions analyzed compared to usual care. In the short-term (Fig. 4A), CIDs were obtained for many TE approaches (aquatic exercise, Pilates, qigong, resistance training, virtual reality, mixed exercise, Tai Chi, and aerobic exercise). However, in the long term (Fig. 4B), only three interventions achieved a CID: resistance training, dance, and functional training. Furthermore, only resistance training showed a moderately important clinical difference (≥30 points out of 100). Finally, in Fig. 4A and 4B, some comparisons between TE approaches and the comparators presented CID in favor of the comparators.

### Risk of bias

Most studies (48 out of 60) were rated as "good quality" on the PEDro scale. Seven studies were rated as "excellent quality" and five studies were rated as "fair quality" (Supplementary material -Table S.8).

### Publication bias risk assessment

The Begg and Egger tests did not reveal statistical evidence of publication bias (*p* > 0.05). These findings are shown in the funnel plots (Supplementary material -Fig. S.15-S.28). The sensitivity analysis indicated that the general results were not substantially modified by eliminating any result.

### Certainty of the evidence

In the short-term, 171 comparisons (47 studies) were performed, of which 22 were rated as “moderate quality”. The remaining 149 comparisons were rated as "low quality". Long-term, 137 comparisons (32 studies) were performed, of which only one was rated as "high quality" (aerobic exercise versus usual care). Of the remaining 136 comparisons, 110 were rated as "moderate quality" and 26 as "low quality". (Supplementary material -Table S.98).

## Discussion

To our knowledge, this is the first NMA to compare different TE approaches used in women with fibromyalgia to reduce pain intensity. Our NMA established a ranking (Fig. 3) of TE approaches that we used to order the columns in Fig. 4 showing the relative effects between all pairs of comparisons. Therefore, Fig. 4 can be used as a valuable decision-making tool when choosing the most appropriate type of TE to reduce pain intensity in women with fibromyalgia.

In the short-term (Fig. 4A), we had seven TE approaches that generated statistically significant differences compared to usual care and sham; also, these improvements were clinically important. If we analyze the comparisons between these seven approaches, our results appear to indicate that, in the short term, the five most effective type of TE to improve pain intensity in women with fibromyalgia were aquatic exercise, Pilates, qigong, resistance training, and virtual reality. Furthermore, the strength of evidence, in all these comparisons with usual care was moderate, except for Pilates, which was low.

In the long-term (Fig. 4B), we had six TE approaches that generated statistically significant differences compared to usual care and education. Compared to sham, five types of TE generated statistically significant differences. If we analyze the comparisons between these exercise approaches, our results appear to indicate that, in the long-term, the three most effective type of TE, compared to usual care, education, and sham, to improve pain intensity in women with fibromyalgia, were resistance training, dance, and functional training. Furthermore, resistance training and dance were the most effective TE compared to drug therapy, acupuncture, and combination therapy; and only resistance training was the most effective TE compared to relaxation. All of these improvements were clinically important. The strength of evidence, of all these comparisons was moderate.

Importantly, resistance training was the only intervention that showed statistically significant and clinically relevant differences compared to usual care and sham in both the short and long term. It was also the only intervention that showed a moderately important clinical difference (≥30 points out of 100), so, overall, resistance training may be the most effective type of TE to reduce pain intensity in women with fibromyalgia.

Our findings have significant clinical implications for several reasons. Most importantly, Fig. 4 can serve as a valuable guide for selecting the most appropriate TE approaches to reduce pain intensity in women with fibromyalgia. This decision should also be made based on the patient's characteristics and preferences, as this is a key factor in improving adherence to treatment.^107^, ^108^, ^109^ These findings could also contribute to a change in the current paradigm regarding TE prescription in fibromyalgia, because the "gold standard" recommendation of aerobic exercise should be replaced by other more effective approaches. Specifically, aquatic exercise was more effective than aerobic exercise in the short-term and resistance training and dance were more effective in the long-term.

This review has several limitations. First, studies are at risk of bias due to the impossibility of blinding when active interventions are used. Second, this review is limited to the effects of exercise in women with fibromyalgia. It seems more appropriate to limit the sample to this sex, as fibromyalgia affects mainly women and including men in the review would increase the risk of bias. Therefore, these results cannot be extrapolated to men with fibromyalgia. Third, there are concerns about the patient selection criteria used in RCTs. A total of 80 % of the trials used the ACR 1990 criteria, the validity of which was refuted by the authors themselves in 2010. Despite this, studies published in 2021 still continue to use these criteria.^110^, ^111^, ^112^ This could lead to serious selection bias in patients, which could affect the extrapolation of the results to the clinical setting. Finally, our study has evaluated the efficacy of different TE approaches in reducing pain intensity. However, it should be noted that intensity is only one dimension of pain experience, and therefore an improvement in pain intensity does not necessarily imply an improvement in the overall impact of the disease.^19^

Future studies should focus on whether different parameters of the FITT model (frequency, intensity, time, and type), could optimize outcomes in reducing pain intensity in women with fibromyalgia. Finally, it is imperative to unify diagnostic criteria in fibromyalgia so that these patients are classified more accurately and homogeneously. The criteria currently used for the diagnosis of fibromyalgia are still based on a biomedical model, ignoring the recommendations of the World Health Organisation (WHO). Psychosocial variables are known to have a significant impact on disease development, so it is necessary to establish diagnostic criteria based on a biopsychosocial model.^113^

## Conclusions

The NMA showed, with a moderate level of evidence, that the most effective TE approach to reduce pain intensity in women with fibromyalgia was, in the short-term, aquatic exercise and, in the long-term, resistance training. Furthermore, resistance training was the only intervention that showed short- and long-term improvements, with a moderately important clinical difference. More RCTs are needed to strengthen these findings.

## Declaration of competing interest

The authors declare no competing interest.

## Appendix Supplementary materials

Image, application 1
