# Supplementary material for: The most effective therapeutic exercises for pain intensity in women with fibromyalgia: A systematic review and network meta-analysis
Source: Braz J Phys Ther. 2025 May 3;29(4):101226. doi: 10.1016/j.bjpt.2025.101226 (PMC12099910; doi:10.1016/j.bjpt.2025.101226)
Supplement: Supplementary file 1 [file mmc1.pdf]

The Most Effective Therapeutic Exercises for Pain Intensity in Women with Fibromyalgia: A Systematic Review and Network Meta-analysis

**Supplemental Materials**	**Page**
Table S.1. Search strategy	2
Table S.2. Characteristics of included studies	4
Table S.3. Description of CINeMA domain assessments	16
Fig. 1-14. Forest plots for each pairwise comparison	18
Table S.4. Summary of the results obtained	32
Table S.5. Exercise interventions and comparison groups	34
Table S.6. Assessment of local incoherence	35
Table S.7. Summary of results of the pairwise meta-analyses	37
Table S.8. Summary of results of the PEDro scale	38
Fig. 15-28. Funnel plots for each pairwise comparison	40
Table S.9. Judgments for each domain and overall certainty rating for each pairwise comparison (CINeMA assessment)	54

Table S.1. Search strategy

1. MEDLINE

Last searched January 14, 2024

#1 (“Fibromyalgia” OR “Fibromyalgi*” OR “Fibrositis”)

#2 ("Exercise" OR "Resistance Training" OR "Strength Training" OR "Aerobic Training" OR "Flexibility Training" OR "Stretching" OR "Aquatic" OR "Pilates" OR "Zumba" OR "Tai Chi" OR "Qigong" OR "Yoga" OR "Mckenzie" OR "Core Stability" OR "Core training")

#3 ("Pain Measurement" OR "Pain" OR "Pain intensity")

#4 (“Effectiveness” OR “Clinical Trial” OR “Trials” OR “Controlled Trial” OR “Randomized”)

#5 #1 AND #2 AND #3 AND #4

2. CENTRAL by Cochrane Library

Last searched January 14, 2024

#1 (“Fibromyalgia” OR “Fibromyalgi*” OR “Fibrositis”)

#2 ("Exercise" OR "Resistance Training" OR "Strength Training" OR "Aerobic Training" OR "Flexibility Training" OR "Stretching" OR "Aquatic" OR "Pilates" OR "Zumba" OR "Tai Chi" OR "Qigong" OR "Yoga" OR "Mckenzie" OR "Core Stability" OR "Core training")

#3 ("Pain Measurement" OR "Pain" OR “Pain intensity”)

#4 (“Effectiveness” OR “Clinical Trial” OR “Trials” OR “Controlled Trial” OR “Randomized”)

#5 #1 AND #2 AND #3 AND #4

3. Embase

Last searched January 14, 2024

#1 (“Fibromyalgia” OR “Fibromyalgi*” OR “Fibrositis”)

#2 ("Exercise" OR "Resistance Training" OR "Strength Training" OR "Aerobic Training" OR "Flexibility Training" OR "Stretching" OR "Aquatic" OR "Pilates" OR "Zumba" OR "Tai Chi" OR "Qigong" OR "Yoga" OR "Mckenzie" OR "Core Stability" OR "Core training")

#3 ("Pain Measurement" OR "Pain" OR "Pain intensity")

#4 (“Effectiveness” OR “Clinical Trial” OR “Trials” OR “Controlled Trial” OR “Randomized”)

#5 #1 AND #2 AND #3 AND #4

4. Web of Science

Last searched January 14, 2024

#1 (“Fibromyalgia” OR “Fibromyalgi*” OR “Fibrositis”)

#2 All Fields = "Exercise" OR "Resistance Training" OR "Strength Training" OR "Aerobic Training" OR "Flexibility Training" OR "Stretching" OR "Aquatic" OR "Pilates" OR "Zumba" OR "Tai Chi" OR "Qigong" OR "Yoga" OR "Mckenzie" OR "Core Stability" OR "Core training"

#3 All Fields = "Pain Measurement" OR "Pain" OR “Pain intensity”

#4 All Fields = (“Effectiveness” OR “Clinical Trial” OR “Trials” OR “Controlled Trial” OR “Randomized”)

#5 #1 AND #2 AND #3 AND #4

5. Scopus

Last searched January 14, 2024

#1 Search within: Article title, Abstract, Keywords = “Fibromyalgia” OR “Fibromyalgi*” OR “Fibrositis”

#2 Search within: Article title, Abstract, Keywords = "Exercise" OR "Resistance Training" OR "Strength Training" OR "Aerobic Training" OR "Flexibility Training" OR "Stretching" OR "Aquatic" OR "Pilates" OR "Zumba" OR "Tai Chi" OR "Qigong" OR "Yoga" OR "Mckenzie" OR "Core Stability" OR "Core training"

#3 Search within: Article title, Abstract, Keywords = "Pain Measurement" OR "Pain" OR “Pain intensity”

#4 Search within: “Effectiveness” OR “Clinical Trial” OR “Trials” OR “Controlled Trial” OR “Randomized”

#5 #1 AND #2 AND #3 AND #4

6. Cumulative Index to Nursing and Allied Health Literature (CINAHL)

Last searched January 14, 2024

#1 (“Fibromyalgia” OR “Fibromyalgi*” OR “Fibrositis”)

#2 ("Exercise" OR "Resistance Training" OR "Strength Training" OR "Aerobic Training" OR "Flexibility Training" OR "Stretching" OR "Aquatic" OR "Pilates" OR "Zumba" OR "Tai Chi" OR "Qigong" OR "Yoga" OR "Mckenzie" OR "Core Stability" OR "Core training")

#3 ("Pain Measurement" OR "Pain" OR “Pain intensity”)

#4 (“Effectiveness” OR “Clinical Trial” OR “Trials” OR “Controlled Trial” OR “Randomized”)

#5 #1 AND #2 AND #3 AND #4

7. ClinicalTrials.gov

Last searched January 14, 2024

#1 Condition or disease= “Fibromyalgia” OR “Fibromyalgi*” OR “Fibrositis”

#2 Other terms= "Pain Measurement" OR "Pain" OR "Pain intensity"

#3 Intervention or treatment= "Exercise" OR "Resistance Training" OR "Strength Training" OR "Aerobic Training" OR "Flexibility Training" OR "Stretching" OR "Aquatic" OR "Pilates" OR "Zumba" OR "Tai Chi" OR "Qigong" OR "Yoga" OR "Mckenzie" OR "Core Stability" OR "Core training"

#4 Study Status: “All studies”

#5 #1 AND #2 AND #3 AND #4

Table S.2. Characteristics of included studies

Study	Sample	PEDro Score - ACR criteria	Intervention and Follow-up	Reported Results
Mengshoel 1992[42]	n = 25<br>Country: Australia<br>Mean age:<br>32 ± 5 years<br>Dance group:<br>11 – 11<br>Usual care group:<br>14 – 14	7/11<br>-<br>1990	**Dance group**: aerobic dance program.<br>*Intensity:* HR not exceeding 150 beats/min.<br>*Frequency:* 2 d/week (60 min) for 12 weeks.<br>**Usual care group:** control group.<br><br>Follow-up at 0, 10, and 20 weeks.	No statistically significant changes or differences in pain intensity.
Hakkinen 2001[43]	n = 21 Country: Finland<br>Mean age:<br>38 ± 5 years<br>Resistance training group:<br>11 – 11<br>Usual care group:<br>10 – 10	7/11<br>-<br>1990	**Resistance training group:** squat exercise, knee and trunk extension/flexion exercises, and bench press*<br>*Intensity:* initially, 1x15-20 reps (40-60% 1RM), and then 15-20 (70-80% 1RM). Added EES last weeks (40-60% 1RM).<br>*Frequency:* 2 d/week for 21 weeks.<br>**Usual care group:** control group.<br><br>Follow-up at 0 and 21 weeks	Pain intensity improved significantly in resistance training group.
Jentoft 2001[44]	n = 44<br>Country: Norway<br>Mean age:<br>41 ± 10 years<br>Mixed exercise group:<br>22 – 16<br>Aquatic exercise group:<br>22 – 18	5/11<br>-<br>1990	**Mixed exercise group**: Modified version of the Norwegian Aerobic Fitness Model (aerobic dance, flexibility and resistance exercises)*<br>*Intensity:* not specified.<br>*Duration:* 2 d/week (60 min) for 20 weeks.<br>**Aquatic exercise group:** the same program but was realized in pool.<br><br>Follow-up at 0, 20, and 46 weeks.	All variables improved in both groups, but there was no significant difference.
Jones 2002[45]	n = 68<br>Country: United States<br>Mean age:<br>48 ± 10 years<br>Resistance training group:<br>34 - 28<br>Flexibility group:<br>34 - 28	7/11<br>-<br>1990	**Resistance training group:** the main muscle groups were worked (not specified)*<br>*Intensity:* initially 1x4-5 reps, and then to 12.<br>*Duration:* 2 d/week for 12 weeks.<br>**Flexibility group:** the main muscle groups were worked (not specified).<br>*Duration:* 2 d/week for 12 weeks.<br><br>Follow-up at 0 and 12 weeks	Pain improved significantly in resistance training group.
Santen 2002[46]	n = 37<br>Country: Netherlands<br>Mean age:<br>42 ± 17 years<br>Aerobic exercise group:<br>19 – 17<br>Mixed exercise group:<br>18 – 13	7/11<br>-<br>1990	**Aerobic exercise group:** high intensity training (bicycling)*<br>*Intensity:* ≥20-30 min (70% HRmax).<br>*Duration:* 3 d/w (60 min) for 20 weeks.<br>**Mixed exercise group:** low intensity training. Intensive aerobic exercise with flexibility and balance exercise (30 min), and resistance training (10 min)*<br>*Duration:* 2-3 d/w (40-60 min) for 20 weeks.<br><br>Follow-up at 0 and 20 weeks	Pain improved significantly in aerobic exercise group.
Schachter 2003[47]	n = 143<br>Country: Canada	9/11<br>-	**Aerobic exercise (long) group:** long aerobic program*	No statistically significant changes

	Mean age:<br>42 ± 4 years<br>Aerobic exercise (long) group:<br>51 - 29<br>Aerobic exercise (short) group:<br>56 - 26<br>Usual care group:<br>36 - 31	1990	*Intensity:* initially 10 min (40-60% HRR), and then to 30 min (65-75% HRR).<br>*Duration:* 3-5 d/w (1/d) for 16 weeks.<br>**Aerobic exercise (short) group:** short aerobic program*<br>*Intensity:* initially 5 min (40-60 HHR), and then to 15 min (65-75% HHR).<br>*Duration:* 3-5 d/w (2/d) for 16 weeks.<br>**Usual care group:** control group.<br><br>Follow-up at 0, 8, and 16 weeks.	or differences in pain intensity.
Valim 2003[48]	n = 66<br>Country: Brazil<br>Mean age:<br>46 ± 5 years<br>Aerobic exercise group:<br>33 – 32<br>Flexibility group:<br>33 – 28	7/11<br>-<br>1990	**Aerobic exercise group:** supervised walking program.<br>*Intensity:* determined by HR.<br>*Duration:* 3 d/w (45 min) for 20 weeks.<br>**Flexibility group:** 17 exercises including cervical, trunk, and extremities.<br>*Intensity:* maximum position (sustained 30 s) without increased HR.<br>*Duration:* 3 d/w for 20 weeks.<br><br>Follow-up at 0, 10, and 20 weeks.	Pain intensity improved in both groups, but only aerobic exercise group was significant.
Sencan 2004[49]	n = 60<br>Country: Turkey<br>Mean age:<br>35 ± 7 years<br>Aerobic exercise group:<br>20 – 20<br>Drugs therapy group:<br>20 – 20<br>Sham group:<br>20 – 20	7/11<br>-<br>1990	**Aerobic exercise group:** not specified*<br>*Intensity:* not specified.<br>*Duration:* 3 d/w (40 min) for 6 weeks.<br>**Drugs therapy group:** paroxetine (20 mg/d)<br>*Duration:* 7 d/w for 6 weeks.<br>**Sham group:** TENS (placebo).<br>*Duration:* 3 d/w (20 min) for 6 weeks.<br><br>Follow-up at 0, 6, and 24 week (6 months).	Pain intensity improved significantly in both interventions groups (aerobic exercise and drugs therapy).
Valkeinen 2004[50]	n = 26<br>Country: Finland<br>Mean age:<br>61 ± 9 years<br>Resistance training group:<br>13 – 13<br>Usual care group:<br>13 – 13	6/11<br>-<br>1990	**Resistance training group:** the main muscle groups were worked (not specified).<br>*Intensity:* initially 3 sets of 15–20, and then to 4 sets of 8–12 and 5 sets of 5–10.<br>*Duration:* 2 d/w for 21 weeks.<br>**Usual care group:** control group.<br><br>Follow-up at 0 and 21 weeks.	Pain intensity improved in resistance training group, but not significantly.
Gusi 2006[51]	n = 35<br>Country: Spain<br>Mean age:<br>51 ± 1 years<br>Aquatic exercise group:<br>18-17<br>Usual care group:<br>17-17	6/11<br>-<br>1990	**Aquatic exercise group:** aerobic and resistance exercises (knee flexion and extension using water resistance) in water*<br>*Intensity*: aerobic (10 min at 65-75% HRmax) and resistance exercises (4x10 reps).<br>*Duration:* 3 d/w (60 min) for 12 weeks.<br>**Control group:** usual care.<br><br>Follow-up at 0, 12, and 24 weeks.	Pain intensity improved significantly in aquatic exercise group.
Munguia-Izquierdo 2007[52]	n = 60<br>Country: Spain<br>Mean age:<br>48 ± 15 years	4/11<br>-<br>1990	**Aquatic exercise group:** resistance (10-20 min) and aerobic exercise (20-30 min) in water*<br>*Intensity:* aerobic exercise progressed to 50-80% HRmax.<br>*Duration:* 3 d/w for 16 weeks.<br>**Usual care group:** control group.	Pain intensity improved significantly in aquatic exercise group

	Aquatic exercise group:<br>35-29<br>Usual care group:<br>25-24		Follow-up at 0 and 16 weeks.	
Rooks 2007[53]	n = 102<br>Country: United States<br>Mean age:<br>50 ± 7 years<br>Mixed exercise group (1):<br>51 – 35<br>Mixed exercise group (2):<br>51 – 35<br>Education group:<br>50 – 27<br>Combined therapy group:<br>55 – 38	6/11<br>-<br>1990	**Mixed exercise group (1):** treadmill walking (20 min), resistance exercises (25 min: combined machines, hand weights, and calisthenics) and flexibility (primary body movements)*<br>*Intensity:* aerobic (initially 5 min and increased a maximum of 2-4 min/weekly) and resistance exercises (initially 1x6 reps, and then 2x10-12 reps).<br>*Duration:* 2 d/w for 16 weeks.<br>**Mixed exercise group (2):** walking (45 min) and flexibility*<br>**Combined therapy group:** combined therapy and education interventions.<br>*Duration:* 2 d/w for 16 weeks.<br>**Education group:** principles of self-management and supplementary readings (5-15 min).<br>*Duration:* 120 min/2 w for 16 weeks (8 sessions).<br><br>Follow-up at 0 and 16 weeks.	Pain intensity improved significantly in all groups, except education group.
Tomás-Carús 2007[54]	n = 34<br>Country: Spain<br>Mean age:<br>51 ± 1 years<br>Aquatic exercise group:<br>17 – 17<br>Usual care group:<br>17 – 17	7/11<br>-<br>1990	**Aquatic exercise group:** aerobic and resistance exercise (knee flexion and extension), and another aerobic exercise*<br>*Intensity:* aerobic (10 min at 60-65% HRmax) and resistance exercises (20 min: 4x10 reps)<br>*Duration:* 3 d/w (60 min) for 12 weeks.<br>**Usual care group:** control group.<br><br>Follow-up at 0, 12, and 24 weeks.	Pain intensity improved significantly in aquatic exercise group.
Alentorn-Geli 2008[55]	n = 24<br>Country: Spain<br>Mean age:<br>56 ± 9 years<br>Vibration exercise group<br>12 – 11<br>Usual care group<br>12 – 10	7/11<br>-<br>1990	**Vibration exercise group**: mixed exercise + protocol WBV (6 isometric exercises of lower extremities).<br>*Intensity:* 6x30 sec/exercise (3 min rest between rep). WBV: 30 Hz (F) and 2 mm (A). Initially, duration of WBV was 4-5 min/session, and then 18 min/session.<br>*Duration:* 2 d/w (90 min) for 6 weeks.<br>**Usual care group:** control group.<br><br>Follow-up at 0 and 6 weeks.	Pain intensity improved significantly in vibration exercise group.
Bircan 2008[56]	n = 30<br>Country: Turkey<br>Mean age:<br>47 ± 6 years<br>Aerobic exercise group:<br>15 – 13<br>Resistance training group:<br>15 – 13	7/11<br>-<br>1990	**Aerobic exercise group:** treadmill walking<br>*Intensity:* initially 20 min, and then 30 min (60-70% HRmax).<br>*Duration:* 2 d/w for 8 weeks.<br>**Resistance training group:** free and body weight were used*<br>*Intensity:* initially 1x4 reps and then to 12 reps.<br>*Duration:* 2 d/w for 8 weeks.<br><br>Follow-up at 0 and 8 weeks.	Pain intensity improved significantly in both groups.
Bresan 2008[57]	n = 15<br>Country: Brazil	5/11<br>-	**Aerobic exercise group:** treadmill walking*<br>*Intensity:* 25 min at 60-65% HRmax.	Pain intensity improved in both

	Mean age:<br>47 ± 10 years<br>Aerobic exercise group:<br>7 – 7<br>Flexibility group:<br>8 – 8	1990	*Duration:* 1 d/w (30 min) for 8 weeks.<br>**Flexibility group:** sural triceps, hamstrings, glutes, paravertebral muscles, latissimus dorsi, pectorals, trapezius, and respiratory muscles stretches.<br>*Intensity:* 5x30 sec<br>*Duration:* 1 d/w (40-45 min) for 8 weeks.<br><br>Follow-up at 0 and 8 weeks.	groups, but only flexibility group was significant.
Evcik 2008[58]	n = 63<br>Country: Turkey<br>Mean age:<br>43 ± 4 years<br>Aquatic exercise group:<br>33 – 31<br>Mixed exercise group:<br>30 – 30	5/11<br>-<br>1990	**Aquatic exercise group:** aerobic (jumping and jogging), mobility and flexibility exercises in water*<br>*Duration:* 3 d/w (60 min) for 5 weeks.<br>**Mixed group:** home-based exercise program*<br>*Duration:* 3 d/w (60 min) for 5 weeks.<br><br>Follow-up at 0, 4, 12, and 24 weeks.	Pain intensity improved significantly, but no significant difference was found between the groups.
Ide 2008[59]	n = 40<br>Country: Brazil<br>Mean age:<br>46 ± 4 years<br>Aquatic exercise group:<br>20 – 15<br>Usual care group:<br>20 – 11	7/11<br>-<br>1990	**Aquatic exercise group:** aquatic respiratory exercise program (45 min)*<br>*Duration:* 4 d/w for 4 weeks.<br>**Usual care group:** control group.<br>*Duration:* four times a week for 4 weeks.<br><br>Follow-up at 0 and 4 weeks.	Pain intensity improved significantly in aquatic exercise group.
Tomas-Carus2008[60]	n = 33<br>Country: Spain<br>Mean age:<br>51 ± 1 years<br>Aquatic exercise group:<br>17 – 15<br>Usual care group:<br>16 – 15	8/11<br>-<br>1990	**Aquatic exercise group:** aerobic and resistance exercises (knee flexion and extension), and another aerobic exercise*<br>*Intensity:* aerobic (10 min at 65-75% HRmax) and resistance exercises (20 min: 4x10 reps)<br>*Duration:* 3 d/w (60 min) for 8 months.<br>**Usual care group:** control group.<br><br>Follow-up at 0 and 8 months.	Pain intensity improved significantly in aquatic exercise group.
Valkeinen 2008[61]	n = 25<br>Country: Finland<br>Mean age:<br>59 ± 3 years<br>Mixed exercise group:<br>15 – 13<br>Usual care group:<br>11 – 11	6/11<br>-<br>1990	**Mixed exercise group:** resistance (7-8 exercises for main muscle groups) and aerobic exercises (60 min: walking or cycling).<br>*Intensity:* Odd weeks 2 resistance training sessions and 1 aerobic exercise session, and even weeks vice-versa. On average, 1.5 resistance training and 1.5 aerobic exercise were performed each week.<br>*Duration:* 3 d/w for 21 weeks.<br>**Usual care group:** control group.<br><br>Follow-up at 0, 7, 14, and 21 weeks.	Pain intensity improved significantly in mixed exercise group.
Altan 2009[62]	n = 50<br>Country: Turkey<br>Mean age:<br>49 ± 6 years<br>Pilates group:<br>25 – 25<br>Flexibility group:<br>25 – 24	8/11<br>-<br>1990	**Pilates group:** based in basic principles of the Pilates method.<br>*Intensity:* resistance bands and 26cm Pilates balls were used.<br>*Duration:* 3 d/w for 12 weeks.<br>**Flexibility group:** passive and active stretching.<br>*Intensity:* not specified.<br>*Duration:* 3 d/w for 12 weeks.<br><br>Follow-up at 0, 12, and 24 weeks.	Pain intensity improved significantly in Pilates group at 12 weeks.

Carson 2010[63]	n = 53<br>Country: United States<br>Mean age:<br>53 ± 12 years*<br>Yoga group:<br>25 – 22<br>Usual care group:<br>28 – 26	8/11<br>-<br>1990	**Yoga group**: Yoga of Awareness program.<br>*Duration:* 1 d/w (120 min) for 8 weeks. Patients were encouraged to practice at home 20-40 min/d every day (guided by DVD)<br>**Usual care group:** control group.<br>Follow-up at 0 and 8 weeks.	Pain intensity improved significantly in Yoga group.
Kayo 2011[64]	n = 90<br>Country: Brazil<br>Mean age:<br>47 ± 4 years<br>Aerobic exercise group<br>30 – 23<br>Resistance training group<br>30 – 22<br>Control group<br>30 – 23	6/11<br>-<br>1990	**Aerobic exercise group:** walking*<br>*Intensity:* initially 25-30 min, and then 50 min (40-50% HHR, progressed to 60-70%)<br>**Resistance training group:** 11 free active exercises, using free and body weight.<br>*Intensity:* initially 3x10 reps, and then 3x15 (rest of 1 min between sets).<br>*Duration:* 3 d/w for 16 weeks.<br>**Usual care group:** control group.<br><br>Follow-up at 0, 8, 16, and 28 weeks (seven months)	Pain intensity improved significantly in aerobic exercise group.
Baptista 2012[65]	n = 80<br>Country: Brazil<br>Mean age:<br>49 ± 2 years<br>Dance group:<br>40 – 38<br>Usual care group:<br>40 – 37	8/11<br>-<br>1990	**Dance group:** aerobic dance program*<br>*Duration:* 2 d/w for 16 weeks.<br>**Usual care group:** control group.<br><br>Follow-up at 0, 16, and 32 weeks.	Pain intensity improved significantly in dance group.
López-Rodríguez 2012[66]	n = 70<br>Country: Spain<br>Mean age:<br>55 ± 1 years<br>Aquatic exercise group:<br>35 – 19<br>Flexibility group:<br>35 – 20	5/11<br>-<br>2010	**Aquatic exercise group:** walking and slow movements*<br>*Intensity:* 40 min (not specified).<br>*Duration:* 2 d/w (60 min) for 12 weeks.<br>**Flexibility group:** global stretching.<br>*Duration:* 2 d/w (60 min) for 12 weeks.<br><br>Follow-up at 0 and 12 weeks.	Pain intensity improved significantly in the aquatic exercise group. There was no difference in the flexibility group.
Letieri 2013[67]	n = 66<br>Country: Brazil<br>Mean age:<br>59 ± 6 years<br>Aquatic exercise group: 33 – 33<br>Usual care group:<br>33 – 31	5/11<br>-<br>1990	**Aquatic exercise group:** resistance, mobility, and balance exercises*<br>*Intensity:* 35 min (not specified).<br>*Duration:* 2 d/w for 15 weeks.<br>**Usual care group:** control group.<br><br>Follow-up at 0 and 15 weeks.	Pain intensity improved significantly in aquatic exercise group.
López-Rodríguez 2013[68]	n = 76<br>Country: Spain<br>Mean age:<br>55 ± 5 years<br>Aquatic exercise group:<br>38 – 30<br>Flexibility group:<br>38 – 29	8/11<br>-<br>2010	**Aquatic exercise group:** aquatic dance program (aquatic biodance)*<br>*Intensity:* 40 min (not specified).<br>*Duration:* 2 d/w for 12 weeks.<br>**Flexibility group:** stretching of main muscle groups.<br>*Duration:* 2 d/w for 12 weeks.	Pain intensity improved significantly in aquatic exercise group. There was no difference in flexibility group.

Duruturk 2014[69]	n = 33<br>Country: Turkey<br>Mean age:<br>51 ± 15 years<br>Aerobic exercise group:<br>17 – 14<br>Balance exercise group:<br>16 – 12	6/11<br>-<br>1990	**Aerobic exercise group:** treadmill walking*<br>*Intensity:* 20-45 min at 60-75% HRmax. Increased according to patient´s tolerance.<br>*Duration:* 3 d/w for 6 weeks.<br>**Balance exercise group:** postural biofeedback exercises (catch, speedball, sky ball, and gotcha)*<br>*Duration:* 3 d/w (20-30 min) for 6 weeks.<br><br>Follow-up at 0 and 6 weeks.	Pain intensity improved significantly in both groups, but aerobic exercise group was higher than balance exercise group.
Gavi 2014[70]	n = 80<br>Country: Brazil<br>Mean age:<br>46 ± 18 years<br>Resistance training group:<br>40 – 35<br>Flexibility group:<br>40 – 31	7/11<br>-<br>1990	**Resistance training group:** leg press, leg extension, hip flexion, fly, triceps extension, shoulder flexion, abduction and extension, leg curl, calf, pulldown, biceps flexion.<br>*Intensity:* 3x12 reps (45% 1RM)<br>*Duration:* 2 d/w for 16 weeks.<br>**Flexibility group:** the main muscle groups were worked (not specified).<br>*Duration:* 2 d/w for 16 weeks.<br><br>Follow-up at 0 and 16 weeks.	Pain intensity improved significantly in both groups. There was no significant difference between groups.
Genc 2015[71]	n = 54<br>Country: Turkey<br>Mean age:<br>36 ± 7 years<br>Flexibility group:<br>27 – 25<br>Mixed exercise group:<br>27 – 25	6/11<br>-<br>1990	**Flexibility group:** the main muscle groups were worked<br>*Duration:* 2 d/w for 6 weeks.<br>**Mixed exercise group:** the same flexibility program with aerobic exercise (treadmill walking).<br>*Intensity:* aerobic exercise (40-50 min at 60-75% HRmax)<br>*Duration:* 3 d/w for 6 weeks.<br><br>Follow-up at 0 and 6 weeks.	Pain intensity improved significantly in both groups.
Larsson 2015[72]	n = 130<br>Country: Sweden<br>Mean age:<br>51 ± 7 years<br>Resistance training group:<br>67 – 48<br>Relaxation group:<br>63 – 43	6/11<br>-<br>1990	**Resistance training group:** leg-press, knee extension and flexion, biceps curl and hand grip strength, heel raise and core stability*<br>*Intensity:* initially 1-2x10-12 reps (40-60% 1RM), and then 1-2x5-8 reps (80% 1RM)<br>50 min. Added EES last weeks. 1 min rest between sets.<br>*Duration:* 2 d/w (50 min) for 15 weeks.<br>**Relaxation group:** relaxation therapy.<br>*Duration:* 2 d/w (25 min) for 15 weeks.<br><br>Follow-up at 0 and 15 weeks, and 13 and 18 months.	Pain intensity improved significantly in resistance training group.
Latorre 2015[73]	n = 39<br>Country: Spain<br>Mean age:<br>51 ± 5 years<br>Functional training group:<br>20 – 20<br>Usual care group:<br>19 – 19	7/11<br>-<br>1990	**Functional training group**: circuit of resistance and balance exercises (in pool and on land)*<br>*Intensity:* 1-3x8-12 reps with 0.5-2 kg (according to patient´s tolerance).<br>*Duration:* 3 d/w (50 min) for 18 weeks.<br>**Usual care group:** control group.<br>**Duration:** three times a week for 18 weeks.<br><br>Follow-up at 0 and 18 weeks.	Pain intensity improved significantly in functional training group.
Sañudo 2015[74]	n = 32<br>Country: Spain<br>Mean age:<br>57 ± 8 years<br>Aerobic exercise group:	7/11<br>-<br>2016	**Aerobic exercise group:** continuous walking with arm movements and jogging and interval training (6 reps of 1.5 min with 1 min rest between them)*<br>*Intensity:* continuous (15-20 min at 60-65% HRmax) and interval training (15 min at 75-80% HRmax)<br>*Duration:* 2 d/w for 24 weeks.	Pain intensity improved in aerobic exercise group, but not significantly.

	16 – 16<br>Usual care group:<br>16 – 12		**Usual care group:** control group.<br><br>Follow-up at 0 and 24 weeks.	
Sevimli 2015[75]	n = 75<br>Country: Turkey<br>Mean age:<br>35 ± 9 years*<br>Aerobic exercise group:<br>25 – 25<br>Aquatic exercise group: 25 – 25<br>Mixed exercise group:<br>25 – 25	7/11<br>-<br>1990	**Aerobic exercise group:** gymnastic-based aerobic exercise program.<br>*Intensity:* Initially 3x3 min with 30 sec rest between them (60-80% HRmax), and then 4x30 min (30 sec rest) and 5x30 min (10 sec rest).<br>*Duration:* 2 d/w (40-50 min) for 12 weeks.<br>**Aquatic exercise group:** aquatic aerobic program.<br>*Duration:* 2 d/w (40-50 min) for 12 weeks.<br>**Mixed exercise group:** isometric and flexibility exercises.<br>*Duration:* 15 min/d for 12 weeks.<br><br>Follow-up at 0 and 12 weeks.	Pain intensity improved significantly in aquatic and aerobic exercise groups.
Ekici 2016[76]	n = 74<br>Country: Turkey<br>Mean age:<br>37 ± 1 years<br>Pilates group:<br>21 – 15<br>Manual therapy group:<br>22 – 21	7/11<br>-<br>1990	**Pilates group:** based in basic principles of the Pilates method.<br>*Intensity:* increased gradually (from 5 reps to 10 reps)<br>*Duration:* 3 d/w (60 min) for 4 weeks.<br>**Manual therapy group:** connective tissue massage<br>*Duration:* 3 d/w (5-20 min) for 4 weeks.<br><br>Follow-up at 0 and 4 weeks.	Pain intensity improved significantly in both groups.
Fernandes 2016[77]	n = 75<br>Country: Brazil<br>Mean age:<br>49 ± 4 years<br>Aerobic exercise group:<br>36 – 34<br>Aquatic exercise group: 39 – 37	9/11<br>-[77]<br>1990	**Aerobic exercise group:** walking*<br>*Intensity:* 40 min (not specified).<br>*Duration:* 3 d/w (50 min) for 12 weeks.<br>**Aquatic exercise group:** freestyle swimming<br>*Intensity:* 40 min (not specified).<br>*Duration:* 3 d/w (50 min) for 12 weeks.<br><br>Follow-up at 0, 6 and 12 weeks.	Pain intensity improved significantly in both groups.
Alev 2017[78]	n = 20<br>Country: Turkey<br>Mean age:<br>57 ± 4 years<br>Vibration exercise group:<br>10 – 10<br>Combined therapy group:<br>10 – 10	9/11<br>-<br>2010	**Vibration exercise group:** resistance exercises (6 isometric and isotonic exercises) with WBV.<br>*Intensity:* 6x30 sec (3 min rest between then). WBV: 30 Hz (F) and 2 mm (A).<br>*Duration:* 2 d/w for 4 weeks.<br>**Combined therapy group:** the same program, but without vibratory stimulus (placebo).<br><br>Follow-up at 0, 12, and 24 weeks.	Pain intensity improved in both groups, but not significantly.
Assumpção 2017[79]	n = 53<br>Country: Brazil<br>Mean age:<br>47 ± 4 years<br>Resistance training group:<br>19 – 16<br>Flexibility group:<br>18 – 14<br>Usual care group:	6/11<br>-<br>1990	**Resistance training group:** the main muscle groups were worked.<br>*Intensity:* 1x8 reps. Initially no load was used, and then 0.5 kg was added each week (increased according to patient´s tolerance).<br>*Duration:* 2 d/w for 12 weeks.<br>**Flexibility group:** postural reeducation method.<br>*Intensity:* 3x30 sec (increased according to patient´s tolerance)<br>*Duration:* 2 d/w for 12 weeks.<br>**Usual care group:** control group.	Pain intensity improved in both intervention groups.

	16 – 14		Follow-up at 0 and 12 weeks.	
Collado-Mateo 2017[80]	n = 83<br>Country: Spain<br>Mean age:<br>52 ± 1 years<br>Virtual reality group<br>42 – 41<br>Usual care group<br>41 – 35	7/11<br>-<br>1990/ 2010	**Virtual reality group**: exargame (VirtualEx-FM) focused on postural control and coordination.<br>*Intensity:* no specified.<br>*Duration:* 2 d/w for 8 weeks.<br>**Usual care:** control group.<br><br>Follow-up at 0 and 8 weeks.	Pain intensity improved significantly.
Toprak 2017[81]	n = 49<br>Country: Turkey<br>Mean age:<br>42 ± 7 years<br>Mixed exercise group:<br>24 – 20<br>Combined therapy group:<br>25 – 20	8/11<br>-<br>1990	**Mixed exercise group:** aerobic (treadmill walking), resistance (10-12 exercises with elastic resistive bands) and flexibility exercises*<br>*Intensity:* aerobic (20 min at 65-70% HRmax, progressed 75-80%), resistance (1x15 reps and progressed according to patient´s tolerance) and flexibility exercises (10x10 s)<br>*Duration:* 2 d/w (50 min) for 6 weeks.<br>**Combined therapy group:** the same program, but manual therapy was included.<br>*Duration:* 2 d/w (5-20 min) for 6 weeks.<br><br>Follow-up at 0 and 6 weeks.	Pain intensity improved significantly in both groups.
Acosta-Gallego 2018[82]	n = 90<br>Country: Spain<br>Mean age:<br>58 ± 1 years<br>Aquatic exercise group: 45 – 36<br>Aerobic exercise group:<br>45 – 37	6/11<br>-<br>1990	**Aquatic exercise group:** aquatic aerobic program (standard physical rehabilitation intervention program in pool)*<br>*Intensity:* not specified.<br>*Duration:* 2 d/w (50 min) 20 weeks.<br>**Aerobic exercise group:** the same program land-based.<br><br>Follow-up at 0 and 20 weeks.	Pain intensity improved in aquatic exercise group
Ernberg 2018[83]	n = 125<br>Country: Sweden<br>Mean age:<br>51 ± 9 years<br>Resistance training group:<br>67 – 49<br>Relaxation group:<br>58 – 43	6/11<br>-<br>1990	**Resistance training group:** leg-press, knee extension and flexion, biceps curl and hand grip strength, heel raise and core stability*<br>*Intensity:* initially 1-2x10-12 reps (40-60% 1RM), and then 1-2x5-8 reps (80% 1RM)<br>50 min. Added EES last weeks. 1 min rest between sets.<br>*Duration:* 2 d/w (50 min) for 15 weeks.<br>**RT group:** relaxation therapy.<br>*Duration:* 2 d/w (25 min) for 15 weeks.<br><br>Follow-up at 0 and 15 weeks.	Pain intensity improved significantly in resistance training group.
Wong 2018[84]	n = 37<br>Country: Korea<br>Mean age:<br>51 ± 1 years<br>Tai Chi group:<br>18 – 17<br>Usual care group:<br>19 – 14	8/11<br>-<br>1990	**Tai Chi group**: exercise program based on the principles of Tai Chi.<br>*Intensity:* 40-50% HRR<br>*Duration:* 3 d/w (55 min) for 12 weeks.<br>**Usual care group:** control group.<br><br>Follow-up at 0 and 12 weeks.	Pain intensity improved significantly in Tai Chi group.
Andrade 2019[85]	n = 54<br>Country: Brazil<br>Mean age:<br>48 ± 4 years	8/11<br>-<br>1990	**Aquatic exercise group:** aerobic (walking), flexibility and resistance exercises*<br>*Intensity:* initially 80% VAT HR (sitting on floats), and then 110% VAT HR (jumping) and 100% VAT HR (aquatic cycle).<br>*Duration:* 2 d/w for 16 weeks.	Pain intensity improved significantly in aquatic exercise group.

	Aquatic exercise group: 27 – 22<br>Usual care group: 27–24		**Usual care group:** control group.<br><br>Follow-up at 0, 16, and 32 weeks.	
Gómez-Hernández 2019[86]	n = 64<br>Country: Spain<br>Mean age:<br>54 ± 7 years*<br>Aerobic exercise group:<br>32 – 32<br>Mixed exercise group:<br>32 – 32	9/11<br>-<br>1990	**Aerobic exercise group:** supervised stationary cycling program*****<br>*Intensity:* 10 min at 50-70% HRmax<br>*Duration:* 3 d/w for 12 weeks.<br>**Mixed exercise group:** the same aerobic program with flexibility exercises.<br>*Duration:* (flexibility exercises) 1 d/w (45 min) for 12 weeks.<br><br>Follow-up at 0, 4 and 12 weeks.	Pain intensity improved significantly in both groups.
Jablochkova 2019[87]	n = 75<br>Country: Sweden<br>Mean age:<br>51 ± 10 years<br>Resistance training group:<br>41 – 38<br>Relaxation group:<br>34 – 34	6/11<br>-<br>1990	**Resistance training group:** leg-press, knee extension and flexion, biceps curl and hand grip strength, heel raise and core stability*<br>*Intensity:* initially 1-2x10-12 reps (40-60% 1RM), and then 1-2x5-8 reps (80% 1RM)<br>50 min. Added EES last weeks. 1 min rest between sets.<br>*Duration:* 2 d/w (50 min) for 15 weeks.<br>**Relaxation group:** relaxation therapy contained mental exercises.<br>*Duration:* 2 d/w (25 min) for 15 weeks.<br><br>Follow-up at 0 and 15 weeks.	Pain intensity improved significantly in resistance training group.
Silva 2019[88]	n = 60<br>Country: Brazil<br>Mean age:<br>53 ± 1 years<br>Resistance training group:<br>30 – 28<br>Relaxation group:<br>30 – 27	8/11<br>-<br>1990/ 2010	**Resistance training group:** biceps brachial, triceps, pectoralis, trapezius, knee extensors and flexors, and hip abductors.<br>*Intensity:* 3x12 rep at 60% 1RM, and then 70-80% 1RM.<br>*Duration:* 2 d/w for 12 weeks.<br>**Relaxation group:** sophrology technique (combined breathing and relaxation exercises, body awareness, visualization, gentle movement and meditation).<br>*Duration:* 2 d/w (40 min) for 12 weeks.<br><br>Follow-up at 0, 4, 8, and 12 weeks.	Pain intensity improved significantly in both groups.
Toprak 2019[89]	n = 42<br>Country: Turkey<br>Mean age:<br>49 ± 1 years<br>Core stability group:<br>21 – 19<br>Combined therapy group:<br>21 – 17	8/11<br>-<br>1990	**Core stability group**: focused on abdominal muscle activity (isometric and dynamic exercises)*<br>*Intensity:* isometric (10x10 s), dynamic exercises (6-12 reps with elastic resistive bands).<br>*Duration:* 2 d/w (40 min) for 6 weeks.<br>**Combined therapy group:** the same program but therapeutic tape was applied.<br><br>Follow-up at 0 and 6 weeks.	Pain intensity improved significantly in both groups.
Villafaina 2019[90]	n = 55<br>Country: Spain<br>Mean age:<br>53 ± 6 years<br>Virtual reality group:<br>28 – 25 – 15<br>Usual care group:<br>27 – 25 – 15	7/11<br>-<br>2010	**Virtual reality group**: exergame (VirtualEx-FM) based aerobic dance, walking, postural control and coordination games.<br>*Intensity:* no specified.<br>*Duration:* 2 d/w (60 min) for 24weeks.<br>**Usual care group:** control group.<br><br>Follow-up at 0, 24, and 48 weeks.	Pain intensity improved significantly in virtual reality group.
Atan 2020[91]	n = 60<br>Country: Turkey<br>Mean age:	8/11<br>-<br>2016	**Mixed exercise (high) group:** aerobic (HIIT), resistance and flexibility exercises (major muscle groups)*	Pain intensity improved significantly in both intervention groups.

	49 ± 14 years<br>Mixed exercise (high) group<br>20 – 19<br>Mixed exercise (moderate) group<br>20 – 19<br>Usual care group<br>20 – 17		*Intensity:* aerobic exercise (4x4 min at 80-95% HRmax followed by 3 min recovery intervals at 70% HRmax), resistance (1x8-10 reps with 1-3 kg) and flexibility (4-5x20-30 sec).<br>*Duration:* 5 d/w (35 min) for 6 weeks.<br>**Mixed exercise (moderate) group:** aerobic (MICT), resistance and flexibility exercises (major muscle groups)*<br>*Intensity:* aerobic (45 min at 65-70% HRmax), resistance (1x8-10 reps with 1-3 kg) and flexibility exercises (4-5x20-30 sec).<br>*Duration:* 5 d/w (55 min) for 6 weeks<br>**Usual care group:** control group.<br><br>Follow-up at 0 and 6 weeks.	
Britto 2020[92]	n = 33<br>Country: Brazil<br>Mean age:<br>48 ± 12 years<br>Mixed exercise group:<br>17– 17<br>Aquatic exercise group: 16 – 16	9/11<br>-<br>2010	**Mixed exercise group:** resistance and flexibility exercises (posterior and anterior muscle chains)*<br>*Intensity:* resistance (3x15 reps) and flexibility exercises (3x15-25 sec).<br>*Duration:* 3 d/w for 8 weeks.<br>**Aquatic exercise group:** a pool-based aquatic exercise (the same program).<br><br>Follow-up at 0 and 8 weeks.	Pain intensity improved significantly in both groups.
De Medeiros 2020[93]	n = 42<br>Country: Brazil<br>Mean age:<br>48 ± 17 years<br>Aquatic exercise group: 21 – 19<br>Pilates group: 21 – 18	9/11<br>-<br>2010	**Aquatic exercise group:** aquatic aerobic program*<br>*Intensity:* increased the speed of the exercises in each session.<br>*Duration:* 2 d/w (40 min) for 16 weeks.<br>**Pilates group:** Mat Pilates method.<br>*Intensity:* initially 1x8 reps, and then 2x10 reps and 3x8 reps<br>*Duration:* 2 d/w (50 min) for 16 weeks.<br><br>Follow-up at 0 and 12 weeks.	Pain intensity improved significantly in both groups.
Gulsen 2020[94]	n = 20<br>Country: Turkey<br>Mean age:<br>42 ± 10 years<br>Virtual reality group:<br>10-8<br>Mixed exercise group:<br>10-8	7/11<br>-<br>2010	**Virtual reality group**: aerobic exercise and Pilates with immersive virtual reality (20 min).<br>*Intensity:* not specified.<br>*Duration:* 2 d/w for 8 weeks.<br>**Mixed exercise group:** the same program, but without virtual reality treatment.<br><br>Follow-up at 0 and 8 weeks.	Pain intensity improved significantly in both groups.
Sarmento 2020[95]	n = 28<br>Country: United States<br>Mean age:<br>49 ± 30 years<br>Qigong group:<br>14 – 10<br>Sham group:<br>14 – 10	7/11<br>-<br>1990/<br>2010	**Qigong group**: a type of Qigong ("six healing sounds").<br>*Intensity:* not specified.<br>*Duration:* 2 d/w (25 min) for 10 weeks.<br>**Sham group:** sham-Qigong (healing sounds not taught).<br><br>Follow-up at 0 and 10 weeks.	Pain intensity improved significantly in Qigong group.
De Carvalho 2020[96]	n = 35<br>Country: Brazil<br>Mean age:<br>51 ± 23 years<br>Virtual reality group:<br>16 – 11<br>Flexibility group:	7/11<br>-<br>2010	**Virtual reality group:** 6 subgames of Wii Fit Plus.<br>*Duration:* 3 d/w for 7 weeks.<br>**Flexibility group:** chain muscle stretching technique.<br>*Intensity:* positions were held during 4 deep and prolonged expirations.<br>*Duration:* 3 d/w for 7 weeks.<br><br>Follow-up at 0, 4, and 7 weeks.	Pain intensity improved significantly in both groups.

	19 – 10			
Swar 2020[97]	n = 60<br>Country: Egypt<br>Mean age:<br>53 ± 3 years<br>Aquatic exercise group: 30 – 30<br>Mixed exercise group: 30 – 30	7/11<br>-<br>2010	**Aquatic exercise group:** aerobic (bicycling simulation, walk around pool and aerobics dance) and resistance exercises (water resistance).<br>*Intensity:* not specified.<br>*Duration:* 2 d/w for 8 weeks.<br>**Mixed exercise group:** aerobic (walk and aerobics dance), resistance (8-10 exercises) and flexibility exercises.<br>*Intensity:* 1-3x8-12 reps with 0.5-2 kg (loads were increased gradually).<br><br>Follow-up at 0 and 8 weeks.	Pain intensity improved in both groups, but only aquatic exercise group was significant.
Arakaki 2021[98]	n = 60<br>Country: Brazil<br>Mean age:<br>47 ± 1 years<br>Resistance training group:<br>30 – 28<br>Flexibility group:<br>30 – 26	9/11<br>-<br>1990	**Resistance training group:** resistance exercises using a Swiss ball (65 cm-diameter) and dumbbells.<br>*Intensity:* 3x12 reps at 60% 1RM (1-2 min rest between exercise)<br>**Flexibility group:** same muscles trained in the Swiss ball group.<br>*Intensity:* 3x30 s each position.<br>*Duration:* 3 d/w (40 min) for 12 weeks.<br><br>Follow-up at 0, 6, and 12 weeks.	Pain intensity improved significantly in both groups, but higher in resistance training group than flexibility group.
Garrido-Ardila 2021[99]	n = 135<br>Country: Spain<br>Mean age:<br>56 ± 8 years*<br>Core stability group:<br>46 – 36<br>Acupuncture group:<br>45 – 34<br>Usual care group:<br>45 – 33	8/11<br>-<br>1990	**Core stability group:** coordination of breathing and core activation (7 exercises).<br>*Intensity:* not specified.<br>*Duration:* 2 d/w (30 min) for 6 weeks.<br>**Acupuncture group:** acupuncture points from Traditional Chinese Medicine.<br>*Duration:* 2 d/w for 6 weeks.<br>**Usual care group:** control group.<br><br>Follow-up at 0, 6, and 13 weeks.	Pain intensity improved in both intervention groups, but not significantly.
Hernando-Garijo 2021[100]	n = 34<br>Country: Spain<br>Mean age:<br>53± 10 years<br>Aerobic exercise group:<br>17 – 14<br>Usual care group:<br>17 – 14	9/11<br>-<br>1990	**Aerobic exercise group:** telerehabilitation aerobic program.<br>*Intensity:* 10, 15, 20, or 25 reps/min (increased according to patient´s tolerance).<br>*Duration:* 2 d/w (50 min) for 15 weeks.<br>**Usual care group:** control group.<br><br>Follow-up at 0 and 15 weeks.	Pain intensity improved significantly in aerobic exercise group.
Rodríguez-Mansilla 2021[101]	n = 141<br>Country: Spain<br>Mean age:<br>52 ± 6 years*<br>Qigong group:<br>47 – 31<br>Mixed exercise group:<br>47 – 33<br>Usual care group:<br>47 – 29	7/11<br>-<br>2010	**Qigong group:** a type of Qigong ("twenty Wang Ziping figures for health and longevity')<br>*Intensity:* 6 reps each figure.<br>*Duration:* 2 d/w for 4 weeks.<br>**Mixed exercise group:** aerobic, resistance, balance and flexibility exercises.<br>*Duration:* 2 d/w for 4 weeks.<br>**Usual care group:** control group.<br><br>Follow-up at 0 and 4 weeks.	Pain intensity improved significantly in both interventions groups.

* Included 5-10 min of warm-up and cool-down (mobility and relaxation exercises, slow walks, and stretching).

Mean age: mean ± standard deviation (SD). The mean age and SD between groups were calculated from the mean and sample size reported by each study. *Overall mean age and SD of the sample reported by each study. Legend: EES, exercises for explosive strength; FM, fibromyalgia; HIIT, high-intensity interval training; HR, heart rate; HRmax, maximum heart rate; HRR,

heart rate reserve; MICT, moderate-intensity continuous training; PNE, pain neuroscience education; RM, repetition maximum; TENS, transcutaneous electrical nerve stimulation; VAT, ventilatory anaerobic threshold.

Table S.3. Description of CINeMA domain assessments: risk of bias, indirectness, incoherence, heterogeneity, imprecision, publication bias.

CINeMA Domains	Description of assessment
**Risk of bias (ROB)**	Within-study bias domain refers to limitations in the individual studies that may lead to a biased estimated relative treatment effect.<br><br>We used the PEDro scale to assess 11 items (eligibility criteria, random allocation, concealed allocation, baseline comparability, blind subjects, blind therapists, blind assessors, adequate follow-up, intention-to-treat analysis, between-group comparisons, point estimates and variability) as low, unclear, or high ROB for each included study. The included studies were classified according to scores of 9 or 10, 6 to 8, and ≤ 5 on the PEDro scale and were interpreted as excellent (low ROB), good (moderate ROB), and fair quality (high ROB), respectively[62]
**Indirectness**	Indirectness refers to the relevance of the included studies to the research question, which includes the definition of the population, interventions, and outcomes of interest. A core assumption in NMA is that of transitivity; that there is an underlying true relative treatment effect which applies to all studies regardless of the treatments being compared. CINeMA's approach for indirectness intends to also address the assumption of transitivity by indicating which comparisons may suffer from different definitions of the setting of interest. Assuming that transitivity holds implies that consistency—which refers to the agreement of the estimated treatment effects—also holds.<br><br>The population of interest were women with fibromyalgia. All settings, including healthcare, occupational, general population and mixed, were relevant for this systematic review. The intervention of interest in this review was therapeutic exercise treatment. These interventions encompassed a diverse set of treatments characterized by several interacting components and heterogeneous in treatment design and delivery. Pairwise comparison indirectness was computed for the studies contributing to the direct and indirect meta-analyses using the CINeMA web application based on individual study ratings. Transitivity was investigated by considering distribution of potential treatment effect modifiers.
**Incoherence**	Incoherence refers to the range of effects that are considered clinically important<br><br>We assessed incoherence for the assessment of certainty of evidence

	using the SIDE split approach. This approach separates the indirect meta-analysis effect from mixed NMA effect. We assessed incoherence using the approach recommended by CINeMA, which estimates an inconsistency factor with 95% interval (ratio of direct and indirect estimates; no concerns about incoherence if the p-value from SIDE >0.10) and assesses the difference between direct and indirect estimates considering the range of equivalence based on a defined clinically important difference. Because we used a random effects model and standardized mean difference, we assume a moderate to large effect size (SMD =<0.6) as a clinically important difference.
**Heterogeneity**	Heterogeneity domain refers to the certainty with which the variability in the results of studies contributing to each comparison.<br><br>We assessed heterogeneity by comparing the 95% confidence interval of the pairwise NMA estimate with the prediction interval for NMA estimate. We judged heterogeneity following CINeMA recommendations, by assessing how many crossings of the interval with null effect and clinically important value (SMD =<0.6) in opposite direction as point estimate.
**Imprecision**	Imprecision domain refers to the certainty with which each effect is estimated.<br><br>We considered the treatment effects included in the 95% confidence interval relative to potentially clinically important differences to assess the precision of the NMA estimate for each pairwise comparison.
**Publication bias**	Reporting bias results from the inclusion in the systematic review of a nonrepresentative set of the eligible studies, that may occur for example from an uncomplete literature search.

Fig. S.1. Forest plot of the meta-analyses of those studies evaluating the effect of Aquatic Exercise compared to any treatment in relation to pain intensity using Visual Analogue Scale. Short term (≤ 3 months). Long term (> 3 months).

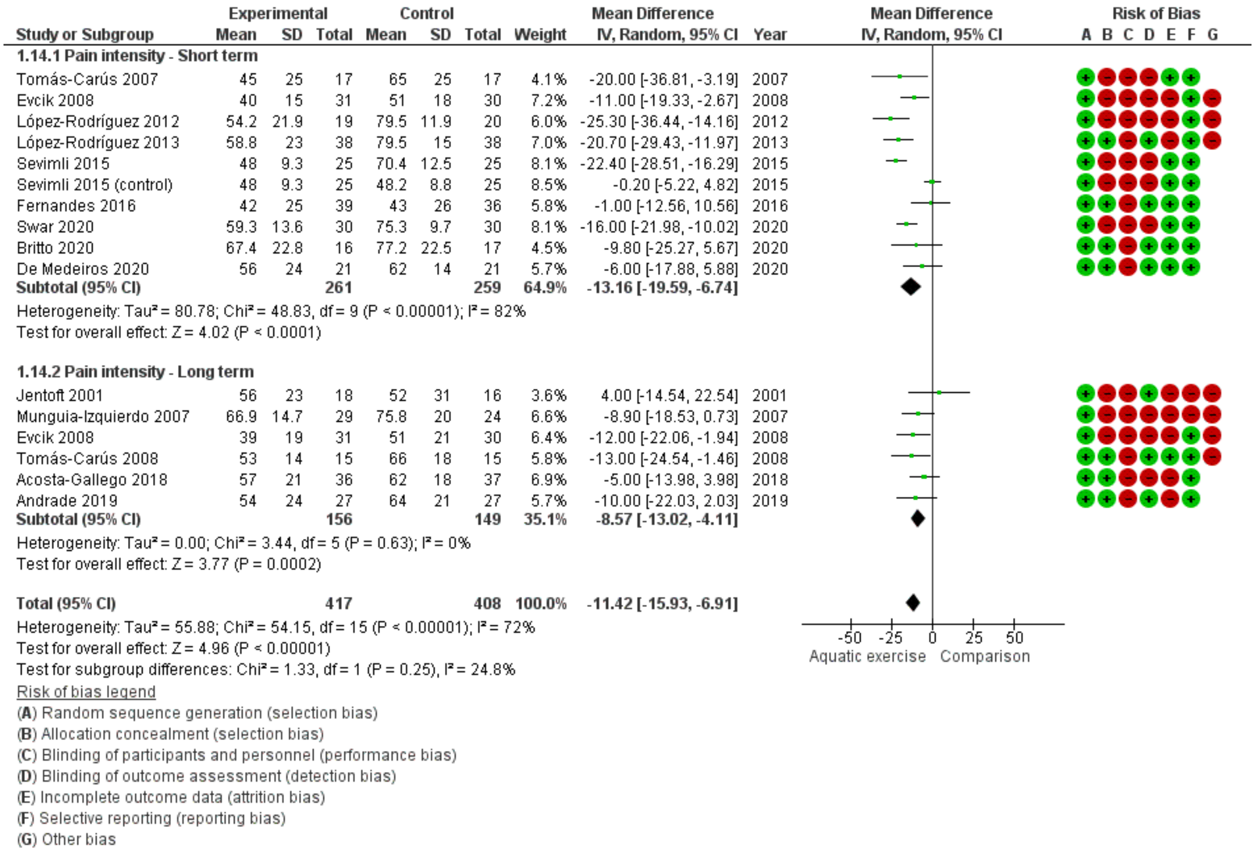

Fig. S.2. Forest plot of the meta-analyses of those studies evaluating the effect of Aerobic Exercise compared to any treatment in relation to pain intensity using Visual Analogue Scale. Short term (≤ 3 months). Long term (> 3 months).

	Aerobic exercise			Comparison				Mean Difference	
**Study or Subgroup**	**Mean**	**SD**	**Total**	**Mean**	**SD**	**Total**	**Weight**	**IV, Random, 95% CI**	**Year**
**2.11.1 Pain intensity - Short term**									
Valim 2003	50	27.1	32	47	25	28	4.6%	3.00 [-10.19, 16.19]	2003
Schachter 2003	47	22.7	29	61	21.6	31	5.0%	-14.00 [-25.23, -2.77]	2003
Schachter 2003 (control)	50	26.4	26	61	21.6	31	4.7%	-11.00 [-23.68, 1.68]	2003
Sencan 2004	40.5	9.1	20	35.5	13.5	20	5.8%	5.00 [-2.14, 12.14]	2004
Sencan 2004 (control)	40.5	9.1	20	62	18.1	20	5.5%	-21.50 [-30.38, -12.62]	2004
Bircan 2008	21.9	18.8	13	26.5	14.1	13	4.7%	-4.60 [-17.37, 8.17]	2008
Bressan 2008	60.3	19.2	6	55.6	22.6	8	3.0%	4.70 [-17.24, 26.64]	2008
Duruturk 2014	31.67	24.71	14	44	15.09	12	4.1%	-12.33 [-27.84, 3.18]	2014
Sevimli 2015 (control)	48.2	8.8	25	48	9.3	25	6.2%	0.20 [-4.82, 5.22]	2015
Sevimli 2015	48.2	8.8	25	70.4	12.5	25	6.0%	-22.20 [-28.19, -16.21]	2015
Fernandes 2016	43	26	36	42	25	39	4.9%	1.00 [-10.56, 12.56]	2016
Gómez-Hernández 2019	73.3	3.8	32	66.8	4.8	32	6.5%	6.50 [4.38, 8.62]	2019
**Subtotal (95% CI)**			**278**			**284**	**61.0%**	**-5.58 [-12.88, 1.72]**	
Heterogeneity: Tau² = 135.04; Chi² = 123.40, df = 11 (P < 0.00001); I² = 91%									
Test for overall effect: Z = 1.50 (P = 0.13)									
**2.11.2 Pain intensity - Long term**									
Schachter 2003	49	25.9	29	56	21.6	31	4.8%	-7.00 [-19.11, 5.11]	2003
Schachter 2003 (control)	50	23.1	26	56	21.6	31	4.9%	-6.00 [-17.69, 5.69]	2003
Valim 2003	50	27.1	32	47	25	28	4.6%	3.00 [-10.19, 16.19]	2003
Sencan 2004 (control)	47.5	12.1	20	58.4	21.1	20	5.1%	-10.90 [-21.56, -0.24]	2004
Sencan 2004	47.5	12.1	20	50.1	19.1	20	5.3%	-2.60 [-12.51, 7.31]	2004
Sañudo 2015	67	22	16	70	17	12	4.3%	-3.00 [-17.45, 11.45]	2015
Acosta-Gallego 2018	62	18	37	57	21	36	5.5%	5.00 [-3.98, 13.98]	2018
Hernando-Garijo 2021	49.2	20	17	64.6	19.2	17	4.6%	-15.40 [-28.58, -2.22]	2021
**Subtotal (95% CI)**			**197**			**195**	**39.0%**	**-4.14 [-8.97, 0.69]**	
Heterogeneity: Tau² = 13.96; Chi² = 9.86, df = 7 (P = 0.20); I² = 29%									
Test for overall effect: Z = 1.68 (P = 0.09)									
**Total (95% CI)**			**475**			**479**	**100.0%**	**-5.15 [-10.28, -0.02]**	
Heterogeneity: Tau² = 104.18; Chi² = 138.43, df = 19 (P < 0.00001); I² = 86%									
Test for overall effect: Z = 1.97 (P = 0.05)									
Test for subgroup differences: Chi² = 0.10, df = 1 (P = 0.75), I² = 0%									

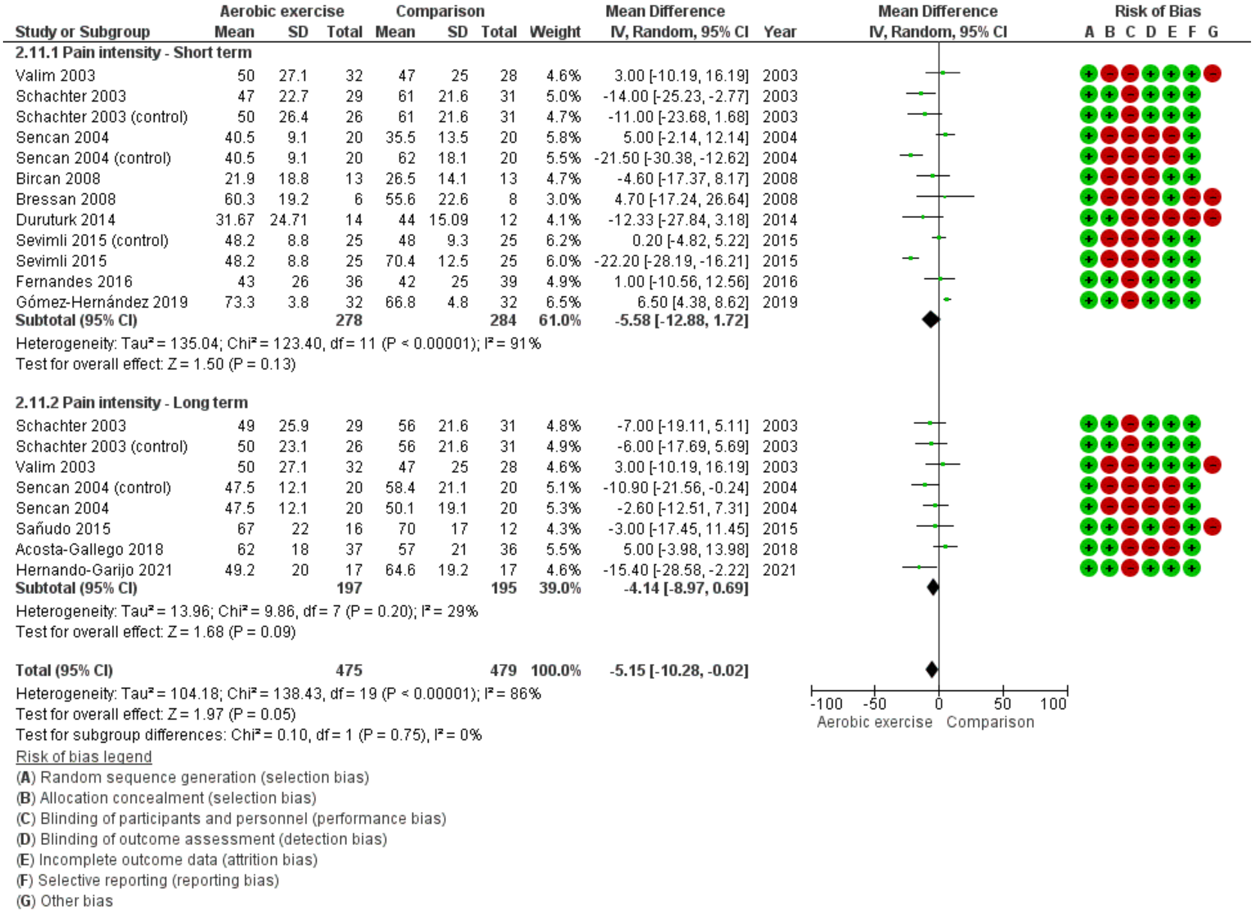

Risk of bias legend
(A) Random sequence generation (selection bias)
(B) Allocation concealment (selection bias)
(C) Blinding of participants and personnel (performance bias)
(D) Blinding of outcome assessment (detection bias)
(E) Incomplete outcome data (attrition bias)
(F) Selective reporting (reporting bias)
(G) Other bias

Fig. S.3. Forest plot of the meta-analyses of those studies evaluating the effect of Resistance Training compared to any treatment in relation to pain intensity using Visual Analogue Scale. Short term (≤ 3 months). Long term (> 3 months).

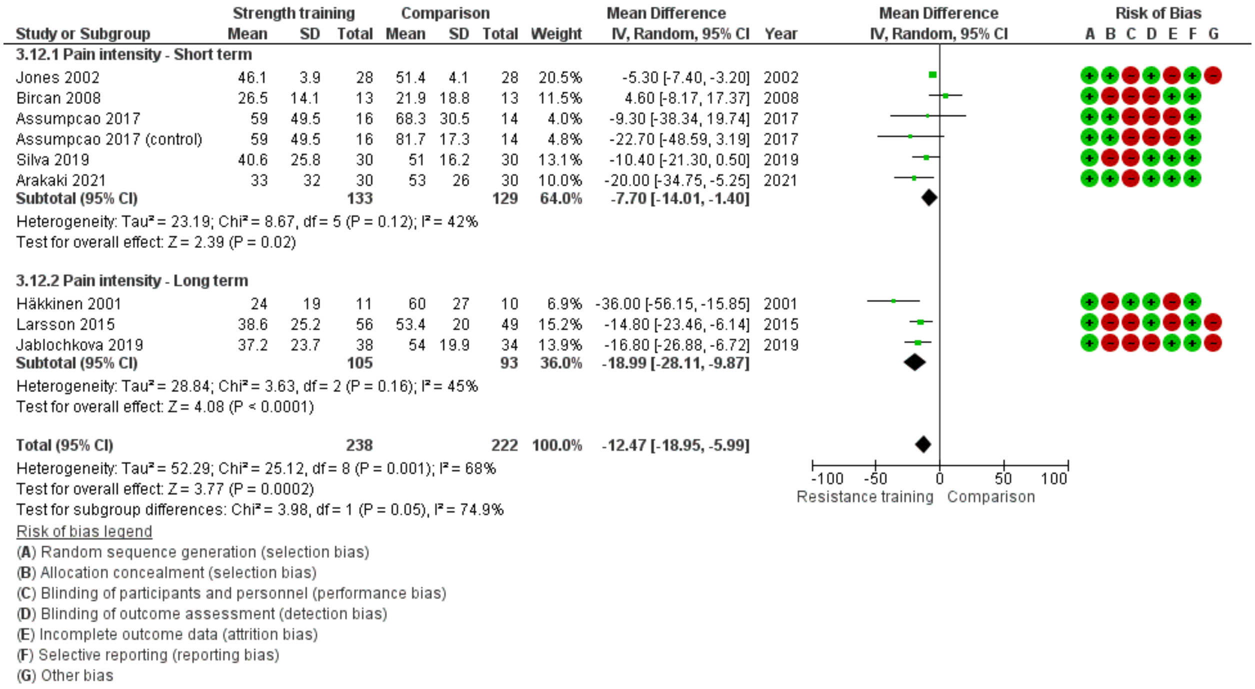

Fig. S.4. Forest plot of the meta-analyses of those studies evaluating the effect of Flexibility compared to any treatment in relation to pain intensity using Visual Analogue Scale. Short term (≤ 3 months). Long term (> 3 months).

	Experimental			Control				Mean Difference		Risk of Bias						
**Study or Subgroup**	**Mean**	**SD**	**Total**	**Mean**	**SD**	**Total**	**Weight**	**IV, Random, 95% CI**	**Year**	**A**	**B**	**C**	**D**	**E**	**F**	**G**
**4.12.1 Pain intensity - Short term**																
Jones 2002	51.4	4.1	28	46.1	3.9	28	13.3%	5.30 [3.20, 7.40]	2002	+	+	−	+	−	+	−
Valim 2003	47	25	28	50	27.1	28	7.2%	-3.00 [-16.66, 10.66]	2003	+	−	−	+	+	+	−
Bressan 2008	55.6	22.6	8	60.3	19.2	6	4.2%	-4.70 [-26.64, 17.24]	2008	+	−	−	−	+	−	−
Altan 2009	60	21	24	41	17	25	8.8%	19.00 [8.28, 29.72]	2009	+	+	−	+	+	+	
López-Rodríguez 2012	79.5	11.9	20	54.2	21.9	19	8.6%	25.30 [14.16, 36.44]	2012	+	−	−	−	−	+	−
López-Rodríguez 2013	79.5	15	38	58.8	23	38	10.0%	20.70 [11.97, 29.43]	2013	+	+	−	+	−	+	−
Genc 2015	63.93	19.01	25	55.56	20.09	25	8.7%	8.37 [-2.47, 19.21]	2015	+	−	−	−	+	+	−
Assumpcao 2017	68.3	30.5	14	59	49.5	16	2.7%	9.30 [-19.74, 38.34]	2017	+	+	−	−	−	+	
Assumpcao 2017 (control)	68.3	30.5	14	81.7	17.3	14	5.3%	-13.40 [-31.77, 4.97]	2017	+	+	−	−	−	+	
De Carvalho 2020	51.6	19.6	19	44.4	13	16	8.7%	7.20 [-3.67, 18.07]	2020	+	−	−	+	−	+	
Arakaki 2021	53	26	30	33	32	30	6.7%	20.00 [5.25, 34.75]	2021	+	+	−	+	+	+	
**Subtotal (95% CI)**			**248**			**245**	**84.2%**	**9.95 [3.74, 16.15]**								
Heterogeneity: Tau² = 66.18; Chi² = 38.11, df = 10 (P < 0.0001); I² = 74%																
Test for overall effect: Z = 3.14 (P = 0.002)																
**4.12.2 Pain intensity - Long term**																
Valim 2003	65	21	24	52	25	25	7.6%	13.00 [0.09, 25.91]	2003	+	−	−	+	+	+	−
Altan 2009	48	21.8	28	34.2	25	32	8.2%	13.80 [1.96, 25.64]	2009	+	+	−	+	+	+	
**Subtotal (95% CI)**			**52**			**57**	**15.8%**	**13.43 [4.71, 22.16]**								
Heterogeneity: Tau² = 0.00; Chi² = 0.01, df = 1 (P = 0.93); I² = 0%																
Test for overall effect: Z = 3.02 (P = 0.003)																
**Total (95% CI)**			**300**			**302**	**100.0%**	**10.57 [5.18, 15.96]**								
Heterogeneity: Tau² = 55.87; Chi² = 40.16, df = 12 (P < 0.0001); I² = 70%																
Test for overall effect: Z = 3.84 (P = 0.0001)																
Test for subgroup differences: Chi² = 0.41, df = 1 (P = 0.52), I² = 0%																

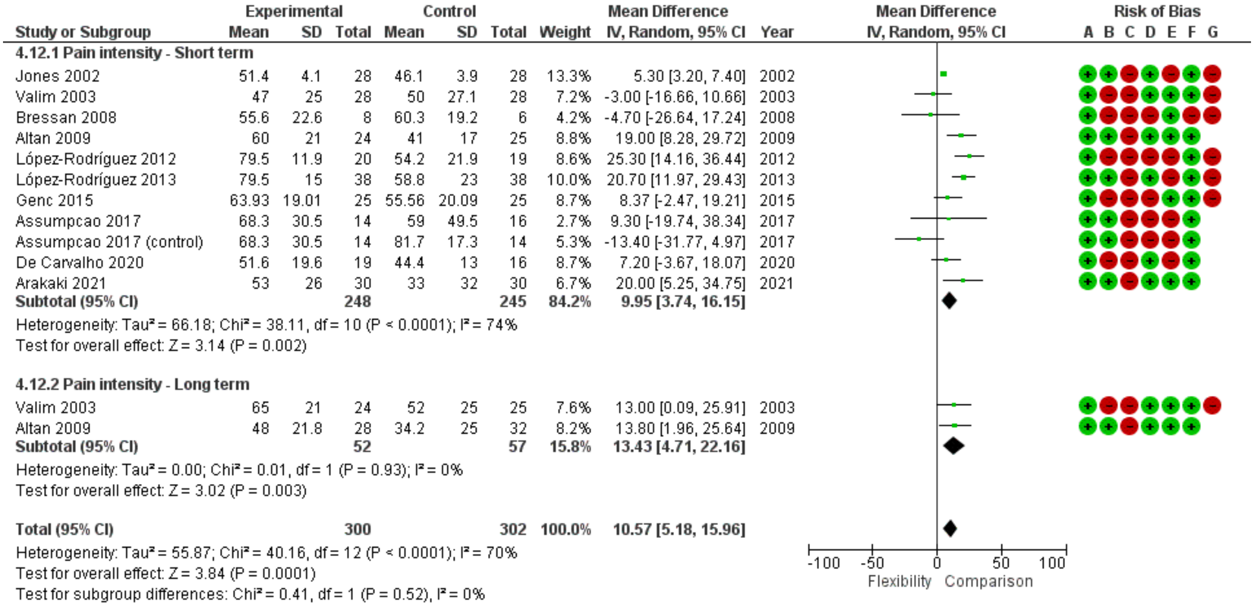

Risk of bias legend
(A) Random sequence generation (selection bias)
(B) Allocation concealment (selection bias)
(C) Blinding of participants and personnel (performance bias)
(D) Blinding of outcome assessment (detection bias)
(E) Incomplete outcome data (attrition bias)
(F) Selective reporting (reporting bias)
(G) Other bias

Fig. S.5. Forest plot of the meta-analyses of those studies evaluating the effect of Mixed Exercise compared to any treatment in relation to pain intensity using Visual Analogue Scale. Short term (≤ 3 months). Long term (> 3 months).

	Experimental			Control				Mean Difference	
Study or Subgroup	Mean	SD	Total	Mean	SD	Total	Weight	IV, Random, 95% CI	Year
**8.15.1 Pain intensity - Short term**									
Evcik 2008	51	18	30	40	15	31	6.4%	11.00 [2.67, 19.33]	2008
Genc 2015	55.56	20.09	25	63.96	19.01	25	6.2%	-8.40 [-19.24, 2.44]	2015
Sevimli 2015	70.4	12.5	25	48	9.3	25	6.6%	22.40 [16.29, 28.51]	2015
Sevimli 2015 (control)	70.4	12.5	25	48.2	8.8	25	6.7%	22.20 [16.21, 28.19]	2015
Gómez-Hernández 2019	57.7	4	32	67.1	4.2	32	6.9%	-9.40 [-11.41, -7.39]	2019
Gulsen 2020	45.6	19.4	10	39.2	10.1	10	5.8%	6.40 [-7.16, 19.96]	2020
Swar 2020	75.3	9.7	30	59.3	13.6	30	6.7%	16.00 [10.02, 21.98]	2020
Atan 2020 (control)	43.6	24.3	19	78.8	13.6	17	5.9%	-35.20 [-47.90, -22.50]	2020
Britto 2020	77.2	22.5	17	67.4	22.8	16	5.6%	9.80 [-5.67, 25.27]	2020
Atan 2020	42.1	19.3	19	78.8	13.6	17	6.2%	-36.70 [-47.52, -25.88]	2020
Rodríguez-Mansilla 2021	73.1	19.3	47	61.6	25.6	47	6.4%	11.50 [2.33, 20.67]	2021
Rodríguez-Mansilla 2021 (control)	67.9	14.3	47	61.6	25.6	47	6.4%	6.30 [-2.08, 14.68]	2021
**Subtotal (95% CI)**			**326**			**322**	**75.8%**	**1.63 [-8.73, 12.00]**	
Heterogeneity: Tau² = 311.43; Chi² = 303.17, df = 11 (P < 0.00001); I² = 96%									
Test for overall effect: Z = 0.31 (P = 0.76)									
**8.15.2 Pain intensity - Long term**									
Jentoft 2001	52	31	16	56	23	18	5.1%	-4.00 [-22.54, 14.54]	2001
Rooks 2007 (1)	52	20	51	59	22	50	6.5%	-7.00 [-15.20, 1.20]	2007
Rooks 2007 (2)	48	25	51	59	22	50	6.4%	-11.00 [-20.18, -1.82]	2007
Evcik 2008	51	21	30	39	19	31	6.3%	12.00 [1.94, 22.06]	2008
**Subtotal (95% CI)**			**148**			**149**	**24.2%**	**-2.55 [-13.35, 8.26]**	
Heterogeneity: Tau² = 88.16; Chi² = 12.37, df = 3 (P = 0.006); I² = 76%									
Test for overall effect: Z = 0.46 (P = 0.64)									
**Total (95% CI)**			**474**			**471**	**100.0%**	**0.67 [-7.69, 9.02]**	
Heterogeneity: Tau² = 263.70; Chi² = 315.75, df = 15 (P < 0.00001); I² = 95%									
Test for overall effect: Z = 0.16 (P = 0.88)									
Test for subgroup differences: Chi² = 0.30, df = 1 (P = 0.58), I² = 0%									

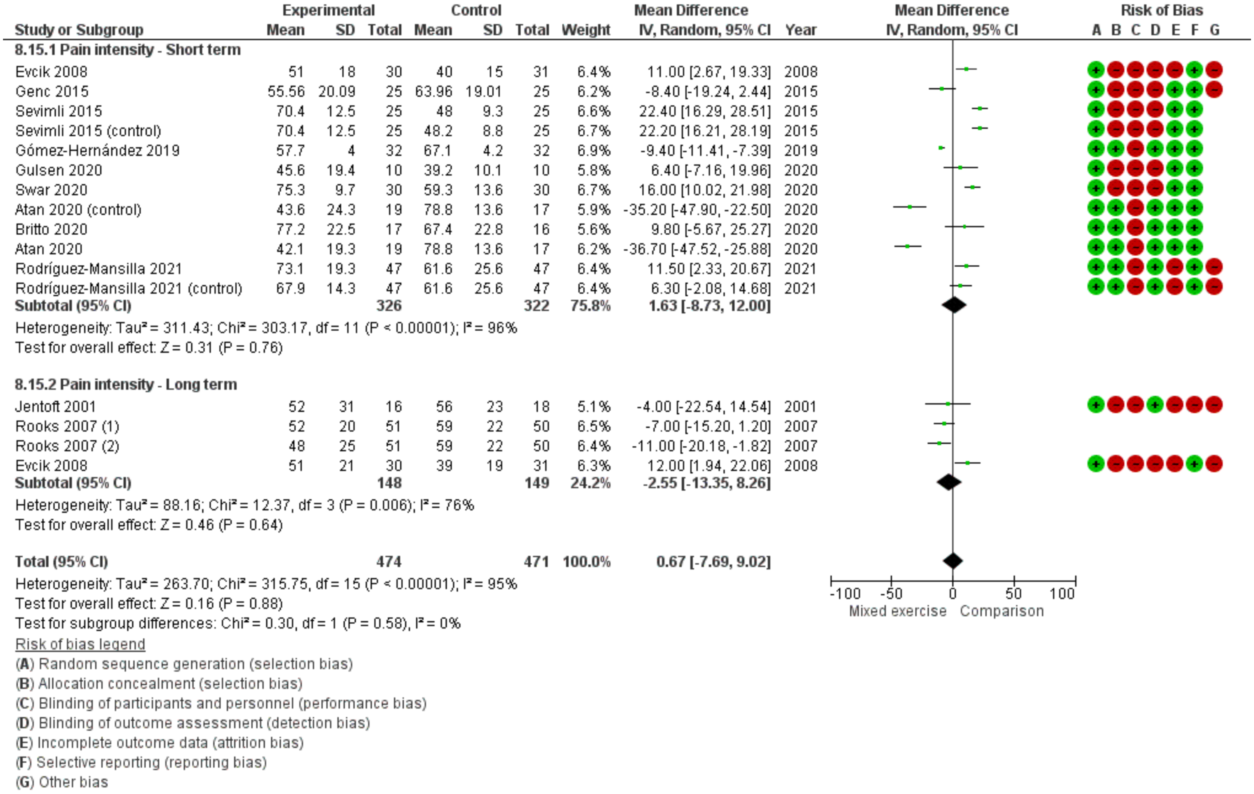

Risk of bias legend
(A) Random sequence generation (selection bias)
(B) Allocation concealment (selection bias)
(C) Blinding of participants and personnel (performance bias)
(D) Blinding of outcome assessment (detection bias)
(E) Incomplete outcome data (attrition bias)
(F) Selective reporting (reporting bias)
(G) Other bias

Fig. S.6. Forest plot of the meta-analyses of those studies evaluating the effect of Core Stability compared to any treatment in relation to pain intensity using Visual Analogue Scale. Short term (≤ 3 months). Long term (> 3 months).

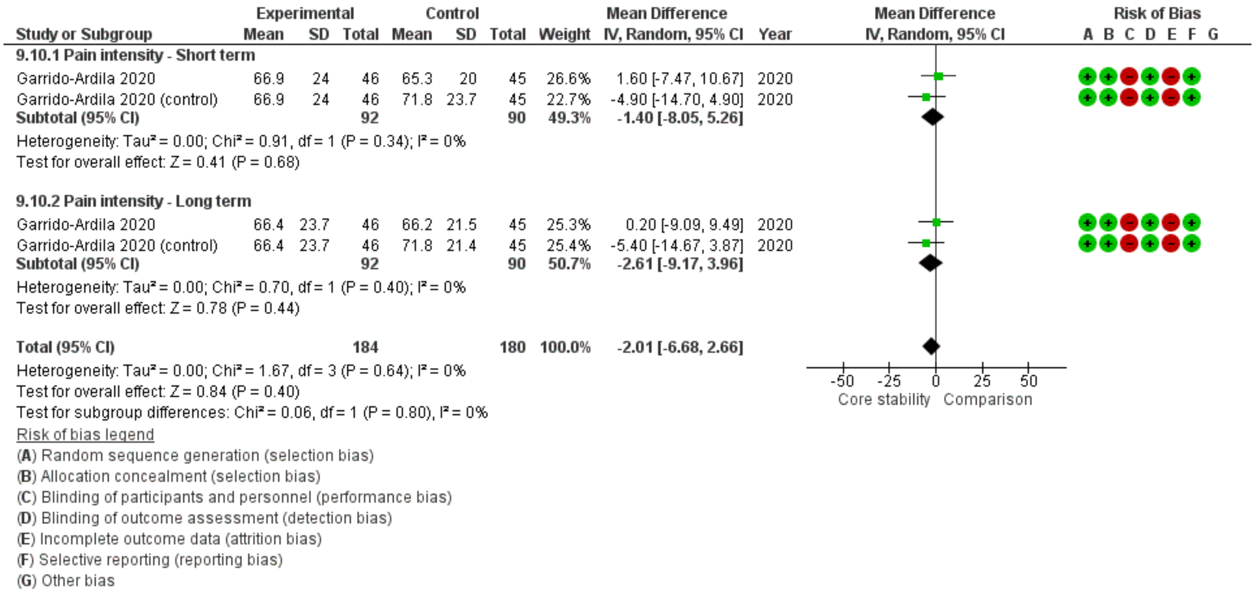

Fig. S.7. Forest plot of the meta-analyses of those studies evaluating the effect of Dance compared to any treatment in relation to pain intensity using Visual Analogue Scale. Short term (≤ 3 months). Long term (> 3 months).

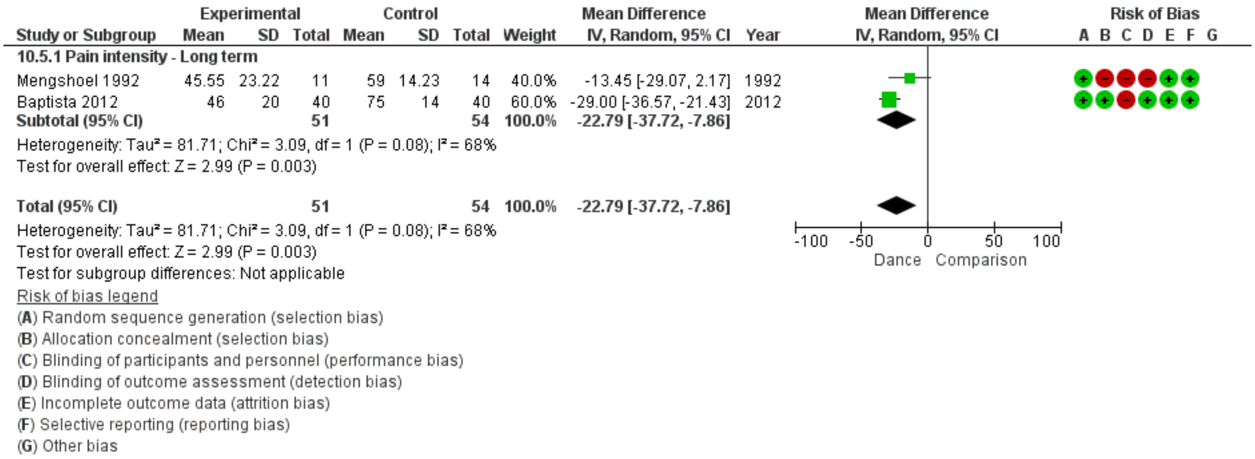

Fig. S.8. Forest plot of the meta-analyses of those studies evaluating the effect of Functional Training compared to any treatment in relation to pain intensity using Visual Analogue Scale. Short term (≤ 3 months). Long term (> 3 months).

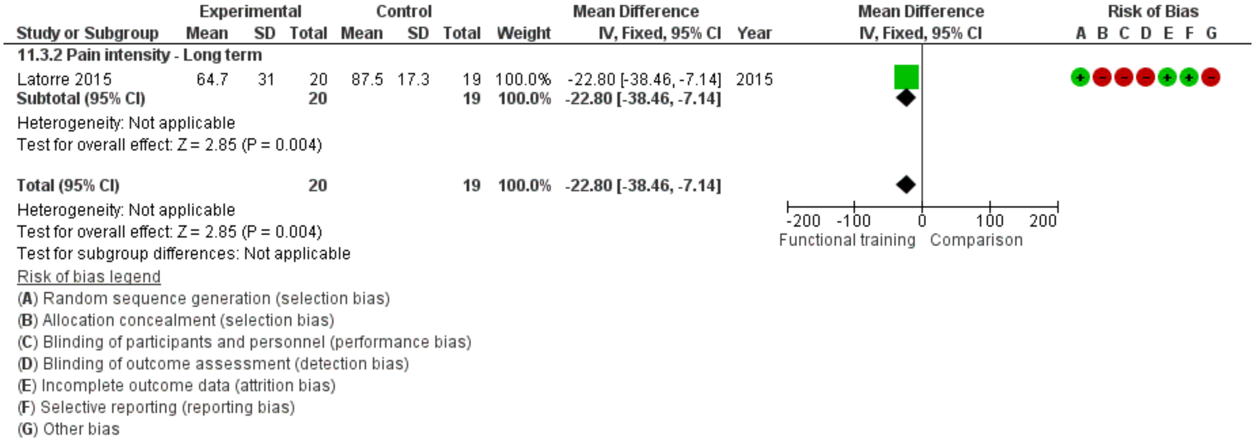

Fig. S.9. Forest plot of the meta-analyses of those studies evaluating the effect of Pilates compared to any treatment in relation to pain intensity using Visual Analogue Scale. Short term (≤ 3 months). Long term (> 3 months).

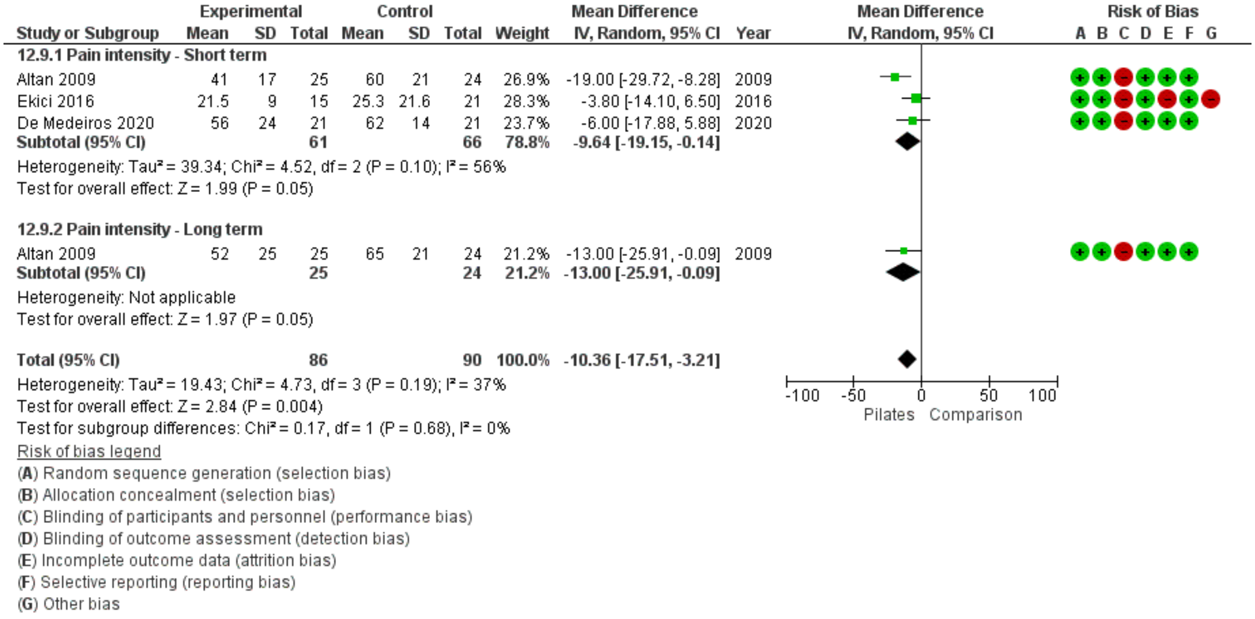

Fig. S.10. Forest plot of the meta-analyses of those studies evaluating the effect of Balance Exercise compared to any treatment in relation to pain intensity using Visual Analogue Scale. Short term (≤ 3 months). Long term (> 3 months).

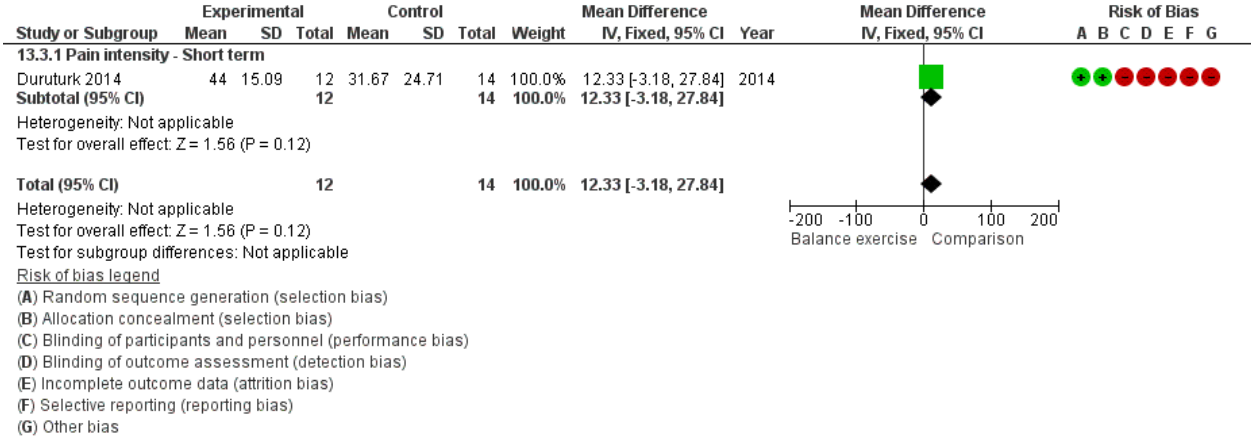

Fig. S.11. Forest plot of the meta-analyses of those studies evaluating the effect of Qigong compared to any treatment in relation to pain intensity using Visual Analogue Scale. Short term (≤ 3 months). Long term (> 3 months).

Study or Subgroup	Experimental Mean	Experimental SD	Experimental Total	Control Mean	Control SD	Control Total	Weight	Mean Difference IV, Random, 95% CI	Year
**14.3.1 Pain intensity - Short term**									
Sarmento 2020	31	17	10	68	21	10	26.7%	-37.00 [-53.75, -20.25]	2020
Rodríguez-Mansilla 2021	61.6	25.6	47	73.1	19.3	47	36.2%	-11.50 [-20.67, -2.33]	2021
Rodríguez-Mansilla 2021 (control)	61.6	25.6	47	67.9	14.3	47	37.1%	-6.30 [-14.68, 2.08]	2021
**Subtotal (95% CI)**			**104**			**104**	**100.0%**	**-16.39 [-30.57, -2.20]**	
Heterogeneity: Tau² = 122.91; Chi² = 10.35, df = 2 (P = 0.006); I² = 81%									
Test for overall effect: Z = 2.26 (P = 0.02)									
**Total (95% CI)**			**104**			**104**	**100.0%**	**-16.39 [-30.57, -2.20]**	
Heterogeneity: Tau² = 122.91; Chi² = 10.35, df = 2 (P = 0.006); I² = 81%									
Test for overall effect: Z = 2.26 (P = 0.02)									
Test for subgroup differences: Not applicable									

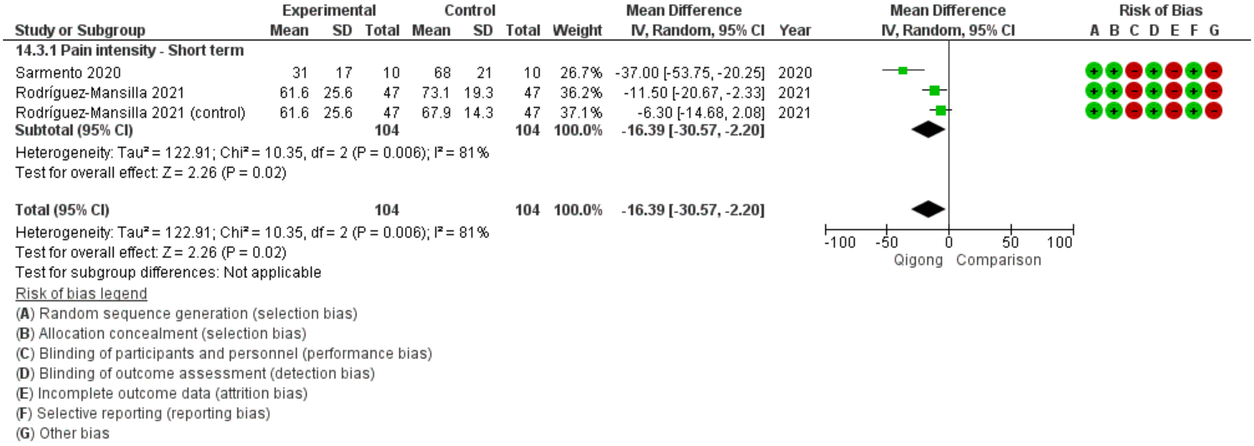

Risk of bias legend
(A) Random sequence generation (selection bias)
(B) Allocation concealment (selection bias)
(C) Blinding of participants and personnel (performance bias)
(D) Blinding of outcome assessment (detection bias)
(E) Incomplete outcome data (attrition bias)
(F) Selective reporting (reporting bias)
(G) Other bias

Fig. S.12. Forest plot of the meta-analyses of those studies evaluating the effect of Tai Chi compared to any treatment in relation to pain intensity using Visual Analogue Scale. Short term (≤ 3 months). Long term (> 3 months).

Study or Subgroup	Experimental Mean	Experimental SD	Experimental Total	Control Mean	Control SD	Control Total	Weight	Mean Difference IV, Fixed, 95% CI	Year
**15.2.1 Pain intensity - Short term**									
Wong 2018	53	3	18	70	5	19	100.0%	-17.00 [-19.64, -14.36]	2018
**Subtotal (95% CI)**			**18**			**19**	**100.0%**	**-17.00 [-19.64, -14.36]**	
Heterogeneity: Not applicable									
Test for overall effect: Z = 12.62 (P < 0.00001)									
**Total (95% CI)**			**18**			**19**	**100.0%**	**-17.00 [-19.64, -14.36]**	
Heterogeneity: Not applicable									
Test for overall effect: Z = 12.62 (P < 0.00001)									
Test for subgroup differences: Not applicable									

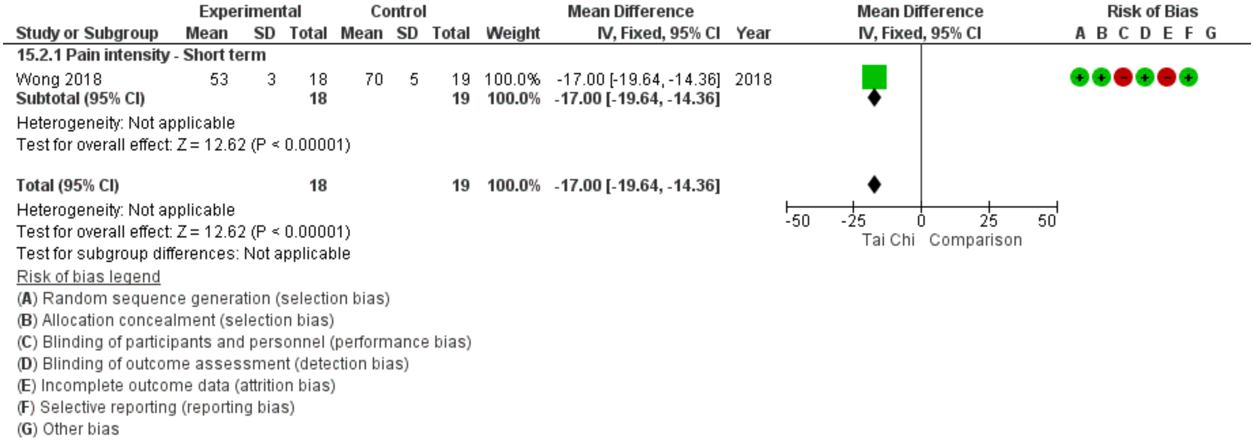

Risk of bias legend
(A) Random sequence generation (selection bias)
(B) Allocation concealment (selection bias)
(C) Blinding of participants and personnel (performance bias)
(D) Blinding of outcome assessment (detection bias)
(E) Incomplete outcome data (attrition bias)
(F) Selective reporting (reporting bias)
(G) Other bias

Fig. S.13. Forest plot of the meta-analyses of those studies evaluating the effect of Virtual Reality compared to any treatment in relation to pain intensity using Visual Analogue Scale. Short term (≤ 3 months). Long term (> 3 months).

	Experimental			Control				Mean Difference	
Study or Subgroup	Mean	SD	Total	Mean	SD	Total	Weight	IV, Fixed, 95% CI	Year
**17.9.1 Pain intensity - Short term**									
Collado-Mateo 2017	53.7	18.9	42	67	22	41	32.4%	-13.30 [-22.13, -4.47]	2017
De Carvalho 2020	44.4	13	16	51.6	19.6	19	21.4%	-7.20 [-18.07, 3.67]	2020
Gulsen 2020	39.2	10.1	10	45.6	19.4	10	13.7%	-6.40 [-19.96, 7.16]	2020
**Subtotal (95% CI)**			**68**			**70**	**67.5%**	**-9.96 [-16.08, -3.85]**	
Heterogeneity: Chi² = 1.06, df = 2 (P = 0.59); I² = 0%									
Test for overall effect: Z = 3.19 (P = 0.001)									
**17.9.2 Pain intensity - Long term**									
Villafaina 2019	58.88	16.34	28	68.2	17.29	28	32.5%	-9.32 [-18.13, -0.51]	2019
**Subtotal (95% CI)**			**28**			**28**	**32.5%**	**-9.32 [-18.13, -0.51]**	
Heterogeneity: Not applicable									
Test for overall effect: Z = 2.07 (P = 0.04)									
**Total (95% CI)**			**96**			**98**	**100.0%**	**-9.75 [-14.78, -4.73]**	
Heterogeneity: Chi² = 1.08, df = 3 (P = 0.78); I² = 0%									
Test for overall effect: Z = 3.80 (P = 0.0001)									
Test for subgroup differences: Chi² = 0.01, df = 1 (P = 0.91), I² = 0%									

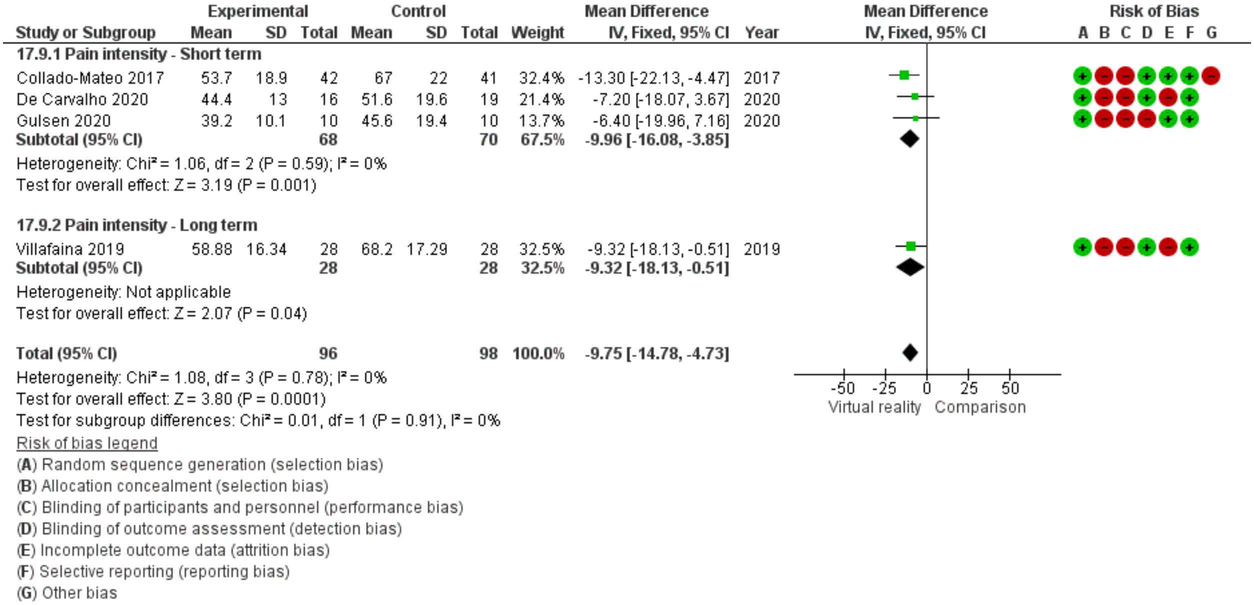

Risk of bias legend
(A) Random sequence generation (selection bias)
(B) Allocation concealment (selection bias)
(C) Blinding of participants and personnel (performance bias)
(D) Blinding of outcome assessment (detection bias)
(E) Incomplete outcome data (attrition bias)
(F) Selective reporting (reporting bias)
(G) Other bias

Fig. S.14. Forest plot of the meta-analyses of those studies evaluating the effect of Yoga compared to any treatment in relation to pain intensity using Visual Analogue Scale. Short term (≤ 3 months). Long term (> 3 months).

	Experimental			Control				Mean Difference		Risk of Bias
**Study or Subgroup**	**Mean**	**SD**	**Total**	**Mean**	**SD**	**Total**	**Weight**	**IV, Fixed, 95% CI**	**Year**	**A B C D E F G**
**18.4.1 Pain intensity - Short term**										
Carson 2010	41.2	20.5	25	51.4	22.7	28	100.0%	-10.20 [-21.83, 1.43]	2010	+ + − + + −
**Subtotal (95% CI)**			**25**			**28**	**100.0%**	**-10.20 [-21.83, 1.43]**		
Heterogeneity: Not applicable										
Test for overall effect: Z = 1.72 (P = 0.09)										
**Total (95% CI)**			**25**			**28**	**100.0%**	**-10.20 [-21.83, 1.43]**		
Heterogeneity: Not applicable										
Test for overall effect: Z = 1.72 (P = 0.09)										
Test for subgroup differences: Not applicable										

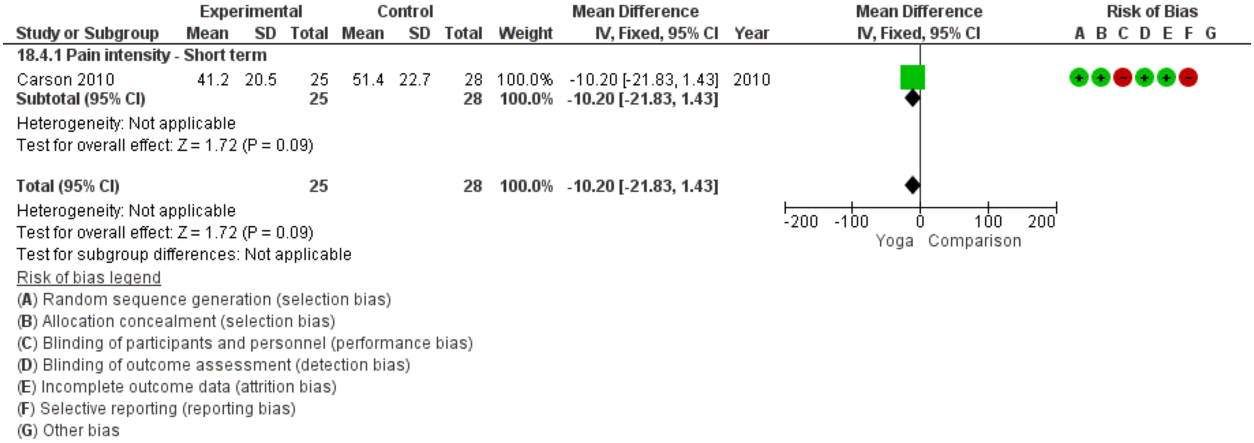

Risk of bias legend
(A) Random sequence generation (selection bias)
(B) Allocation concealment (selection bias)
(C) Blinding of participants and personnel (performance bias)
(D) Blinding of outcome assessment (detection bias)
(E) Incomplete outcome data (attrition bias)
(F) Selective reporting (reporting bias)
(G) Other bias

Table S.4. Summary of the results obtained. Number of participants by type of intervention and temporality investigated, number of studies, and diagnostic criteria used.

**Total studies in review (n)**	**Excluded for meta-analysis (n)**	**Analyzed (n)**	
61 (n = 3,581)	10 (n = 708)	51 (n = 2,873)	
**Diagnostic criteria**			
**ACR1990**	**ACR2010**	**ACR2016**	
48/60	13/60	2/60	
**Number of studies by modality and temporality**			
	Short-term Number of studies (n)	Long-term Number of studies (n)	Total Number of studies (n)
**Aerobic Exercise**	9 (233)	6 (177)	12 (295)
**Aquatic Exercise**	9 (236)	6 (156)	13 (345)
**Resistance Training**	5 (117)	3 (105)	8. (222)
**Flexibility**	9 (234)	2 (52)	9 (234)
**Mixed Exercise**	9 (255)	3 (164)	11 (402)
**Core Stability**	1 (46)	1 (46)	1 (46)
**Dance**	-	2 (51)	2 (51)
**Functional Training**	-	1 (20)	1 (20)
**Pilates**	3 (61)	1 (25)	3 (61)
**Balance Exercise**	1 (12)	-	1 (12)
**Qigong**	1 (57)	-	1 (57)
**Tai Chi**	1 (18)	-	1 (18)
**Vibration Exercise**	-	-	-
**Virtual Reality**	3 (68)	1 (28)	4 (96)
**Yoga**	1 (25)	-	1 (25)
**Acupuncture**	1 (45)	1 (45)	1 (45)
**Combined Therapy**	4 (59)	3 (63)	6 (112)
**Usual Care**	15 (307)	18 (312)	26 (479)
**Education**	-	1 (27)	1 (27)
**Manual Therapy**	1 (21)	-	1 (21)
**Drugs Therapy**	1 (20)	1 (20)	1 (20)
**Relaxation**	1 (27)	4 (147)	4 (147)
**Sham**	2 (30)	1 (20)	2 (30)
**TOTAL**	n = 1.812	n = 1.395	n = 2.653
**Intervention modalities**			
**Supervised**	**Home-exercise**	**Telerehabilitation**	**Not specified**
48 (2547)	7 (659)	1 (34)	5 (389)
**Nationality**			
**Australia**	**Finland**	**Norway**	**USA**

1 (25)	3 (72)	1 (44)	4 (356)
**Netherlands**	**Canada**	**Brazil**	**Turkey**
1 (37)	1 (143)	15 (849)	13 (630)
**Spain**	**Sweden**	**Korea**	**Egypt**
17 (1049)	3 (330)	1 (37)	1 (60)

Table S.5. Exercise interventions and comparison groups.

Intervention	Definition
**Aerobic Exercise**	Exercises training such as walking, cycling, and jogging in any land-based mode that is designed to improve the cardiovascular system.
**Aquatic Exercise**	Exercise training performed in deep or shallow water.
**Resistance Training**	Exercise training designed to improve muscle strength, tone, mass, or endurance.
**Flexibility**	Exercise training designed to improve the range of movement by holding or stretching the body in specific positions.
**Mixed Exercise**	Exercise training including two or more types of exercise such as aerobic, strengthening, or flexibility exercises.
**Core Stability**	Exercise training designed to improve the control, coordination, and strength of the lumbopelvic region.
**Dance**	Type of aerobic exercise where movement is performed with music.
**Functional Training**	Exercise training focusing on activities of daily living, avoiding traditional resistance/strength training.
**Pilates**	Exercise training following traditional Pilates principles such as centering, concentration, control, precision, flow, and breathing.
**Balance Exercise**	Exercise training designed to improve the neuromuscular system.
**Qigong**	Exercise training following traditional Qigong principles such as movement, meditation, and breathing regulation.
**Tai Chi**	Exercise training following traditional Tai Chi principles such as slow movements, breathing, and mental concentration.
**Vibration Exercise**	Exercise training performed on a vibrating platform.
**Virtual Reality**	Exercise training performed using a virtual reality device.
**Yoga**	Exercise training following traditional Yoga principles such as stretching, strengthening, controlled breathing and meditation.
**Comparators**	**Definition**
**Acupuncture**	Introduction of very fine needles in certain points of the human body according to the principles of Chinese medicine.
**Combined Therapy**	Intervention that combines different exercise modalities with other therapeutic modalities.
**Usual Care**	Participants were allowed to continue their normal daily activities as earlier, to use their usual treatment and to visit medical professionals if needed (no intervention).
**Education**	Biopsychosocial intervention with verbal or written guidance, online or in person.
**Manual Therapy**	Any intervention using passive manual techniques
**Drugs Therapy**	Any intervention that uses drugs or medications.
**Relaxation**	Intervention performed with the aim of controlling stress and improving general well-being.
**Sham**	Any intervention in which the participant is blinded to the sham treatment received.

Table S.6. Assessment of local incoherence

Comparisons	NMA	Direct	Indirect	Difference direct-indirect	z-statistic	p-value
Acupuncture vs. Core Stability	-0.88	-1.6	7.49	-9.09	-0.21	0.83
Acupuncture vs. Usual Care	-7.22	-6.5	-15.59	9.09	0.21	0.83
Aerobic Exercise vs. Aquatic Exercise	12.12	2.33	19.18	-16.85	-1.49	0.14
Aerobic Exercise vs. Usual Care	-16.95	-12.54	-19.70	7.16	0.62	0.53
Aerobic Exercise vs. Flexibility	-6.61	3.69	-11.41	15.10	1.23	0.22
Aerobic Exercise vs. Mixed Exercise	0.63	-5.90	5.39	-11.29	-1.06	0.29
Aerobic Exercise vs. Pharmacology	2.85	5	-26.89	31.89	0.7	0.48
Aerobic Exercise vs. Sham	-26.18	-21.5	-36.88	15.38	0.7	0.48
Aerobic Exercise vs. Resistance Training	2.28	-4.6	5.99	-10.58	-0.66	0.51
Aquatic Exercise vs. Usual Care	-29.07	-20	-30.99	10.99	0.71	0.48
Aquatic Exercise vs. Flexibility	-18.73	-22.91	-15.93	-6.98	-0.62	0.53
Aquatic Exercise vs. Mixed Exercise	-11.5	-15.24	-4.69	-10.55	-1.03	0.30
Aquatic Exercise vs. Pilates	-2.80	-6	0.94	-6.94	-0.37	0.71
Core Stability vs. Combined Therapy	21.27	19.2	23.85	-4.66	-0.21	0.83
Core Stability vs. Usual Care	-6.34	-4.9	-9.56	4.66	0.21	0.83
Combined Therapy vs. Mixed Exercise	-10.03	-10.91	-6.28	-4.66	-0.21	0.83
Flexibility vs. Usual Care	-10.34	-13.33	-9.70	-3.63	-0.23	0.82
Mixed Exercise vs. Usual Care	-17.59	-24.61	-10.66	-13.95	-1.38	0.167
Qigong vs. Usual Care	-21.42	-11.5	-38.08	26.58	1.33	0.18
Resistance Training vs. Usual Care	-19.24	-22.67	-18.26	-4.41	-0.22	0.82
Virtual Reality vs. Usual Care	-17.97	-13.3	-21.45	8.145	0.51	0.61
Flexibility vs. Mixed Exercise	7.25	8.4	6.95	1.452	0.1	0.918
Flexibility vs. Pilates	15.93	19	12.06	6.94	0.37	0.71
Flexibility vs. Resistance Training	8.90	10.98	2.24	8.74	0.54	0.59
Flexibility vs. Virtual Reality	7.63	7.2	7.93	-0.73	-0.04	0.96
Mixed Exercise vs. Qigong	3.83	6.3	-0.428	6.73	0.34	0.74
Mixed Exercise vs. Virtual Reality	0.38	6.4	-2.96	9.36	0.57	0.57
Pharmacology vs. Sham	-29.04	-26.5	-55.85	29.35	0.7	0.48
Qigong vs. Sham	-30.65	-37	-21.62	-15.38	-0.7	0.48

Table S.6A. Separate Indirect from Direct Evidence (SIDE) results. Short term (≤ 3 months).

Comparisons	NMA	Direct	Indirect	Difference direct-indirect	z-statistic	p-value
Aerobic Exercise vs. Aquatic Exercise	3.76	5	2.54	2.46	0.38	0.70
Aerobic Exercise vs. Usual Care	-7.27	-7.89	-5.42	-2.46	-0.38	0.70
Aquatic Exercise vs. Usual Care	-11.03	-10.42	-12.89	2.46	0.38	0.70
Combined Therapy vs. Education	-10.73	-10	-34.45	24.45	1.33	0.18
Combined Therapy vs. Mixed Exercise	-2.71	-2.64	-19.61	16.96	0.35	0.73
Education vs. Mixed Exercise	8.03	8.78	-15.69	24.46	1.37	0.17

Table S.6B. Separate Indirect from Direct Evidence (SIDE) results. Long term (> 3 months).

Table S.7. Summary of results of the pairwise meta-analyses for the Visual Analogic Scale.

**Exercise treatments**	***Short-term***	**Long-term**	**Total**
Aquatic Exercise	MD = -13.16; 95% CI (-19.59, -6.74)	MD = -8.57; 95% CI (-15.93, -6.91)	MD = -11.42; 95% CI (-15.93, -6.91),
Aerobic Exercise	MD = -5.75; 95% CI (-13.05, 1.56)	MD = -6.07; 95% CI (-10.57, -1.57)	MD = -5.84; 95% CI (-11.23, -0.46)
Resistance Training	MD = -7.70; 95% CI (-14.01, -1.40)	MD = -18.99; 95% CI (-28.11, -9.87)	MD = -12.47; 95% CI (-18.95, -5.99)
Flexibility	MD = 9.95; 95% CI (3.74, 16.15)	MD = 13.43; 95% CI (-4.71, 22.16)	MD = 10.57; 95% CI (5.18, 15.96)
Mixed Exercise	MD = 1.63; 95% CI (-8.73, 12.00)	MD = -2.55; 95% CI (-13.35, 8.26)	MD = 0.67; 95% CI (-7.69, 9.02)
Core Stability	MD = -1.40; 95% CI (-8.05, 5.26)	MD = -2.61; 95% CI (-9.17, 3.96)	MD = -2.01; 95% CI (-6.68, 2.66)
Dance	-	MD = -22.79; 95% CI (-37.72, -7.86)	-
Functional Training	-	MD = -22.80; 95% CI (-38.46, -7.14)	-
Pilates	MD = -9.64; 95% CI (-19.15, -0.14)	MD = -13.00; 95% CI (-25.91, -0.09)	MD = -10.36; 95% CI (-17.51, -3.21)
Balance Exercise	MD = 12.33; 95% CI (-3.18, 27.84)	-	-
Qigong	MD = -16.39; 95% CI (-30.57, -2.20)	-	-
Tai Chi	MD = -17.00; 95% CI (-19.64, -14.36)	-	-
Virtual Reality	MD = -9.96; 95% CI (-16.08, -3.85)	MD = -9.32; 95% CI (-18.13, -0.51)	MD = -9.75; 95% CI (-14.78, -4.73)
Yoga	MD = -10.20; 95% CI (-21.83, 1.43)	-	-

Statistically significant results are shown in blue. Statistically significant results in favor of the comparison group are shown in orange. The results in dark blue (white numbers) indicate those that were clinically relevant. A clinically important difference was interpreted as a difference in pain of 15 points out of 100. MD, mean difference; CI, confidence interval.

Table S.8. Summary of results of the PEDro scale.

Ref	1	2	3	4	5	6	7	8	9	10	11	PEDro
Mengshoel 1992[42]	Yes	Yes	No	Yes	No	No	No	Yes	Yes	Yes	Yes	7/11
Hakkinen 2001[43]	Yes	Yes	No	Yes	No	No	No	Yes	Yes	Yes	Yes	7/11
Jentoft 2001[44]	Yes	Yes	No	Yes	No	No	Yes	No	No	No	Yes	5/11
Jones 2002[45]	Yes	Yes	Yes	Yes	No	No	Yes	No	No	Yes	Yes	7/11
Santen 2002[46]	Yes	Yes	No	No	No	No	Yes	Yes	Yes	Yes	Yes	7/11
Schachter 2003[47]	Yes	Yes	Yes	Yes	No	No	Yes	Yes	Yes	Yes	Yes	9/11
Valim 2003[48]	Yes	Yes	No	Yes	No	No	Yes	Yes	No	Yes	Yes	7/11
Sencan 2004[49]	Yes	Yes	No	Yes	No	No	No	Yes	Yes	Yes	Yes	7/11
Valkeinen 2004[50]	Yes	Yes	No	Yes	No	No	No	Yes	Yes	No	Yes	6/11
Gusi 2006[51]	Yes	Yes	No	Yes	No	No	No	Yes	No	Yes	Yes	6/11
Munguia-Izquierdo 2007[52]	Yes	Yes	No	Yes	No	No	No	No	No	No	Yes	4/11
Rooks 2007[53]	No	Yes	Yes	Yes	No	No	No	No	Yes	Yes	Yes	6/11
Tomás-Carús 2007[54]	Yes	Yes	No	Yes	No	No	No	Yes	Yes	Yes	Yes	7/11
Alentorn-Geli 2008[55]	Yes	Yes	No	Yes	No	No	Yes	Yes	No	Yes	Yes	7/11
Bircan 2008[56]	Yes	Yes	No	Yes	No	No	No	Yes	Yes	Yes	Yes	7/11
Bresan 2008[57]	No	Yes	No	Yes	No	No	No	Yes	Yes	No	Yes	5/11
Evcik 2008[58]	Yes	Yes	No	No	No	No	No	Yes	No	Yes	Yes	5/11
Ide 2008[59]	Yes	Yes	Yes	Yes	No	No	Yes	No	No	Yes	Yes	7/11
Tomas-Carus2008[60]	Yes	Yes	Yes	Yes	No	No	Yes	Yes	No	Yes	Yes	8/11
Valkeinen 2008[61]	Yes	Yes	No	Yes	No	No	No	Yes	No	Yes	Yes	6/11
Altan 2009[62]	Yes	Yes	Yes	Yes	No	No	Yes	Yes	No	Yes	Yes	8/11
Carson 2010[63]	Yes	Yes	Yes	Yes	No	No	Yes	Yes	Yes	No	Yes	8/11
Kayo 2011[64]	Yes	Yes	Yes	No	No	No	Yes	No	Yes	Yes	No	6/11
Baptista 2012[65]	Yes	Yes	Yes	No	No	No	Yes	Yes	Yes	Yes	Yes	8/11
López-Rodríguez 2012[66]	Yes	Yes	No	Yes	No	No	No	No	No	Yes	Yes	5/11
Letieri 2013[67]	Yes	Yes	No	Yes	No	No	No	Yes	No	Yes	No	5/11
López-Rodríguez 2013[68]	Yes	Yes	Yes	Yes	No	No	Yes	No	Yes	Yes	Yes	8/11
Duruturk 2014[69]	Yes	Yes	Yes	Yes	No	No	No	No	No	Yes	Yes	6/11
Gavi 2014[70]	Yes	Yes	Yes	Yes	No	No	Yes	No	No	Yes	Yes	7/11
Genc 2015[71]	Yes	Yes	No	Yes	No	No	No	Yes	No	Yes	Yes	6/11
Larsson 2015[72]	Yes	Yes	No	Yes	No	No	Yes	No	No	Yes	Yes	6/11
Latorre 2015[73]	Yes	Yes	No	Yes	No	No	No	Yes	Yes	Yes	Yes	7/11
Sañudo 2015[74]	Yes	Yes	Yes	Yes	No	No	Yes	No	No	Yes	Yes	7/11

Sevimli 2015[75]	Yes	Yes	No	Yes	No	No	No	Yes	Yes	Yes	Yes	7/11
Ekici 2016[76]	Yes	Yes	Yes	Yes	No	No	Yes	No	No	Yes	Yes	7/11
Fernandes 2016[77]	Yes	Yes	Yes	Yes	No	No	Yes	Yes	Yes	Yes	Yes	9/11
Alev 2017[78]	Yes	Yes	No	Yes	No	No	Yes	Yes	No	Yes	Yes	7/11
Assumpção 2017[79]	Yes	Yes	Yes	Yes	No	No	No	No	No	Yes	Yes	6/11
Collado-Mateo 2017[80]	No	Yes	No	Yes	No	No	Yes	Yes	Yes	Yes	Yes	7/11
Toprak 2017[81]	Yes	Yes	Yes	Yes	No	No	Yes	No	No	Yes	No	6/11
Acosta-Gallego 2018[82]	Yes	Yes	Yes	Yes	No	No	No	No	No	Yes	Yes	6/11
Ernberg 2018[83]	Yes	Yes	Yes	Yes	No	No	No	No	No	Yes	Yes	6/11
Wong 2018[84]	Yes	Yes	Yes	Yes	No	No	Yes	No	Yes	Yes	Yes	8/11
Andrade 2019[85]	Yes	Yes	Yes	Yes	No	No	Yes	No	Yes	Yes	Yes	8/11
Gómez-Hernández 2019[86]	Yes	Yes	Yes	Yes	No	No	Yes	Yes	Yes	Yes	Yes	9/11
Jablochkova 2019[87]	Yes	Yes	No	Yes	No	No	No	Yes	No	Yes	Yes	6/11
Silva 2019[88]	Yes	Yes	No	Yes	No	No	Yes	Yes	Yes	Yes	Yes	8/11
Toprak 2019[89]	Yes	Yes	Yes	Yes	No	No	Yes	Yes	No	Yes	Yes	8/11
Villafaina 2019[90]	Yes	Yes	No	Yes	No	No	Yes	No	Yes	Yes	Yes	7/10
Atan 2020[91]	Yes	Yes	Yes	Yes	No	No	Yes	Yes	No	Yes	Yes	8/11
Britto 2020[92]	Yes	Yes	Yes	Yes	No	No	Yes	Yes	Yes	Yes	Yes	9/11
De Medeiros 2020[93]	Yes	Yes	Yes	Yes	No	No	Yes	Yes	Yes	Yes	Yes	9/11
Gulsen 2020[94]	Yes	Yes	Yes	Yes	No	No	No	Yes	Yes	Yes	Yes	7/11
Sarmento 2020[95]	Yes	Yes	Yes	Yes	Yes	No	Yes	No	No	Yes	Yes	7/11
De Carvalho 2020[96]	Yes	Yes	No	Yes	No	No	Yes	No	Yes	Yes	Yes	7/11
Swar 2020[97]	Yes	Yes	No	Yes	No	No	No	Yes	Yes	Yes	Yes	7/11
Arakaki 2021[98]	Yes	Yes	Yes	Yes	No	No	Yes	Yes	Yes	Yes	Yes	9/11
Garrido-Ardila 2021[99]	Yes	Yes	Yes	Yes	No	No	Yes	No	Yes	Yes	Yes	7/11
Hernando-Garijo 2021[100]	Yes	Yes	Yes	Yes	No	No	Yes	Yes	Yes	Yes	Yes	9/11
Rodríguez-Mansilla 2021[101]	Yes	Yes	Yes	No	No	No	Yes	No	Yes	Yes	Yes	7/11

Abbreviations: 1 = eligibility criteria, 2 = random allocation, 3 = concealed allocation, 4 = baseline comparability, 5 = blind subjects, 6 = blind therapists, 7 = blind assessors, 8 = adequate follow-up, 9 = intention-to-treat analysis, 10 = between-group comparisons, 11 = point estimates and variability

Fig. S.15. Funnel plot of the meta-analyses of those studies evaluating the effect of Aquatic Exercise compared to any treatment in relation to pain intensity using Visual Analogue Scale. Short term (≤ 3 months). Long term (> 3 months).

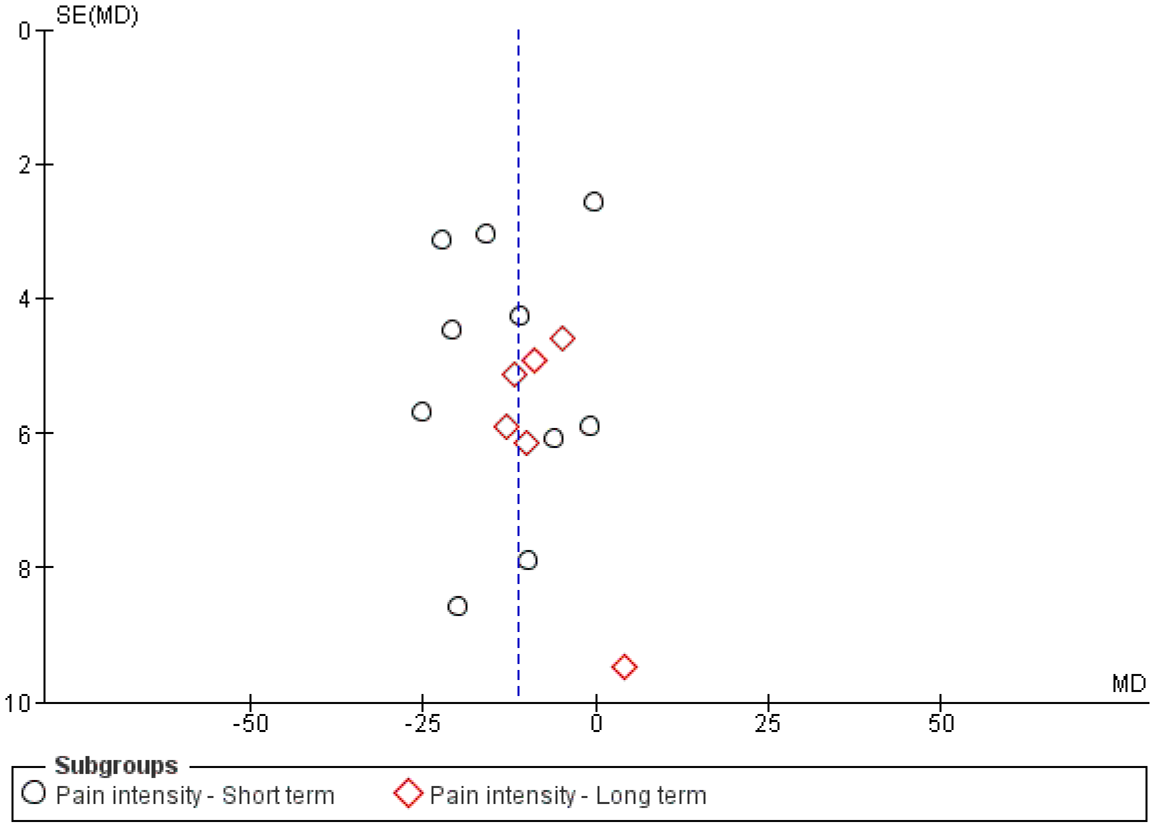

Fig. S.16. Funnel plot of the meta-analyses of those studies evaluating the effect of Aerobic Exercise compared to any treatment in relation to pain intensity using Visual Analogue Scale. Short term (≤ 3 months). Long term (> 3 months).

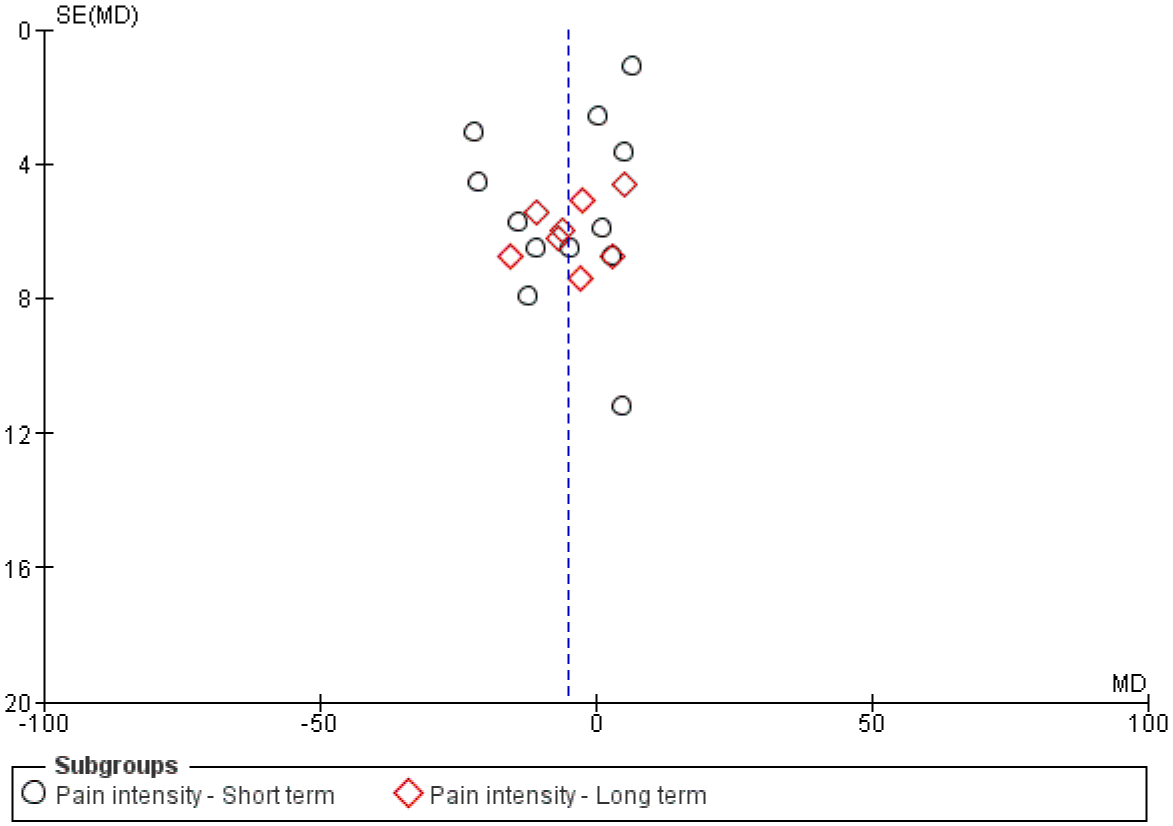

Fig. S.17. Funnel plot of the meta-analyses of those studies evaluating the effect of Resistance Training compared to any treatment in relation to pain intensity using Visual Analogue Scale. Short term (≤ 3 months). Long term (> 3 months).

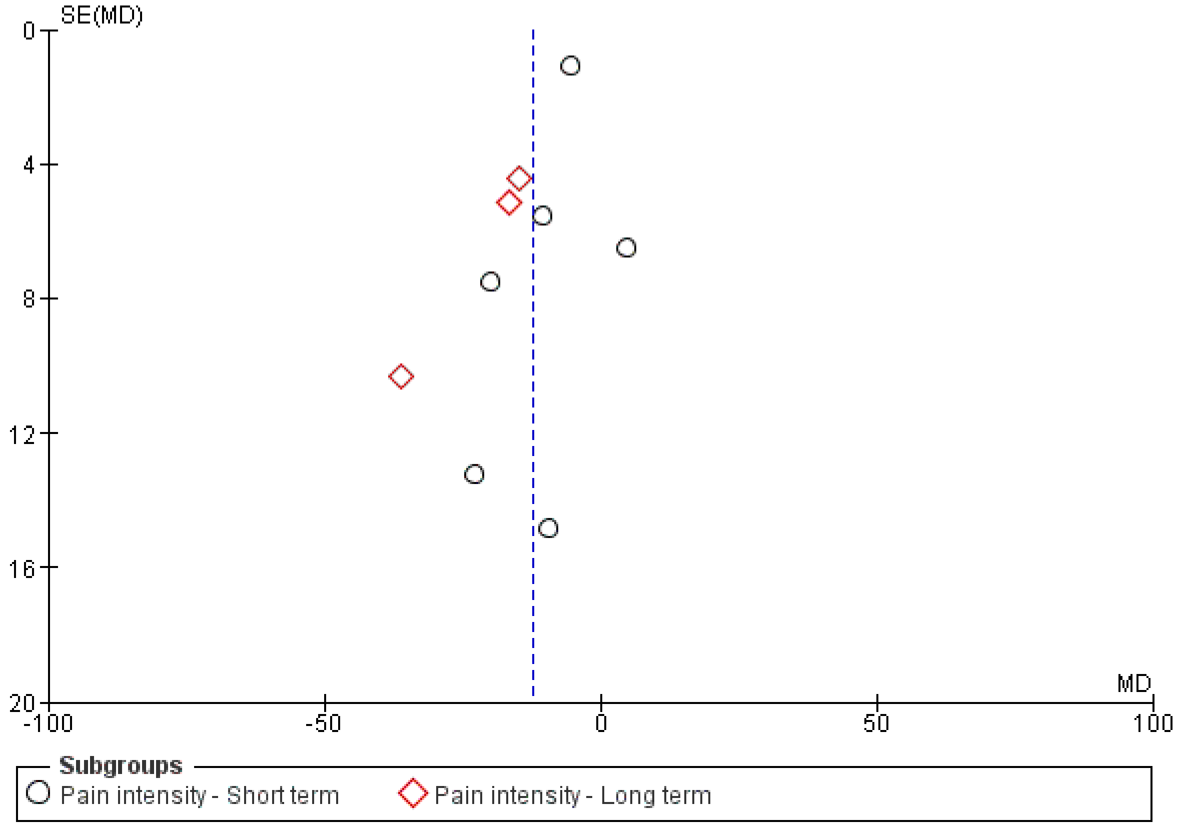

Fig. S.18. Funnel plot of the meta-analyses of those studies evaluating the effect of Flexibility compared to any treatment in relation to pain intensity using Visual Analogue Scale. Short term (≤ 3 months). Long term (> 3 months).

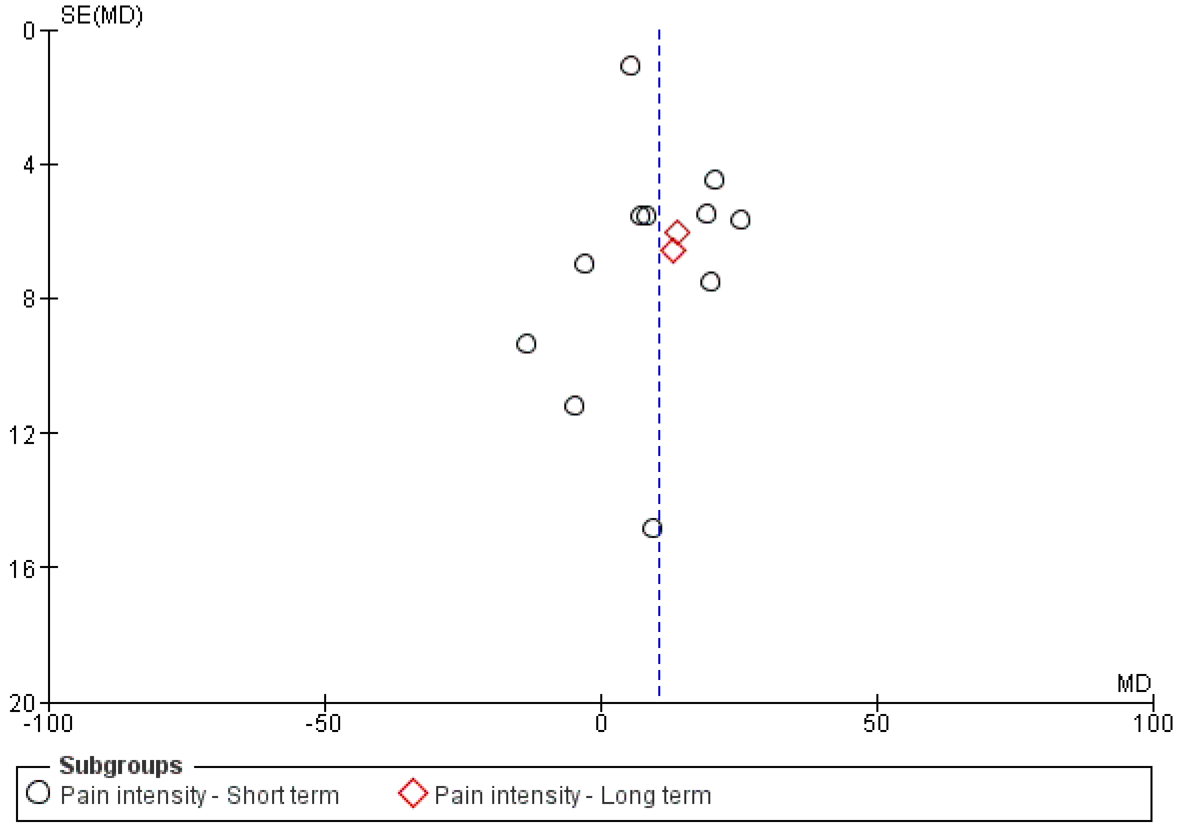

Fig. S.19. Funnel plot of the meta-analyses of those studies evaluating the effect of Mixed Exercise compared to any treatment in relation to pain intensity using Visual Analogue Scale. Short term (≤ 3 months). Long term (> 3 months).

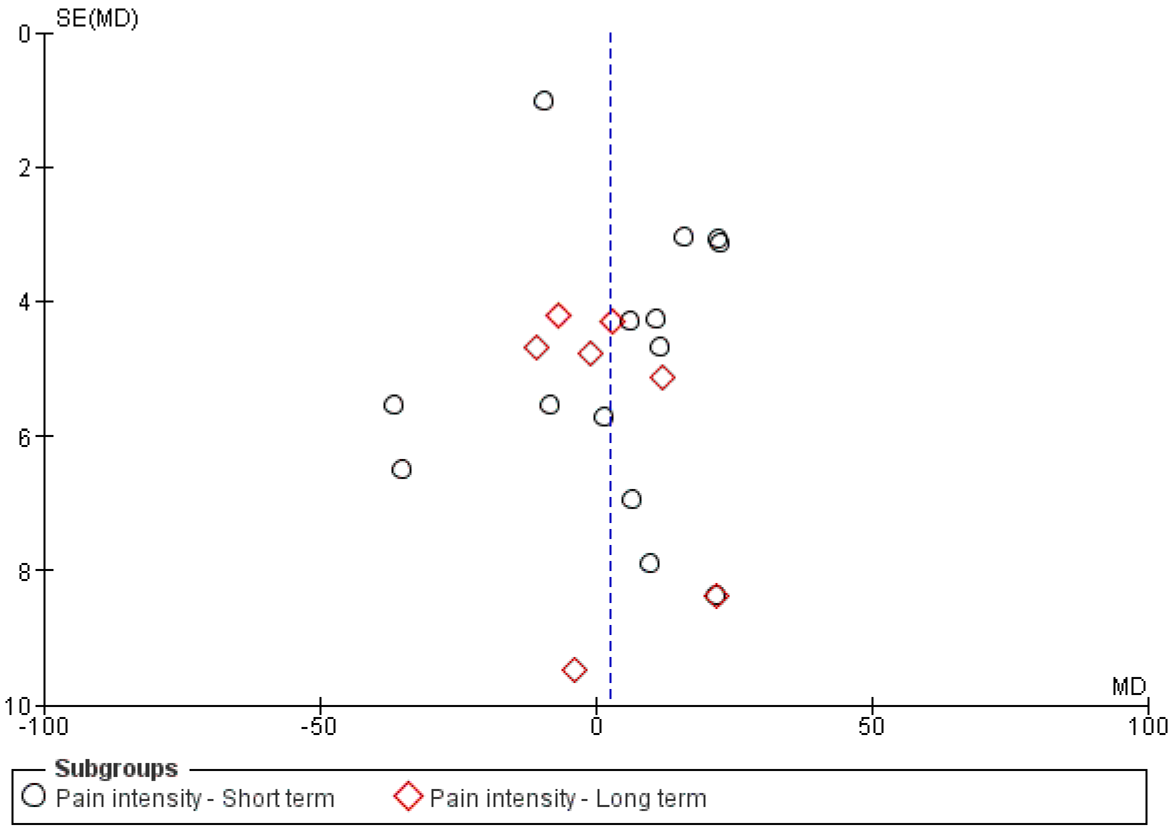

Fig. S.20. Funnel plot of the meta-analyses of those studies evaluating the effect of Core Stability compared to any treatment in relation to pain intensity using Visual Analogue Scale. Short term (≤ 3 months). Long term (> 3 months).

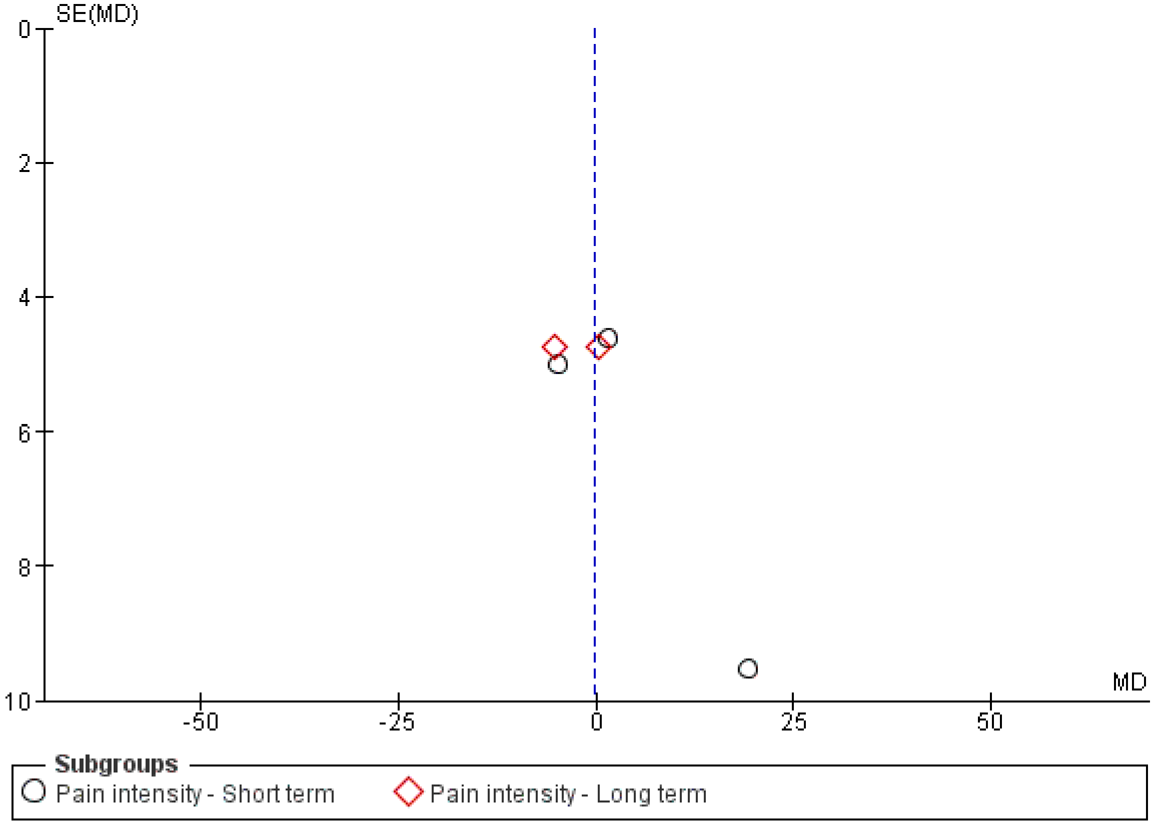

Fig. S.21. Funnel plot of the meta-analyses of those studies evaluating the effect of Dance compared to any treatment in relation to pain intensity using Visual Analogue Scale. Short term (≤ 3 months). Long term (> 3 months).

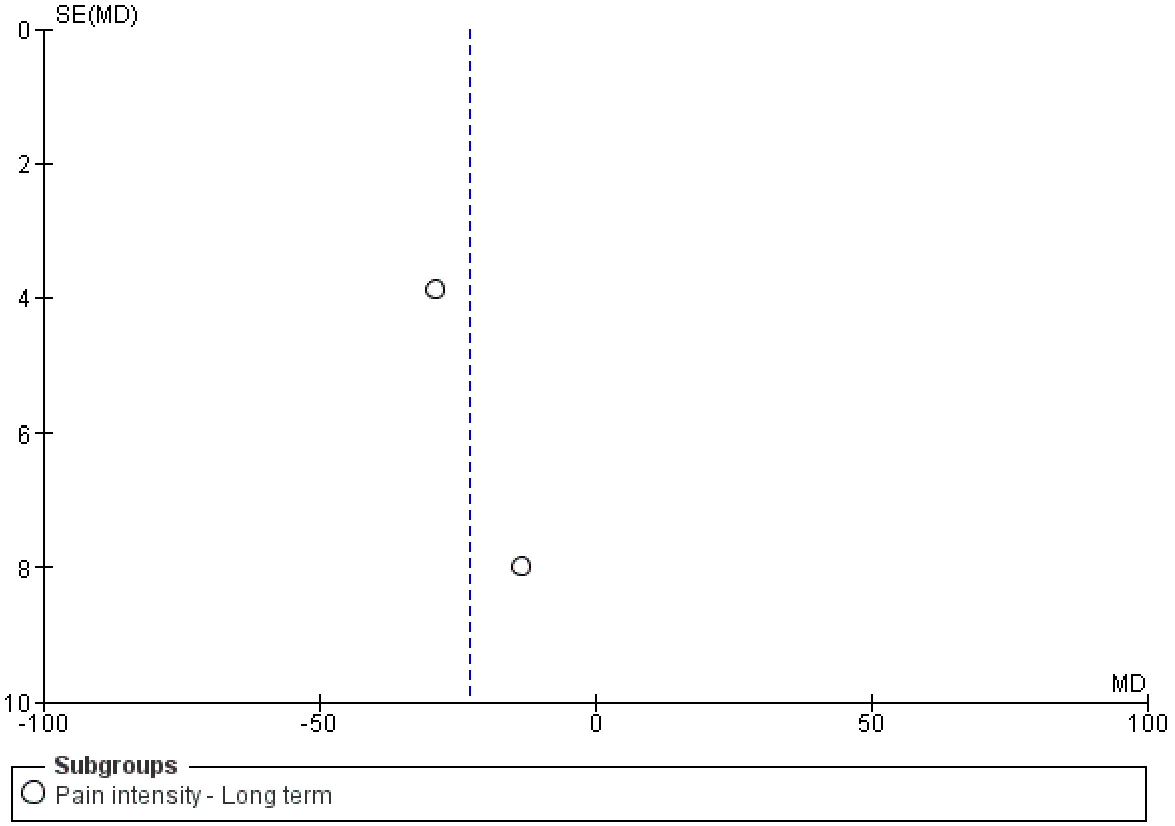

Fig. S.22. Funnel plot of the meta-analyses of those studies evaluating the effect of Functional Training compared to any treatment in relation to pain intensity using Visual Analogue Scale. Short term (≤ 3 months). Long term (> 3 months).

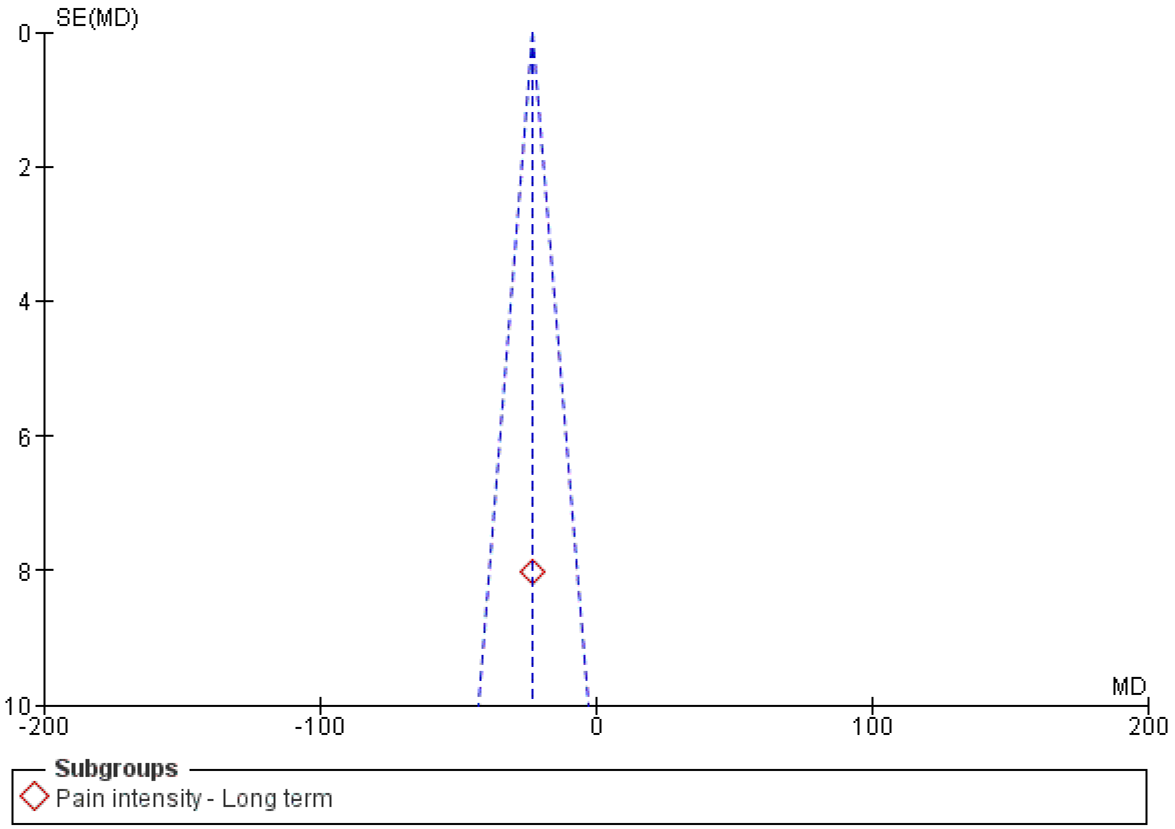

Fig. S.23. Funnel plot of the meta-analyses of those studies evaluating the effect of Pilates compared to any treatment in relation to pain intensity using Visual Analogue Scale. Short term (≤ 3 months). Long term (> 3 months).

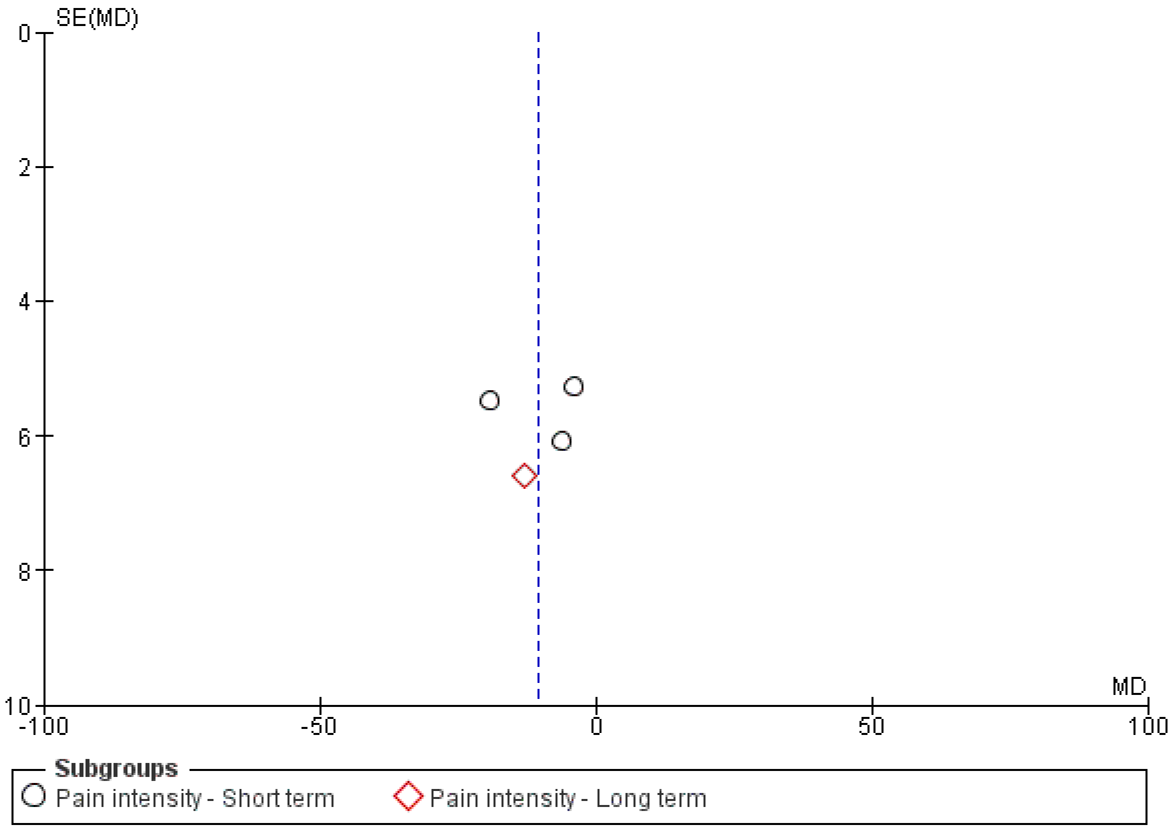

Fig. S.24. Funnel plot of the meta-analyses of those studies evaluating the effect of Balance Exercise compared to any treatment in relation to pain intensity using Visual Analogue Scale. Short term (≤ 3 months). Long term (> 3 months).

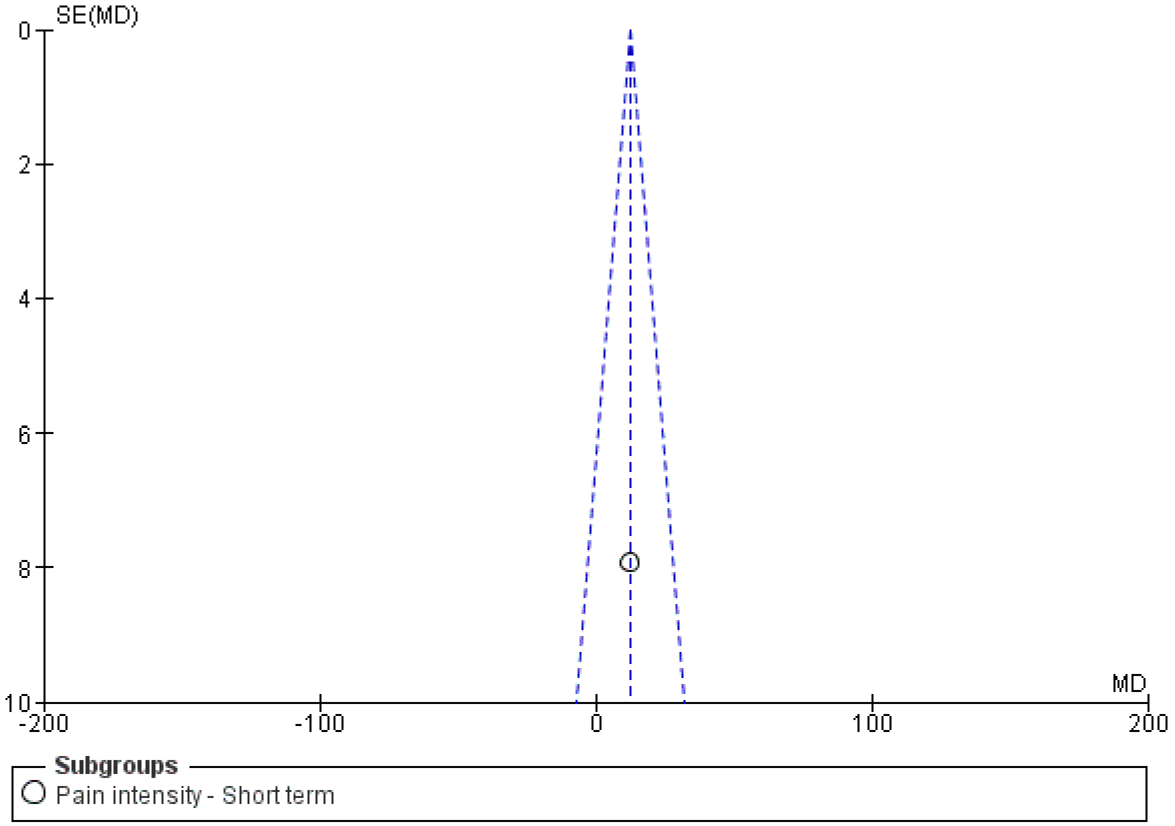

Fig. S.25. Funnel plot of the meta-analyses of those studies evaluating the effect of Qigong compared to any treatment in relation to pain intensity using Visual Analogue Scale. Short term (≤ 3 months). Long term (> 3 months).

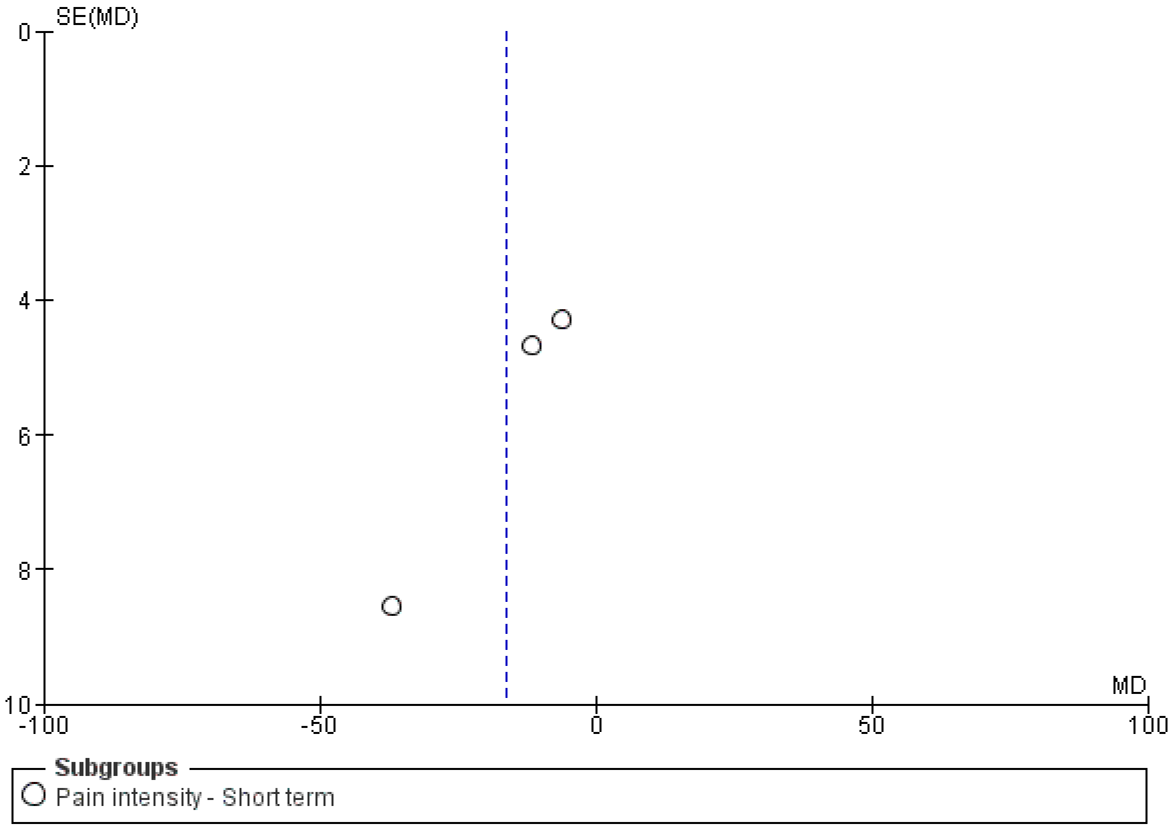

Fig. S.26. Funnel plot of the meta-analyses of those studies evaluating the effect of Tai Chi compared to any treatment in relation to pain intensity using Visual Analogue Scale. Short term (≤ 3 months). Long term (> 3 months).

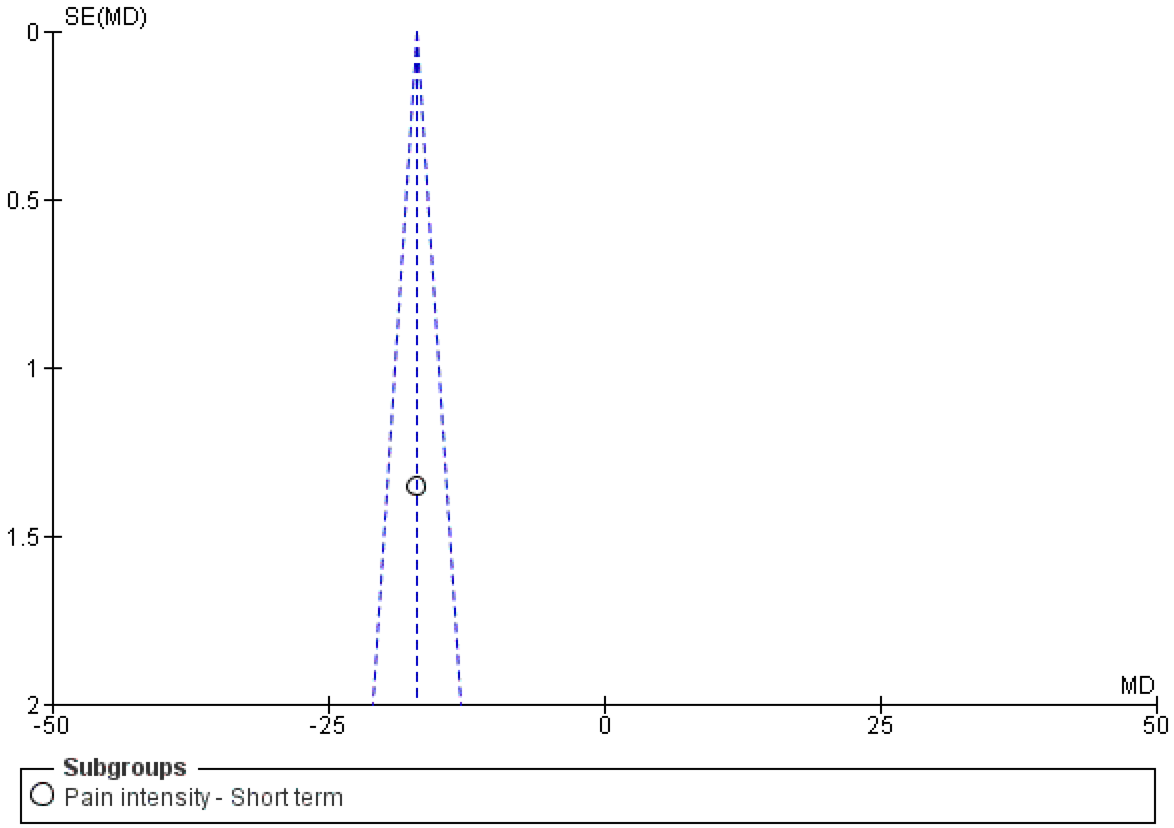

Fig. S.27. Funnel plot of the meta-analyses of those studies evaluating the effect of Virtual Reality compared to any treatment in relation to pain intensity using Visual Analogue Scale. Short term (≤ 3 months). Long term (> 3 months).

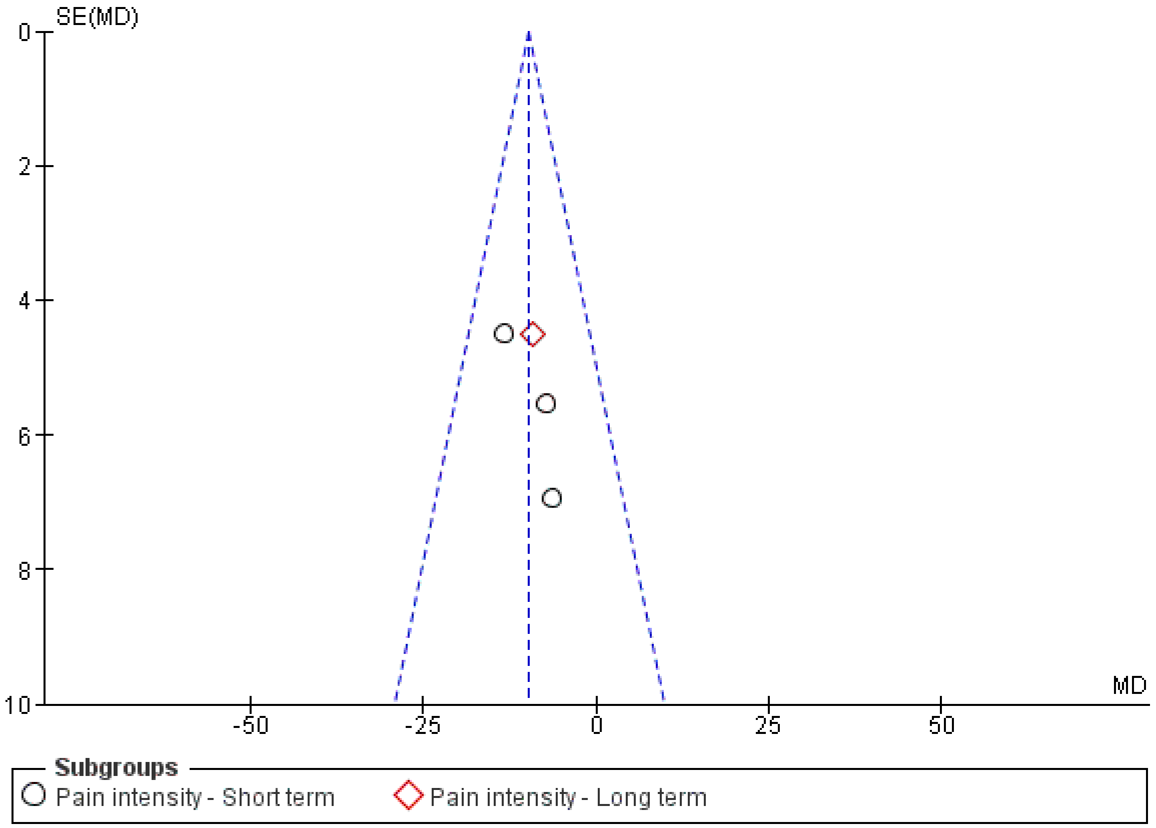

Fig. S.28. Funnel plot of the meta-analyses of those studies evaluating the effect of Yoga compared to any treatment in relation to pain intensity using Visual Analogue Scale. Short term (≤ 3 months). Long term (> 3 months).

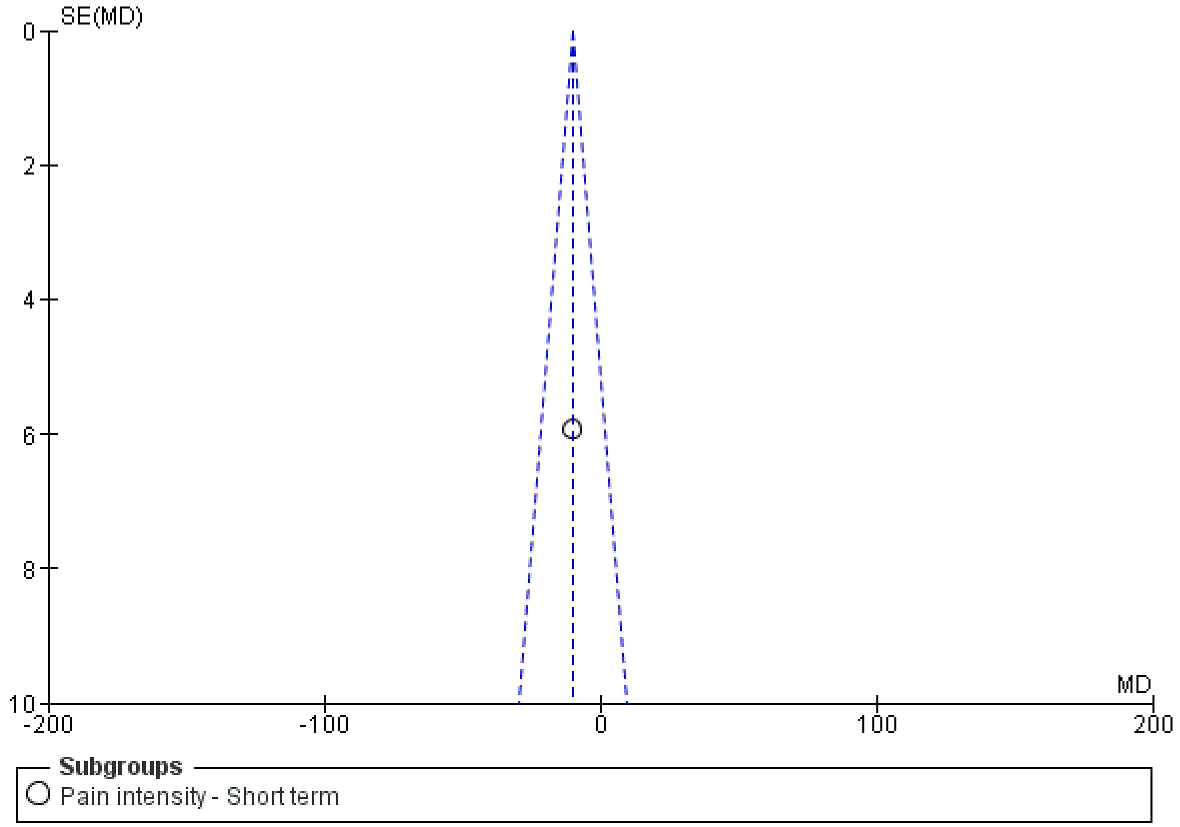

Table S.9. Judgments for each domain and overall certainty rating for each pairwise comparison (CINeMA assessment). A: short-term; B: long-term

Table S.8A. Short-term outcomes.

Comparison	Trials	Within study bias	Reporting bias	Indirectness	Imprecision	Heterogeneity	Incoherence	Confidence rating
Aerobic Exercise vs Aquatic Exercise	2	Some concerns	Low risk	No concerns	No concerns	Some concerns	No concerns	Moderate quality
Aerobic Exercise vs Balance Exercise	1	Some concerns	Low risk	No concerns	Major concerns	No concerns	Major concerns	Low quality
Aerobic Exercise vs Usual Care	2	Some concerns	Low risk	No concerns	No concerns	Some concerns	No concerns	Moderate quality
Aerobic Exercise vs Flexibility	2	Some concerns	Low risk	No concerns	Some concerns	Some concerns	No concerns	Moderate quality
Aerobic Exercise vs Mixed Exercise	2	Some concerns	Low risk	No concerns	No concerns	Major concerns	No concerns	Low quality
Aerobic Exercise vs Drugs Therapy	1	Some concerns	Low risk	No concerns	Major concerns	No concerns	No concerns	Low quality
Aerobic Exercise vs Resistance Training	1	Some concerns	Low risk	No concerns	Some concerns	Some concerns	No concerns	Moderate quality
Aerobic Exercise vs Sham	1	Some concerns	Low risk	No concerns	No concerns	Some concerns	No concerns	Moderate quality
Acupuncture vs Core Stability	1	Some concerns	Low risk	No concerns	Major concerns	No concerns	No concerns	Low quality
Acupuncture vs Usual Care	1	Some concerns	Low risk	No concerns	Major concerns	No concerns	No concerns	Low quality
Aquatic Exercise vs Usual Care	1	Some concerns	Low risk	No concerns	No concerns	No concerns	No concerns	Moderate quality
Aquatic Exercise vs Flexibility	2	Some concerns	Low risk	No concerns	No concerns	Some concerns	No concerns	Moderate quality
Aquatic Exercise vs Mixed Exercise	4	Some concerns	Low risk	No concerns	No concerns	Some concerns	No concerns	Moderate quality
Aquatic Exercise vs Pilates	1	Some concerns	Low risk	No concerns	Major concerns	No concerns	No concerns	Low quality
Core Stability vs Combined Therapy	1	Some concerns	Low risk	No concerns	Some concerns	No concerns	No concerns	Moderate quality
Core Stability vs Usual Care	1	Some concerns	Low risk	No concerns	Some concerns	Some concerns	No concerns	Moderate quality
Combined Therapy vs Mixed Exercise	2	Some concerns	Low risk	No concerns	Some concerns	Some concerns	No concerns	Moderate quality

Comparison	Trials	Within study bias	Reporting bias	Indirectness	Imprecision	Heterogeneity	Incoherence	Confidence rating
Usual Care vs Flexibility	1	Some concerns	Low risk	No concerns	Some concerns	Some concerns	No concerns	Moderate quality
Usual Care vs Mixed Exercise	3	Some concerns	Low risk	No concerns	No concerns	Some concerns	No concerns	Moderate quality
Usual Care vs Qigong	1	Some concerns	Low risk	No concerns	No concerns	Some concerns	No concerns	Moderate quality
Usual Care vs Resistance Training	1	Some concerns	Low risk	No concerns	Some concerns	No concerns	No concerns	Moderate quality
Usual Care vs Tai Chi	1	Some concerns	Low risk	No concerns	Some concerns	Some concerns	Major concerns	Low quality
Usual Care vs Virtual Reality	1	Some concerns	Low risk	No concerns	No concerns	Some concerns	No concerns	Moderate quality
Usual Care vs Yoga	1	Some concerns	Low risk	No concerns	Major concerns	No concerns	Major concerns	Low quality
Flexibility vs Mixed Exercise	1	Some concerns	Low risk	No concerns	Some concerns	Some concerns	No concerns	Moderate quality
Flexibility vs Pilates	1	Some concerns	Low risk	No concerns	Some concerns	Some concerns	No concerns	Moderate quality
Flexibility vs Resistance Training	3	Some concerns	Low risk	No concerns	Some concerns	Some concerns	No concerns	Moderate quality
Flexibility vs Virtual Reality	1	Some concerns	Low risk	No concerns	Some concerns	Some concerns	No concerns	Moderate quality
Mixed Exercise vs Qigong	1	Some concerns	Low risk	No concerns	Major concerns	No concerns	No concerns	Low quality
Mixed Exercise vs Virtual Reality	1	Some concerns	Low risk	No concerns	Major concerns	No concerns	No concerns	Low quality
Manual Therapy vs Pilates	1	Some concerns	Low risk	No concerns	Major concerns	No concerns	Major concerns	Low quality
Drugs Therapy vs Sham	1	Some concerns	Low risk	No concerns	No concerns	Some concerns	No concerns	Moderate quality
Qigong vs Sham	1	Some concerns	Low risk	No concerns	No concerns	Some concerns	No concerns	Moderate quality
Relaxation vs Resistance Training	1	Some concerns	Low risk	No concerns	Some concerns	Some concerns	Major concerns	Low quality
Acupuncture vs Aerobic Exercise	0	Some concerns	Low risk	No concerns	Major concerns	No concerns	Major concerns	Low quality
Aerobic Exercise vs Core Stability	0	Some concerns	Low risk	No concerns	Some concerns	Some concerns	Major concerns	Low quality
Aerobic Exercise vs Combined Therapy	0	Some concerns	Low risk	No concerns	Some concerns	Some concerns	Major concerns	Low quality
Aerobic Exercise vs Manual Therapy	0	Some concerns	Low risk	No concerns	Major concerns	No concerns	Major concerns	Low quality

Comparison	Trials	Within study bias	Reporting bias	Indirectness	Imprecision	Heterogeneity	Incoherence	Confidence rating
Aerobic Exercise vs Pilates	0	Some concerns	Low risk	No concerns	Some concerns	Some concerns	Major concerns	Low quality
Aerobic Exercise vs Qigong	0	Some concerns	Low risk	No concerns	Major concerns	No concerns	Major concerns	Low quality
Aerobic Exercise vs Relaxation	0	Some concerns	Low risk	No concerns	Major concerns	No concerns	Major concerns	Low quality
Aerobic Exercise vs Tai Chi	0	Some concerns	Low risk	No concerns	Major concerns	No concerns	Major concerns	Low quality
Aerobic Exercise vs Virtual Reality	0	Some concerns	Low risk	No concerns	Major concerns	No concerns	Major concerns	Low quality
Aerobic Exercise vs Yoga	0	Some concerns	Low risk	No concerns	Major concerns	No concerns	Major concerns	Low quality
Acupuncture vs Aquatic Exercise	0	Some concerns	Low risk	No concerns	Some concerns	No concerns	Major concerns	Low quality
Acupuncture vs Balance Exercise	0	Some concerns	Low risk	No concerns	Major concerns	No concerns	Major concerns	Low quality
Acupuncture vs Combined Therapy	0	Some concerns	Low risk	No concerns	Some concerns	Some concerns	Major concerns	Low quality
Acupuncture vs Flexibility	0	Some concerns	Low risk	No concerns	Major concerns	No concerns	Major concerns	Low quality
Acupuncture vs Mixed Exercise	0	Some concerns	Low risk	No concerns	Some concerns	Some concerns	Major concerns	Low quality
Acupuncture vs Manual Therapy	0	Some concerns	Low risk	No concerns	Major concerns	No concerns	Major concerns	Low quality
Acupuncture vs Drugs Therapy	0	Some concerns	Low risk	No concerns	Major concerns	No concerns	Major concerns	Low quality
Acupuncture vs Pilates	0	Some concerns	Low risk	No concerns	Some concerns	Some concerns	Major concerns	Low quality
Acupuncture vs Qigong	0	Some concerns	Low risk	No concerns	Major concerns	No concerns	Major concerns	Low quality
Acupuncture vs Relaxation	0	Some concerns	Low risk	No concerns	Major concerns	No concerns	Major concerns	Low quality
Acupuncture vs Resistance Training	0	Some concerns	Low risk	No concerns	Major concerns	No concerns	Major concerns	Low quality
Acupuncture vs Sham	0	Some concerns	Low risk	No concerns	Major concerns	No concerns	Major concerns	Low quality
Acupuncture vs Tai Chi	0	Some concerns	Low risk	No concerns	Major concerns	No concerns	Major concerns	Low quality
Acupuncture vs Virtual Reality	0	Some concerns	Low risk	No concerns	Major concerns	No concerns	Major concerns	Low quality
Acupuncture vs Yoga	0	Some concerns	Low risk	No concerns	Major concerns	No concerns	Major concerns	Low quality
Aquatic Exercise vs Balance Exercise	0	Some concerns	Low risk	No concerns	Some concerns	No concerns	Major concerns	Low quality

Comparison	Trials	Within study bias	Reporting bias	Indirectness	Imprecision	Heterogeneity	Incoherence	Confidence rating
Aquatic Exercise vs Core Stability	0	Some concerns	Low risk	No concerns	No concerns	Some concerns	Major concerns	Low quality
Aquatic Exercise vs Combined Therapy	0	Some concerns	Low risk	No concerns	Major concerns	No concerns	Major concerns	Low quality
Aquatic Exercise vs Manual Therapy	0	Some concerns	Low risk	No concerns	Major concerns	No concerns	Major concerns	Low quality
Aquatic Exercise vs Drugs Therapy	0	Some concerns	Low risk	No concerns	Major concerns	No concerns	Major concerns	Low quality
Aquatic Exercise vs Qigong	0	Some concerns	Low risk	No concerns	Some concerns	Some concerns	Major concerns	Low quality
Aquatic Exercise vs Relaxation	0	Some concerns	Low risk	No concerns	Some concerns	Some concerns	Major concerns	Low quality
Aquatic Exercise vs Resistance Training	0	Some concerns	Low risk	No concerns	Some concerns	Some concerns	Major concerns	Low quality
Aquatic Exercise vs Sham	0	Some concerns	Low risk	No concerns	No concerns	No concerns	Major concerns	Low quality
Aquatic Exercise vs Tai Chi	0	Some concerns	Low risk	No concerns	Some concerns	Some concerns	Major concerns	Low quality
Aquatic Exercise vs Virtual Reality	0	Some concerns	Low risk	No concerns	Some concerns	Some concerns	Major concerns	Low quality
Aquatic Exercise vs Yoga	0	Some concerns	Low risk	No concerns	Some concerns	Some concerns	Major concerns	Low quality
Balance Exercise vs Core Stability	0	Some concerns	Low risk	No concerns	Major concerns	No concerns	Major concerns	Low quality
Balance Exercise vs Combined Therapy	0	Some concerns	Low risk	No concerns	Some concerns	Some concerns	Major concerns	Low quality
Balance Exercise vs Usual Care	0	Some concerns	Low risk	No concerns	Major concerns	No concerns	Major concerns	Low quality
Balance Exercise vs Flexibility	0	Some concerns	Low risk	No concerns	Major concerns	No concerns	Major concerns	Low quality
Balance Exercise vs Mixed Exercise	0	Some concerns	Low risk	No concerns	Major concerns	No concerns	Major concerns	Low quality
Balance Exercise vs Manual Therapy	0	Some concerns	Low risk	No concerns	Major concerns	No concerns	Major concerns	Low quality
Balance Exercise vs Drugs Therapy	0	Some concerns	Low risk	No concerns	Major concerns	No concerns	Major concerns	Low quality
Balance Exercise vs Pilates	0	Some concerns	Low risk	No concerns	Some concerns	Some concerns	Major concerns	Low quality
Balance Exercise vs Qigong	0	Some concerns	Low risk	No concerns	Major concerns	No concerns	Major concerns	Low quality
Balance Exercise vs Relaxation	0	Some concerns	Low risk	No concerns	Major concerns	No concerns	Major concerns	Low quality

Comparison	Trials	Within study bias	Reporting bias	Indirectness	Imprecision	Heterogeneity	Incoherence	Confidence rating
Balance Exercise vs Resistance Training	0	Some concerns	Low risk	No concerns	Major concerns	No concerns	Major concerns	Low quality
Balance Exercise vs Sham	0	Some concerns	Low risk	No concerns	Major concerns	No concerns	Major concerns	Low quality
Balance Exercise vs Tai Chi	0	Some concerns	Low risk	No concerns	Major concerns	No concerns	Major concerns	Low quality
Balance Exercise vs Virtual Reality	0	Some concerns	Low risk	No concerns	Major concerns	No concerns	Major concerns	Low quality
Balance Exercise vs Yoga	0	Some concerns	Low risk	No concerns	Major concerns	No concerns	Major concerns	Low quality
Core Stability vs Flexibility	0	Some concerns	Low risk	No concerns	Major concerns	No concerns	Major concerns	Low quality
Core Stability vs Mixed Exercise	0	Some concerns	Low risk	No concerns	Some concerns	Some concerns	Major concerns	Low quality
Core Stability vs Manual Therapy	0	Some concerns	Low risk	No concerns	Major concerns	No concerns	Major concerns	Low quality
Core Stability vs Drugs Therapy	0	Some concerns	Low risk	No concerns	Major concerns	No concerns	Major concerns	Low quality
Core Stability vs Pilates	0	Some concerns	Low risk	No concerns	Some concerns	Some concerns	Major concerns	Low quality
Core Stability vs Qigong	0	Some concerns	Low risk	No concerns	Some concerns	Some concerns	Major concerns	Low quality
Core Stability vs Relaxation	0	Some concerns	Low risk	No concerns	Major concerns	No concerns	Major concerns	Low quality
Core Stability vs Resistance Training	0	Some concerns	Low risk	No concerns	Major concerns	No concerns	Major concerns	Low quality
Core Stability vs Sham	0	Some concerns	Low risk	No concerns	Some concerns	Some concerns	Major concerns	Low quality
Core Stability vs Tai Chi	0	Some concerns	Low risk	No concerns	Major concerns	No concerns	Major concerns	Low quality
Core Stability vs VR	0	Some concerns	Low risk	No concerns	Some concerns	Some concerns	Major concerns	Low quality
Core Stability vs Yoga	0	Some concerns	Low risk	No concerns	Major concerns	No concerns	Major concerns	Low quality
Combined Therapy vs Usual Care	0	Some concerns	Low risk	No concerns	No concerns	Some concerns	Major concerns	Low quality
Combined Therapy vs Flexibility	0	Some concerns	Low risk	No concerns	Some concerns	Some concerns	Major concerns	Low quality
Combined Therapy vs Manual Therapy	0	Some concerns	Low risk	No concerns	Major concerns	No concerns	Major concerns	Low quality
Combined Therapy vs Drugs Therapy	0	Some concerns	Low risk	No concerns	Major concerns	No concerns	Major concerns	Low quality
Combined Therapy vs Pilates	0	Some concerns	Low risk	No concerns	Major concerns	No concerns	Major concerns	Low quality

Comparison	Trials	Within study bias	Reporting bias	Indirectness	Imprecision	Heterogeneity	Incoherence	Confidence rating
Combined Therapy vs Qigong	0	Some concerns	Low risk	No concerns	Major concerns	No concerns	Major concerns	Low quality
Combined Therapy vs Relaxation	0	Some concerns	Low risk	No concerns	Some concerns	Some concerns	Major concerns	Low quality
Combined Therapy vs Resistance Training	0	Some concerns	Low risk	No concerns	Some concerns	Some concerns	Major concerns	Low quality
Combined Therapy vs Sham	0	Some concerns	Low risk	No concerns	No concerns	Some concerns	Major concerns	Low quality
Combined Therapy vs Tai Chi	0	Some concerns	Low risk	No concerns	Major concerns	No concerns	Major concerns	Low quality
Combined Therapy vs Virtual Reality	0	Some concerns	Low risk	No concerns	Some concerns	Some concerns	Major concerns	Low quality
Combined Therapy vs Yoga	0	Some concerns	Low risk	No concerns	Some concerns	Some concerns	Major concerns	Low quality
Usual Care vs Manual Therapy	0	Some concerns	Low risk	No concerns	Some concerns	Some concerns	Major concerns	Low quality
Usual Care vs Drugs Therapy	0	Some concerns	Low risk	No concerns	Some concerns	Some concerns	Major concerns	Low quality
Usual Care vs Pilates	0	Some concerns	Low risk	No concerns	No concerns	Some concerns	Major concerns	Low quality
Usual Care vs Relaxation	0	Some concerns	Low risk	No concerns	Major concerns	No concerns	Major concerns	Low quality
Usual Care vs Sham	0	Some concerns	Low risk	No concerns	Some concerns	Some concerns	Major concerns	Low quality
Flexibility vs Manual Therapy	0	Some concerns	Low risk	No concerns	Major concerns	No concerns	Major concerns	Low quality
Flexibility vs Drugs Therapy	0	Some concerns	Low risk	No concerns	Major concerns	No concerns	Major concerns	Low quality
Flexibility vs Qigong	0	Some concerns	Low risk	No concerns	Some concerns	Some concerns	Major concerns	Low quality
Flexibility vs Relaxation	0	Some concerns	Low risk	No concerns	Major concerns	No concerns	Major concerns	Low quality
Flexibility vs Sham	0	Some concerns	Low risk	No concerns	Some concerns	No concerns	Major concerns	Low quality
Flexibility vs Tai Chi	0	Some concerns	Low risk	No concerns	Major concerns	No concerns	Major concerns	Low quality
Flexibility vs Yoga	0	Some concerns	Low risk	No concerns	Major concerns	No concerns	Major concerns	Low quality
Mixed Exercise vs Manual Therapy	0	Some concerns	Low risk	No concerns	Major concerns	No concerns	Major concerns	Low quality
Mixed Exercise vs Drugs Therapy	0	Some concerns	Low risk	No concerns	Major concerns	No concerns	Major concerns	Low quality
Mixed Exercise vs Pilates	0	Some concerns	Low risk	No concerns	Some concerns	Some concerns	Major concerns	Low quality
Mixed Exercise vs Relaxation	0	Some concerns	Low risk	No concerns	Major concerns	No concerns	Major concerns	Low quality

Comparison	Trials	Within study bias	Reporting bias	Indirectness	Imprecision	Heterogeneity	Incoherence	Confidence rating
Mixed Exercise vs Resistance Training	0	Some concerns	Low risk	No concerns	Some concerns	Some concerns	Major concerns	Low quality
Mixed Exercise vs Sham	0	Some concerns	Low risk	No concerns	No concerns	Some concerns	Major concerns	Low quality
Mixed Exercise vs Tai Chi	0	Some concerns	Low risk	No concerns	Major concerns	No concerns	Major concerns	Low quality
Mixed Exercise vs Yoga	0	Some concerns	Low risk	No concerns	Major concerns	No concerns	Major concerns	Low quality
Manual Therapy vs Drugs Therapy	0	Some concerns	Low risk	No concerns	Major concerns	No concerns	Major concerns	Low quality
Manual Therapy vs Qigong	0	Some concerns	Low risk	No concerns	Major concerns	No concerns	Major concerns	Low quality
Manual Therapy vs Relaxation	0	Some concerns	Low risk	No concerns	Major concerns	No concerns	Major concerns	Low quality
Manual Therapy vs Resistance Training	0	Some concerns	Low risk	No concerns	Major concerns	No concerns	Major concerns	Low quality
Manual Therapy vs Sham	0	Some concerns	Low risk	No concerns	Some concerns	No concerns	Major concerns	Low quality
Manual Therapy vs Tai Chi	0	Some concerns	Low risk	No concerns	Major concerns	No concerns	Major concerns	Low quality
Manual Therapy vs VR	0	Some concerns	Low risk	No concerns	Major concerns	No concerns	Major concerns	Low quality
Manual Therapy vs Yoga	0	Some concerns	Low risk	No concerns	Major concerns	No concerns	Major concerns	Low quality
Drugs Therapy vs Pilates	0	Some concerns	Low risk	No concerns	Major concerns	No concerns	Major concerns	Low quality
Drugs Therapy vs Qigong	0	Some concerns	Low risk	No concerns	Major concerns	No concerns	Major concerns	Low quality
Drugs Therapy vs Relaxation	0	Some concerns	Low risk	No concerns	Major concerns	No concerns	Major concerns	Low quality
Drugs Therapy vs Resistance Training	0	Some concerns	Low risk	No concerns	Major concerns	No concerns	Major concerns	Low quality
Drugs Therapy vs Tai Chi	0	Some concerns	Low risk	No concerns	Major concerns	No concerns	Major concerns	Low quality
Drugs Therapy vs Virtual Reality	0	Some concerns	Low risk	No concerns	Major concerns	No concerns	Major concerns	Low quality
Drugs Therapy vs Yoga	0	Some concerns	Low risk	No concerns	Major concerns	No concerns	Major concerns	Low quality
Pilates vs Qigong	0	Some concerns	Low risk	No concerns	Major concerns	No concerns	Major concerns	Low quality
Pilates vs Relaxation	0	Some concerns	Low risk	No concerns	Some concerns	Some concerns	Major concerns	Low quality

Comparison	Trials	Within study bias	Reporting bias	Indirectness	Imprecision	Heterogeneity	Incoherence	Confidence rating
Pilates vs Resistance Training	0	Some concerns	Low risk	No concerns	Some concerns	Some concerns	Major concerns	Low quality
Pilates vs Sham	0	Some concerns	Low risk	No concerns	No concerns	Some concerns	Major concerns	Low quality
Pilates vs Tai Chi	0	Some concerns	Low risk	No concerns	Major concerns	No concerns	Major concerns	Low quality
Pilates vs Virtual Reality	0	Some concerns	Low risk	No concerns	Major concerns	No concerns	Major concerns	Low quality
Pilates vs Yoga	0	Some concerns	Low risk	No concerns	Major concerns	No concerns	Major concerns	Low quality
Qigong vs Relaxation	0	Some concerns	Low risk	No concerns	Major concerns	No concerns	Major concerns	Low quality
Qigong vs Resistance Training	0	Some concerns	Low risk	No concerns	Major concerns	No concerns	Major concerns	Low quality
Qigong vs Tai Chi	0	Some concerns	Low risk	No concerns	Major concerns	No concerns	Major concerns	Low quality
Qigong vs VR	0	Some concerns	Low risk	No concerns	Major concerns	No concerns	Major concerns	Low quality
Qigong vs Yoga	0	Some concerns	Low risk	No concerns	Major concerns	No concerns	Major concerns	Low quality
Relaxation vs Sham	0	Some concerns	Low risk	No concerns	Major concerns	No concerns	Major concerns	Low quality
Relaxation vs Tai Chi	0	Some concerns	Low risk	No concerns	Major concerns	No concerns	Major concerns	Low quality
Relaxation vs Virtual Reality	0	Some concerns	Low risk	No concerns	Major concerns	No concerns	Major concerns	Low quality
Relaxation vs Yoga	0	Some concerns	Low risk	No concerns	Major concerns	No concerns	Major concerns	Low quality
Sham vs Resistance Training	0	Some concerns	Low risk	No concerns	No concerns	Some concerns	Major concerns	Low quality
Resistance Training vs Tai Chi	0	Some concerns	Low risk	No concerns	Major concerns	No concerns	Major concerns	Low quality
Resistance Training vs VR	0	Some concerns	Low risk	No concerns	Major concerns	No concerns	Major concerns	Low quality
Resistance Training vs Yoga	0	Some concerns	Low risk	No concerns	Major concerns	No concerns	Major concerns	Low quality
Sham vs Tai Chi	0	Some concerns	Low risk	No concerns	Some concerns	No concerns	Major concerns	Low quality
Sham vs Virtual Reality	0	Some concerns	Low risk	No concerns	No concerns	Some concerns	Major concerns	Low quality
Sham vs Yoga	0	Some concerns	Low risk	No concerns	Some concerns	Some concerns	Major concerns	Low quality
Tai Chi vs Virtual Reality	0	Some concerns	Low risk	No concerns	Major concerns	No concerns	Major concerns	Low quality
Tai Chi vs Yoga	0	Some concerns	Low risk	No concerns	Major concerns	No concerns	Major concerns	Low quality
Virtual Reality vs Yoga	0	Some concerns	Low risk	No concerns	Major concerns	No concerns	Major concerns	Low quality

Table S.8B. Long-term outcomes.

Comparison	Trials	Within study bias	Reporting bias	Indirectness	Imprecision	Heterogeneity	Incoherence	Confidence rating
Aerobic Exercise vs Aquatic Exercise	1	Some concerns	Low risk	No concerns	No concerns	No concerns	No concerns	Moderate quality
Aerobic Exercise vs Usual Care	4	No concerns	Low risk	No concerns	No concerns	No concerns	No concerns	High quality
Aerobic Exercise vs Flexibility	1	Some concerns	Low risk	No concerns	Some concerns	No concerns	No concerns	Moderate quality
Aerobic Exercise vs Drugs Therapy	1	Some concerns	Low risk	No concerns	No concerns	No concerns	No concerns	Moderate quality
Aerobic Exercise vs Sham	1	Some concerns	Low risk	No concerns	No concerns	Some concerns	No concerns	Moderate quality
Acupuncture vs Core Stability	1	Some concerns	Low risk	No concerns	No concerns	No concerns	No concerns	Moderate quality
Acupuncture vs Usual Care	1	Some concerns	Low risk	No concerns	No concerns	Some concerns	No concerns	Moderate quality
Aquatic Exercise vs Usual Care	4	Some concerns	Low risk	No concerns	No concerns	No concerns	No concerns	Moderate quality
Aquatic Exercise vs Mixed Exercise	1	Some concerns	Low risk	No concerns	Some concerns	Some concerns	No concerns	Moderate quality
Core Stability vs Usual Care	1	Some concerns	Low risk	No concerns	No concerns	Some concerns	No concerns	Moderate quality
Combined Therapy vs Education	2	Some concerns	Low risk	No concerns	No concerns	No concerns	No concerns	Moderate quality
Combined Therapy vs Mixed Exercise	3	Some concerns	Low risk	No concerns	No concerns	No concerns	No concerns	Moderate quality
Usual Care vs Dance	2	Some concerns	Low risk	No concerns	No concerns	No concerns	No concerns	Moderate quality
Usual Care vs Functional Training	1	Some concerns	Low risk	No concerns	No concerns	No concerns	No concerns	Moderate quality
Usual Care vs Resistance Training	1	Some concerns	Low risk	No concerns	No concerns	No concerns	No concerns	Moderate quality
Usual Care vs Virtual Reality	1	Some concerns	Low risk	No concerns	No concerns	Some concerns	No concerns	Moderate quality

Comparison	Trials	Within study bias	Reporting bias	Indirectness	Imprecision	Heterogeneity	Incoherence	Confidence rating
Education vs Mixed Exercise	2	Some concerns	Low risk	No concerns	No concerns	No concerns	No concerns	Moderate quality
Flexibility vs Pilates	1	Some concerns	Low risk	No concerns	No concerns	Some concerns	No concerns	Moderate quality
Drugs Therapy vs Sham	1	Some concerns	Low risk	No concerns	Some concerns	No concerns	No concerns	Moderate quality
RT vs Resistance Training	2	Some concerns	Low risk	No concerns	No concerns	No concerns	No concerns	Moderate quality
Acupuncture vs Aerobic Exercise	0	Some concerns	Low risk	No concerns	No concerns	No concerns	No concerns	Moderate quality
Aerobic Exercise vs Core Stability	0	Some concerns	Low risk	No concerns	No concerns	No concerns	No concerns	Moderate quality
Aerobic Exercise vs Combined Therapy	0	Some concerns	Low risk	No concerns	Some concerns	No concerns	No concerns	Moderate quality
Aerobic Exercise vs Dance	0	Some concerns	Low risk	No concerns	No concerns	No concerns	No concerns	Moderate quality
Aerobic Exercise vs Education	0	Some concerns	Low risk	No concerns	Major concerns	No concerns	No concerns	Low quality
Aerobic Exercise vs Functional Training	0	Some concerns	Low risk	No concerns	Some concerns	No concerns	No concerns	Moderate quality
Aerobic Exercise vs Mixed Exercise	0	Some concerns	Low risk	No concerns	Some concerns	No concerns	No concerns	Moderate quality
Aerobic Exercise vs Pilates	0	Some concerns	Low risk	No concerns	Major concerns	No concerns	No concerns	Low quality
Aerobic Exercise vs Relaxation	0	Some concerns	Low risk	No concerns	Some concerns	No concerns	No concerns	Moderate quality
Aerobic Exercise vs Resistance Training	0	Some concerns	Low risk	No concerns	No concerns	No concerns	No concerns	Moderate quality
Aerobic Exercise vs Virtual Reality	0	Some concerns	Low risk	No concerns	No concerns	No concerns	No concerns	Moderate quality
Acupuncture vs Aquatic Exercise	0	Some concerns	Low risk	No concerns	Some concerns	No concerns	No concerns	Moderate quality
Acupuncture vs Combined Therapy	0	Some concerns	Low risk	No concerns	Some concerns	No concerns	No concerns	Moderate quality
Acupuncture vs Dance	0	Some concerns	Low risk	No concerns	No concerns	No concerns	No concerns	Moderate quality
Acupuncture vs Education	0	Some concerns	Low risk	No concerns	Major concerns	No concerns	No concerns	Low quality
Acupuncture vs Flexibility	0	Some concerns	Low risk	No concerns	Some concerns	No concerns	No concerns	Moderate quality
Acupuncture vs Functional Training	0	Some concerns	Low risk	No concerns	Some concerns	No concerns	No concerns	Moderate quality

Comparison	Trials	Within study bias	Reporting bias	Indirectness	Imprecision	Heterogeneity	Incoherence	Confidence rating
Acupuncture vs Mixed Exercise	0	Some concerns	Low risk	No concerns	Some concerns	No concerns	No concerns	Moderate quality
Acupuncture vs Drugs Therapy	0	Some concerns	Low risk	No concerns	Some concerns	Some concerns	No concerns	Moderate quality
Acupuncture vs Pilates	0	Some concerns	Low risk	No concerns	Major concerns	No concerns	No concerns	Low quality
Acupuncture vs Relaxation	0	Some concerns	Low risk	No concerns	Some concerns	No concerns	No concerns	Moderate quality
Acupuncture vs Resistance Training	0	Some concerns	Low risk	No concerns	No concerns	No concerns	No concerns	Moderate quality
Acupuncture vs Sham	0	Some concerns	Low risk	No concerns	Some concerns	No concerns	No concerns	Moderate quality
Acupuncture vs Virtual Reality	0	Some concerns	Low risk	No concerns	Some concerns	No concerns	No concerns	Moderate quality
Aquatic Exercise vs Core Stability	0	Some concerns	Low risk	No concerns	Some concerns	No concerns	No concerns	Moderate quality
Aquatic Exercise vs Combined Therapy	0	Some concerns	Low risk	No concerns	Some concerns	Some concerns	No concerns	Moderate quality
Aquatic Exercise vs Dance	0	Some concerns	Low risk	No concerns	No concerns	No concerns	No concerns	Moderate quality
Aquatic Exercise vs Education	0	Some concerns	Low risk	No concerns	Major concerns	No concerns	No concerns	Low quality
Aquatic Exercise vs Flexibility	0	Some concerns	Low risk	No concerns	No concerns	No concerns	No concerns	Moderate quality
Aquatic Exercise vs Functional Training	0	Some concerns	Low risk	No concerns	Some concerns	No concerns	No concerns	Moderate quality
Aquatic Exercise vs Drugs Therapy	0	Some concerns	Low risk	No concerns	Some concerns	No concerns	No concerns	Moderate quality
Aquatic Exercise vs Pilates	0	Some concerns	Low risk	No concerns	Major concerns	No concerns	No concerns	Low quality
Aquatic Exercise vs Relaxation	0	Some concerns	Low risk	No concerns	Some concerns	Some concerns	No concerns	Moderate quality
Aquatic Exercise vs Resistance Training	0	Some concerns	Low risk	No concerns	No concerns	No concerns	No concerns	Moderate quality
Aquatic Exercise vs Sham	0	Some concerns	Low risk	No concerns	No concerns	No concerns	No concerns	Moderate quality
Aquatic Exercise vs Virtual Reality	0	Some concerns	Low risk	No concerns	No concerns	No concerns	No concerns	Moderate quality
Core Stability vs Combined Therapy	0	Some concerns	Low risk	No concerns	Some concerns	No concerns	No concerns	Moderate quality
Core Stability vs Dance	0	Some concerns	Low risk	No concerns	No concerns	No concerns	No concerns	Moderate quality

Comparison	Trials	Within study bias	Reporting bias	Indirectness	Imprecision	Heterogeneity	Incoherence	Confidence rating
Core Stability vs Education	0	Some concerns	Low risk	No concerns	Major concerns	No concerns	No concerns	Low quality
Core Stability vs Flexibility	0	Some concerns	Low risk	No concerns	Some concerns	No concerns	No concerns	Moderate quality
Core Stability vs Functional Training	0	Some concerns	Low risk	No concerns	Some concerns	No concerns	No concerns	Moderate quality
Core Stability vs Mixed Exercise	0	Some concerns	Low risk	No concerns	Some concerns	No concerns	No concerns	Moderate quality
Core Stability vs Drugs Therapy	0	Some concerns	Low risk	No concerns	Some concerns	Some concerns	No concerns	Moderate quality
Core Stability vs Pilates	0	Some concerns	Low risk	No concerns	Major concerns	No concerns	No concerns	Low quality
Core Stability vs Relaxation	0	Some concerns	Low risk	No concerns	Some concerns	No concerns	No concerns	Moderate quality
Core Stability vs Resistance Training	0	Some concerns	Low risk	No concerns	No concerns	No concerns	No concerns	Moderate quality
Core Stability vs Sham	0	Some concerns	Low risk	No concerns	Some concerns	No concerns	No concerns	Moderate quality
Core Stability vs Virtual Reality	0	Some concerns	Low risk	No concerns	Some concerns	No concerns	No concerns	Moderate quality
Combined Therapy vs Usual Care	0	Some concerns	Low risk	No concerns	Some concerns	No concerns	No concerns	Moderate quality
Combined Therapy vs Dance	0	Some concerns	Low risk	No concerns	Some concerns	Some concerns	No concerns	Moderate quality
Combined Therapy vs Flexibility	0	Some concerns	Low risk	No concerns	Some concerns	No concerns	No concerns	Moderate quality
Combined Therapy vs Functional Training	0	Some concerns	Low risk	No concerns	Major concerns	No concerns	No concerns	Low quality
Combined Therapy vs Drugs Therapy	0	Some concerns	Low risk	No concerns	Some concerns	No concerns	No concerns	Moderate quality
Combined Therapy vs Pilates	0	Some concerns	Low risk	No concerns	Major concerns	No concerns	No concerns	Low quality
Combined Therapy vs Relaxation	0	Some concerns	Low risk	No concerns	Major concerns	No concerns	No concerns	Low quality
Combined Therapy vs Resistance Training	0	Some concerns	Low risk	No concerns	Some concerns	No concerns	No concerns	Moderate quality
Combined Therapy vs Sham	0	Some concerns	Low risk	No concerns	Some concerns	No concerns	No concerns	Moderate quality
Combined Therapy vs Virtual Reality	0	Some concerns	Low risk	No concerns	Some concerns	Some concerns	No concerns	Moderate quality
Usual Care vs Education	0	Some concerns	Low risk	No concerns	Some concerns	Some concerns	No concerns	Moderate quality

Comparison	Trials	Within study bias	Reporting bias	Indirectness	Imprecision	Heterogeneity	Incoherence	Confidence rating
Usual Care vs Flexibility	0	Some concerns	Low risk	No concerns	Some concerns	No concerns	No concerns	Moderate quality
Usual Care vs Mixed Exercise	0	Major concerns	Low risk	No concerns	Some concerns	No concerns	No concerns	Low quality
Usual Care vs Drugs Therapy	0	Some concerns	Low risk	No concerns	Some concerns	No concerns	No concerns	Moderate quality
Usual Care vs Pilates	0	Some concerns	Low risk	No concerns	Some concerns	No concerns	No concerns	Moderate quality
Usual Care vs Relaxation	0	Some concerns	Low risk	No concerns	Some concerns	No concerns	No concerns	Moderate quality
Usual Care vs Sham	0	Some concerns	Low risk	No concerns	Some concerns	No concerns	No concerns	Moderate quality
Dance vs Education	0	Some concerns	Low risk	No concerns	Some concerns	No concerns	No concerns	Moderate quality
Dance vs Flexibility	0	Some concerns	Low risk	No concerns	No concerns	No concerns	No concerns	Moderate quality
Dance vs Functional Training	0	Some concerns	Low risk	No concerns	Some concerns	Some concerns	No concerns	Moderate quality
Dance vs Mixed Exercise	0	Some concerns	Low risk	No concerns	Some concerns	No concerns	No concerns	Moderate quality
Dance vs Drugs Therapy	0	Some concerns	Low risk	No concerns	No concerns	No concerns	No concerns	Moderate quality
Dance vs Pilates	0	Some concerns	Low risk	No concerns	Some concerns	No concerns	No concerns	Moderate quality
Dance vs Relaxation	0	Some concerns	Low risk	No concerns	Major concerns	No concerns	No concerns	Low quality
Dance vs Resistance Training	0	Some concerns	Low risk	No concerns	Some concerns	No concerns	No concerns	Moderate quality
Dance vs Sham	0	Some concerns	Low risk	No concerns	No concerns	No concerns	No concerns	Moderate quality
Dance vs Virtual Reality	0	Some concerns	Low risk	No concerns	No concerns	No concerns	No concerns	Moderate quality
Education vs Flexibility	0	Some concerns	Low risk	No concerns	Some concerns	No concerns	No concerns	Moderate quality
Education vs Functional Training	0	Some concerns	Low risk	No concerns	Some concerns	No concerns	No concerns	Moderate quality
Education vs Drugs Therapy	0	Some concerns	Low risk	No concerns	Major concerns	No concerns	No concerns	Low quality
Education vs Pilates	0	Some concerns	Low risk	No concerns	Major concerns	No concerns	No concerns	Low quality
Education vs Relaxation	0	Some concerns	Low risk	No concerns	Major concerns	No concerns	No concerns	Low quality
Education vs Resistance Training	0	Some concerns	Low risk	No concerns	No concerns	Some concerns	No concerns	Moderate quality

Comparison	Trials	Within study bias	Reporting bias	Indirectness	Imprecision	Heterogeneity	Incoherence	Confidence rating
Education vs Sham	0	Some concerns	Low risk	No concerns	Some concerns	Some concerns	No concerns	Moderate quality
Education vs Virtual Reality	0	Some concerns	Low risk	No concerns	Major concerns	No concerns	No concerns	Low quality
Flexibility vs Functional Training	0	Some concerns	Low risk	No concerns	No concerns	No concerns	No concerns	Moderate quality
Flexibility vs Mixed Exercise	0	Some concerns	Low risk	No concerns	Some concerns	No concerns	No concerns	Moderate quality
Flexibility vs Drugs Therapy	0	Some concerns	Low risk	No concerns	Some concerns	No concerns	No concerns	Moderate quality
Flexibility vs Relaxation	0	Some concerns	Low risk	No concerns	Some concerns	No concerns	No concerns	Moderate quality
Flexibility vs Resistance Training	0	Some concerns	Low risk	No concerns	No concerns	No concerns	No concerns	Moderate quality
Flexibility vs Sham	0	Some concerns	Low risk	No concerns	Major concerns	No concerns	No concerns	Low quality
Flexibility vs Virtual Reality	0	Some concerns	Low risk	No concerns	Some concerns	No concerns	No concerns	Moderate quality
Functional Training vs Mixed Exercise	0	Some concerns	Low risk	No concerns	Major concerns	No concerns	No concerns	Low quality
Functional Training vs Drugs Therapy	0	Some concerns	Low risk	No concerns	Some concerns	No concerns	No concerns	Moderate quality
Functional Training vs Pilates	0	Some concerns	Low risk	No concerns	Some concerns	No concerns	No concerns	Moderate quality
Functional Training vs Relaxation	0	Some concerns	Low risk	No concerns	Major concerns	No concerns	No concerns	Low quality
Functional Training vs Resistance Training	0	Some concerns	Low risk	No concerns	Some concerns	Some concerns	No concerns	Moderate quality
Functional Training vs Sham	0	Some concerns	Low risk	No concerns	No concerns	No concerns	No concerns	Moderate quality
Functional Training vs Virtual Reality	0	Some concerns	Low risk	No concerns	Some concerns	No concerns	No concerns	Moderate quality
Mixed Exercise vs Drugs Therapy	0	Some concerns	Low risk	No concerns	Some concerns	No concerns	No concerns	Moderate quality
Mixed Exercise vs Pilates	0	Some concerns	Low risk	No concerns	Major concerns	No concerns	No concerns	Low quality
Mixed Exercise vs Relaxation	0	Some concerns	Low risk	No concerns	Major concerns	No concerns	No concerns	Low quality

Comparison	Trials	Within study bias	Reporting bias	Indirectness	Imprecision	Heterogeneity	Incoherence	Confidence rating
Mixed Exercise vs Resistance Training	0	Some concerns	Low risk	No concerns	Some concerns	No concerns	No concerns	Moderate quality
Mixed Exercise vs Sham	0	Some concerns	Low risk	No concerns	Some concerns	No concerns	No concerns	Moderate quality
Mixed Exercise vs Virtual Reality	0	Some concerns	Low risk	No concerns	Major concerns	No concerns	No concerns	Low quality
Drugs Therapy vs Pilates	0	Some concerns	Low risk	No concerns	Major concerns	No concerns	No concerns	Low quality
Drugs Therapy vs Relaxation	0	Some concerns	Low risk	No concerns	Some concerns	No concerns	No concerns	Moderate quality
Drugs Therapy vs Resistance Training	0	Some concerns	Low risk	No concerns	No concerns	No concerns	No concerns	Moderate quality
Drugs Therapy vs Virtual Reality	0	Some concerns	Low risk	No concerns	Some concerns	No concerns	No concerns	Moderate quality
Pilates vs Relaxation	0	Some concerns	Low risk	No concerns	Major concerns	No concerns	No concerns	Low quality
Pilates vs Resistance Training	0	Some concerns	Low risk	No concerns	No concerns	Some concerns	No concerns	Moderate quality
Pilates vs Sham	0	Some concerns	Low risk	No concerns	Some concerns	No concerns	No concerns	Moderate quality
Pilates vs Virtual Reality	0	Some concerns	Low risk	No concerns	Major concerns	No concerns	No concerns	Low quality
Relaxation vs Sham	0	Some concerns	Low risk	No concerns	Some concerns	No concerns	No concerns	Moderate quality
Relaxation vs Virtual Reality	0	Some concerns	Low risk	No concerns	Some concerns	No concerns	No concerns	Moderate quality
Sham vs Resistance Training	0	Some concerns	Low risk	No concerns	No concerns	No concerns	No concerns	Moderate quality
Resistance Training vs VR	0	Some concerns	Low risk	No concerns	No concerns	No concerns	No concerns	Moderate quality
Sham vs Virtual Reality	0	Some concerns	Low risk	No concerns	Some concerns	No concerns	No concerns	Moderate quality
